# Birth preparedness and complication readiness among women and couples and its association with skilled birth attendance in rural Bangladesh

**DOI:** 10.1371/journal.pone.0197693

**Published:** 2018-06-07

**Authors:** Sajia Islam, Janet Perkins, Md. Abu Bakkar Siddique, Tapas Mazumder, Mohammad Rifat Haider, Mohammad Masudur Rahman, Cecilia Capello, Dewan Md. Emdadul Hoque, Carlo Santarelli, Shams El Arifeen, Ahmed Ehsanur Rahman

**Affiliations:** 1 Maternal and Child Health Division, International Centre for Diarrhoeal Disease Research, Bangladesh (icddr,b), Dhaka, Bangladesh; 2 Health Department, Enfants du Monde, Geneva, Switzerland; 3 Department of Health Promotion, Education and Behavior, Norman J Arnold School of Public Health, University of South Carolina, Columbia, South Carolina, United States of America; TNO, NETHERLANDS

## Abstract

**Introduction:**

Despite remarkable progress in maternal and neonatal health over past two decades, maternal and neonatal mortality in Bangladesh remain high, which is partially attributable to low use of skilled maternal and newborn health (MNH) services. Birth preparedness and complications readiness (BCPR) is recommended by the World Health Organization and by the Government of Bangladesh as a key intervention to increasing appropriate MNH services. This study aims to explore the status of BPCR in a hard-to-reach area of rural Bangladesh and to demonstrate how BPCR practices is associated with birth in the presence of a skilled birth attendant.

**Methods:**

Data was collected using multistage cluster sampling-based household survey in two sub-districts of Netrokona, Bangladesh in 2014. Interviews were conducted among women with a recent birth history in 12-months and their husbands. Univariate, bivariate, and multivariable analysis using Stata 14.0 were performed from 317 couples.

**Results:**

Mean age of respondents was 26.1 (SD ± 5.3) years. There was a significant difference in BPCR practice between women and couples for identification of the place of birth (84% vs. 75%), identification of a birth attendant (89% vs.72%), arranging transport for birth or emergencies (20% vs. 13%), and identification of a blood donor (15% vs. 8%). In multivariable analysis, odds of giving birth in presence of a skilled birth attendant consistently increased with higher completeness of preparedness (OR 3.3 for 3–5 BPCR components, OR 5.5 for 4–5 BPCR components, OR 10.4 for all 5 BPCR components). For different levels of completeness of BPCR practice, the adjusted odds ratios were higher for couple preparedness comparatively.

**Conclusions:**

BPCR is associated with birth in the presence of a skilled attendant and this effect is magnified when planning is carried out by the couple. Interventions aiming to increase BPCR practices should not focus on women only, as involving the couple is most likely lead to positive care-seeking practices.

## Background

Despite global progress, 303,000 women continue to die each year worldwide due to causes related to pregnancy and childbirth. Ninety-nine percent of these deaths, nearly all preventable, occur in low- and middle-income countries [1]. Between 1990 and 2015 the global maternal mortality ratio (MMR) decreased by 45%, well below the three-quarters reduction which was targeted by the Millennium Development Goal (MDG) 5 [2]. Over this same period, child mortality decreased by 53% [3]. However, among the under-5 age group, neonatal mortality rate (NMR) proved to be the most resistant to reduction, and in every region of the world neonatal mortality now accounts for a larger proportion of under-5 mortality than it did in 1990 [3].

While Bangladesh has made remarkable strides in reducing maternal and neonatal death over the past two decades, the progress was not sufficient for the country to achieve MDG-5 target [3] and MMR still stands at 176 per 100,000 live births[1]. With an estimated NMR of 23 per 1,000 live births in 2015, newborn mortality now accounts for 61% of all under-5 deaths in Bangladesh [3].

In the new era of the Sustainable Development Goals (SDGs), maternal and newborn health (MNH) has been retained as a priority. The 2030 UN Agenda aims to achieve a MMR of ≤70 deaths per 100,000 live births, and NMR of ≤12 per 1,000 live births for every country. Most countries, including Bangladesh, have declared their commitment to achieve these targets, which will require countries to maximize their efforts to ensure access to and utilization of MNH care [4]. In addition to continued investment in increasing availability, readiness and quality of MNH services, birth preparedness and complication readiness (BPCR) can play a significant role in overcoming the barriers related to access to and utilization of skilled MNH care [5–11].

Recognized globally as a key approach for promoting the use of skilled MNH care for women and newborns, BPCR is the process through which women and families plan actions in anticipation of birth and possible obstetric and neonatal emergencies [12]. It has long been considered instrumental in improving the health of women and newborns, and was included by the World Health Organization (WHO) as an integral component of antenatal care (ANC) by the early 2000s [13]. The importance of BPCR has recently been reiterated by WHO as a recommended priority health promotion intervention for MNH [14]. Even in low- and middle-income countries with poorly functioning health systems, increased preparedness for birth and complications allows women and their families to anticipate potential delays, and ensure skilled care for birth and timely use of appropriate facility for complications [14].

While BPCR is recommended as an integrated element of ANC contacts which are generally one-to-one interactions between women and health services providers, the benefits of BPCR practice are likely to be optimized when it is undertaken as a joint process between women and household decision-makers, and particularly male partners [15–18]. Indeed, WHO recommends involving male partners in MNH as a strategy for increasing women’s access to skilled care during pregnancy, around the time of birth and in the case of complications [14]. As men are critical gate-keepers in many societies, it is assumed that their involvement in BPCR can help to ensure that women are able to follow through with the plan which has been prepared in advance; however, little evidence exists regarding the benefit of male involvement in BPCR.

In Bangladesh, utilization of MNH care remains low: less than half (42%) of births are attended by a skilled birth attendant [19]. The recently revised Maternal Health Strategy of Bangladesh 2017 envisions achieving very high national coverage of skilled birth attendance, i.e. 93% by 2030 [20]. BPCR has been prioritized as a key approach to achieve this target and is included by the Bangladesh Maternal Health Strategy as an essential intervention to be promoted during the antenatal period. The current Maternal Health Strategy of Bangladesh emphasizes promotion of the following five core components of BPCR: identifying the place of birth, identifying a birth attendant, arranging transport, saving money for emergencies and identifying a potential blood donor. The maternal health strategy recommends that all facility based health service providers should counsel pregnant women on these BPCR practices during routine ANC contacts. Community-based health workers are also instructed to promote BPCR practices during their routine domiciliary visits. The new strategy also recommends increasing the quality and effectiveness of BPCR through innovative and multi-sectoral approaches.

To date, some evidence exists regarding the status of BPCR practice in Bangladesh [5], however there is no evidence correlating how these practices contribute to the use of skilled MNH care or the value of male involvement in BPCR in this context. Our study aims to explore the status of BPCR in a hard-to-reach area of rural Bangladesh and to demonstrate how practices related to BPCR among women and among couples contribute to ensuring birth in the presence of a skilled birth attendant. Finally, we assess the added value of couples’ joint BPCR planning over women’s planning.

## Materials and methods

### Study design and settings

A community-based, cross-sectional household survey was conducted in hard-to-reach two sub-districts, Barhatta and Kalmakanda, of Netrokona district in Bangladesh in 2014. Netrokona is located approximately 200 kilometres north of Dhaka, the capital of Bangladesh. It is one of the 14 lowest performing districts of Bangladesh in terms of newborn and child mortality rates [21]. Netrokona’s landscape is dominated by four major rivers and abundant wetlands known as *haors*. Agriculture and fishing are the primary sources of income. The sub-district of Kalmakanda has a land area of 377 square kilometres and a total population of around 272,000. Barhatta covers 220 square kilometres of land with a total population of approximately 180,000.

Kalmakanda was included in the study as it had been selected as the implementation site of a programme focusing on health promotion and community engagement actions to improve MNH. This programme was implemented by ‘PARI Development Trust’, a local non-governmental organization (NGO), in collaboration with the Directorate General of Health Services (DGHS) and the Directorate General of Family Planning (DGFP)- branches of the Ministry of Health and Family Welfare (MOHFW). Enfants du Monde (EdM), a Geneva-based NGO provided technical support to the programme and icddr,b (an international health research institute based in Bangladesh) conducted evaluation independently. One of the key planned interventions was the promotion of BPCR, which was initiated following the baseline study. Barhatta, an adjacent sub-district to Kalmadanda, was selected as the control site.

### Study population

Eligible women who gave birth within the 12-month period preceding the survey and their husbands/partners were included in the study.

### Sample size

The sample size was calculated for evaluating the effectiveness of the health promotion and community engagement intervention package on giving birth in the presence of a skilled birth attendant. As planned, the intervention package was delivered in one of the above mentioned sub-districts (Kalmakanda) and study adopted a quasi-experimental design with a comparison sub-district (Barhatta).

As baseline assumption for sample size calculation, we considered the estimates from the BMMS 2010 report (the national survey reporting district-specific estimates in Bangladesh) where the coverage of birth with a skilled birth attendant in Netrokona was reported to be 15.6%. We assumed a minimum of a 10-percentage point (absolute) increase in coverage of skilled birth attendance between baseline and endline. We also wanted to ensure a higher sample from the intervention site (intervention: comparison = 1.5:1). The unadjusted sample size was 404 from the intervention site and 269 from the comparison site at 80% power and 5% error probabilities. The sample size was then adjusted for design effect/cluster effect (1.25) and non-response/loss to follow up (5%). The final sample size was 425 from the intervention site and 283 from the comparison site at baseline and at endline. We present here the findings from the baseline survey. At baseline (conducted in 2014), we interviewed 725 women with a recent birth history at baseline (444 from the intervention site and 281 from the comparison site). We approached all husbands of the women and conducted 317 interviews successfully. Information from 317 wife-husband dyads is presented in this paper.

### Sampling

This study used a multistage cluster sampling to select eligible respondents. In the first stage, four unions (the smallest administrative unit of Bangladesh with an average population of 30,000) were randomly selected from each of the selected sub-districts. In the second stage, four clusters (average population of approximately 1,000) were selected from each union using the probability proportional to size (PPS) sampling technique. All eligible respondents were included from the selected clusters.

### Data collection

In the first stage of sampling, a sketch map was drawn for each of the selected clusters representing boundaries, landmarks and bari (extended household) locations. All households and women who had a birth outcome in the 12 months preceding the survey were enumerated and listed. In the second stage, separate structured questionnaires were used for interviewing all eligible women and their husbands (S1 Table). The questions were adopted from the Bangladesh Demographic and Health Survey (BDHS) 2011, Bangladesh Maternal Mortality Survey (BMMS) 2010 and other relevant studies [19, 22]. Women and their husbands were interviewed separately by different groups of data collectors. The questionnaire started with questions regarding personal and socioeconomic information such as age, education level, marital status and employment status followed by questions related to utilization of routine and emergency obstetric care. Data related to BPCR including identifying a birth place, identifying a birth attendant, saving money for emergencies, arranging transportation to reach the health facility, and arranging a potential blood donor were collected. Information on the extent and roles of spouses’ involvement in BPCR were also collected. For quality assurance, data collection instruments were pre-tested in non-selected clusters of the selected unions. Interviewers were locally recruited to facilitate the data collection processes as they would be familiar with the local context, culture and dialect. Experienced facilitators, trainers and field supervisors trained the data collectors.

### Data analysis

Data was analysed using Stata 14.0 (StataCorp. 2015. Stata Statistical Software: Release 14. College Station, TX: Stata Corp LP). Women’s education, husbands’ education and women’s age were converted to categorical variables from continuous variables. Religion was re-categorised to Muslim and “other”, as all other religions had smaller frequencies. The household asset score was generated using the principal component analysis [23, 24]. Then the asset scores were used to generate wealth quintiles (five categories).

BPCR practices among women and couples were considered as the main explanatory factors. Birth in presence of a skilled birth attendant was considered to be the primary outcome of interest. Regarding BPCR practices, the following five components were included in the analysis as per the Bangladesh Maternal Health Strategy: identifying the place of birth, identifying a birth attendant, arranging transport, saving money for emergencies and identifying a potential blood donor. Couple preparedness for individual components of BPCR was considered when both the women and her husband/partner reporting to be prepared for that component. Complete BPCR was defined as having planned for 3–5 components of BPCR. We have also presented the completeness for 4–5 components and for all 5 components. Good BPCR practices were defined as the following: planning to give birth in a health facility or at home with a skilled birth attendant, discussing BPCR components with a health care provider and discussing BPCR components with the spouse. Regarding the primary outcome of interest, birth was considered to be in the presence of a skilled birth attendant if it occurred in a health facility or if a skilled birth attendant was present during a home birth (which is recognized as a legitimate option by the Ministry of Health and Family Welfare (MOHFW).

We used descriptive statistics (both univariate and bivariate) to describe BPCR practices among women and couples. Proportion test (z test) was conducted to see the statistically significant difference of BPCR practice between women and couples.

The associations between individual components of BPCR practice and birth in the presence of a skilled birth attendant were assessed using multiple logistic regressions after adjusting for known confounders (women age, women’s educations, husband’s education, religion, wealth quintile) when they have showed significant associations in bivariate analysis (S2 Table). Any significant association is reported at p-value<0.05.

### Ethical approval and consent to participate

Ethical approval to conduct the study was obtained from the Institutional Review Board of icddr,b (Protocol Number: PR 14024). All participants interviewed in our survey were married and had a birth outcome in twelve months preceding the survey. As per the IRB recommendations, any married woman with a child can give consent for interviews in the Bangladeshi context (irrespective of age). Moreover, in the Bangladeshi context women move to their husbands’ residents (home) after marriage. Parents are not considered as the guardian of women after marriage irrespective of their age. In addition, husbands were informed regarding the interviews. Prior to interviews, participants were informed of the voluntary nature of their participation and their right to withdraw at any time during the study. They were also informed that refusal to participate in the study would not involve any penalty. Written and informed consent was obtained from each participant once they were fully informed. Privacy, anonymity and confidentiality of the participants were strictly maintained during data collection and analysis.

## Findings

Table 1 presents the background characteristics of all of the respondents included in the survey. Female respondents had an age range of 15–49 years with a mean age of 26.1 (SD ±5.3) years. Most of the respondents lived in rural areas. Half owned land (50%) and more than 80% were in possession of a mobile phone. More than half of the women had no education or did not complete primary education (five years of schooling). Around two-thirds of the husbands had no education or did not complete primary education. Only 7% had completed secondary education. Few women (3%) were involved in income-generating activities.

**Table 1:** Background characteristics of respondents (women and their husbands) (N = 317).

Background characteristics	%	n
**Women’s age**		
15–24 years	42.1	133
25–34 year	50.9	161
35+ years	7.0	22
**Women’s education**		
No Education/Primary incomplete (0–4 years)	55.4	176
Primary complete to secondary incomplete (5–9 years)	38.9	123
Secondary complete or higher (10+ years)	5.7	18
**Husbands’ education**		
Primary incomplete (0–4 years)	65.1	206
Primary complete to secondary incomplete (5–9 years)	28.1	89
Secondary complete or higher (10+ years)	6.8	22
**Women’s involvement in income generating activities**	3.0	10
**Housing possession /household assets**		
Television	12.1	38
Mobile	81.9	260
Land	49.5	157
**Religion**		
Muslim	91.4	290
Others (Hindu/Christian etc.)	8.6	27

Table 2 presents the practice among women and couples as per different components of BPCR. More than 80% women identified a place of birth or a birth attendant in advance. However, preparedness across other components was much lower. Less than half (42%) of women reported having saved money from emergencies. Preparedness related to arranging transportation for birth or emergencies and identifying a potential blood donor was low among women and couples. There was a significant difference (p<0.05) in BPCR practice between women and couples for identification of the place of birth (84% vs. 75%), identification of a birth attendant (89% vs.72%), arranging transport for birth or emergencies (20% vs. 13%), and identification of a blood donor (15% vs. 8%).

**Table 2:** Components of BPCR practices among women and couples.

Components of BPCR	Women (N = 317) %	Couples (N = 317) %	P-value
Identified the place of birth	83.9	74.8	0.004
Identify a birth attendant	88.3	71.9	0.000
Arrange transport for birth or emergency	20.2	12.6	0.010
Save money for emergency	42.3	38.2	0.292
Arrange/identify a potential blood donor(s)	14.5	8.2	0.012

**Fig 1:**
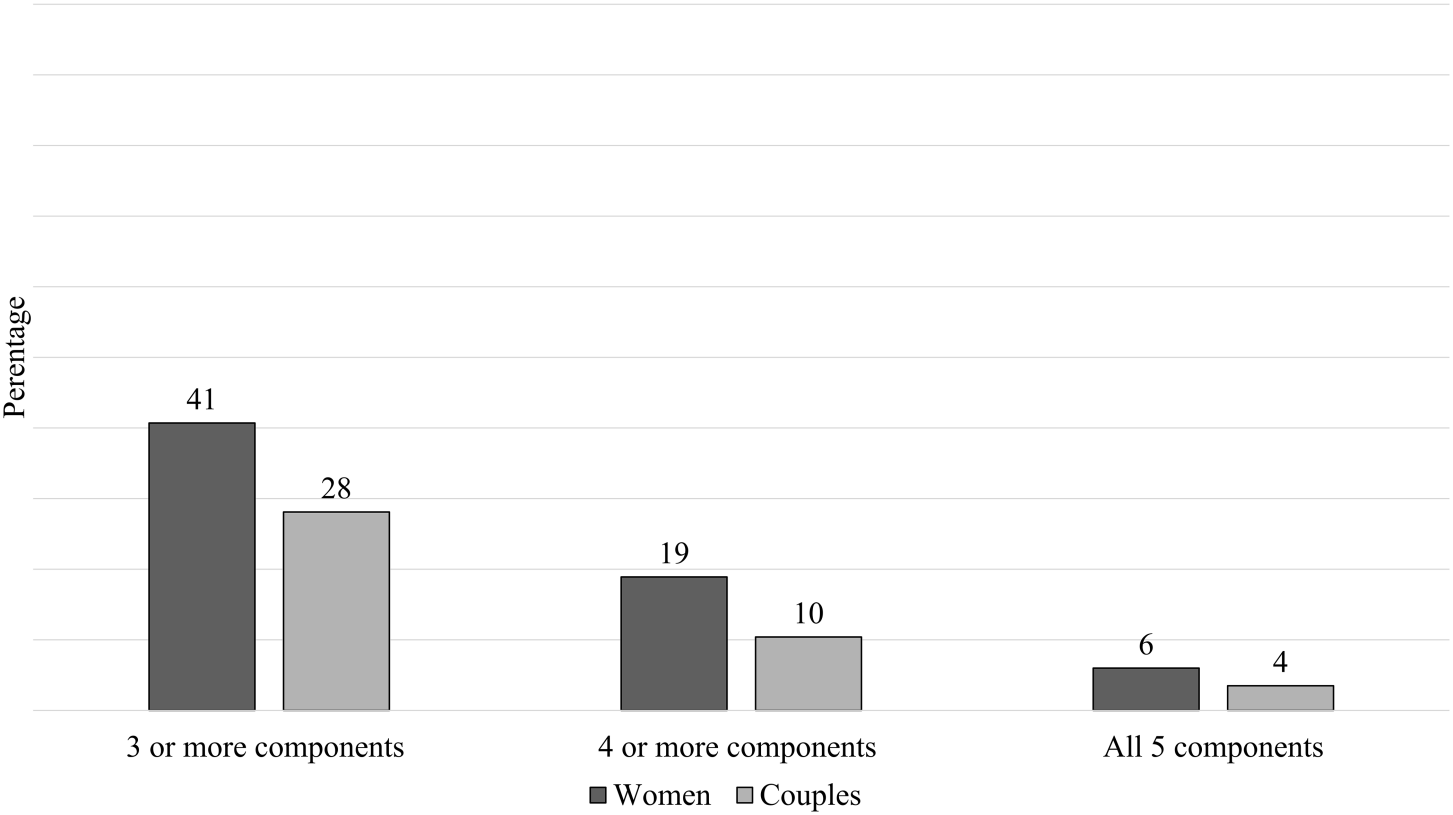
Completeness of BPCR practices among women (n = 317) and couples (n = 317).

Fig 1 presents the completeness of BPCR practices among women and couples. Less than half (41%) of women reported to have prepared three or more components of BPCR, while only 28% couples reported doing so (p = 0.001). Less than one-fifth of women had practiced four or more components of BPCR, whereas only one-tenth of the couples had reported such preparation (p = 0.003). Only 6% women reported having planned across all five components of BPCR, which was lower (3.5%) among couples although the difference was not statistically significant (p = 0.139).

Table 3 summarizes the different indicators to reflect BPCR good practices among women and couples. Although over 80% of women and three-quarters of couples reported having identified a place of birth in advance, very few planned to give birth in a health facility (6% among women and 5% among couples). Among those who planned to give birth at home, only 4% planned to have an SBA present. Only one-third of women reported having discussed BPCR in general with a health worker; whereas only 13% of couples reported having had this discussion with a health worker. Approximately one-quarter of the women reported that they had not discussed BPCR in general with their spouse.

**Table 3:** BPCR good practices, by women and couples.

BPCR good practices	Women (N = 317) %	Couples (N = 317) %	P value
Planned to give birth in health facility	6.3	4.7	0.384
Planned to give birth at home with an SBA	3.8	*data not available	
Discussion with health care provider	27.8	12.9	0.000
Discussion with spouse	78.9	66.9	0.001
Discussed with both health care provider and spouse	24.9	12.0	0.000

*the question was not asked to the husband

The proportion of women who gave birth in health facility or with presence of a skilled birth attendant at home was 16.5%. Table 4 presents the relationship between BPCR practice and birth in the presence of a skilled birth attendant (either facility birth or home birth with a medically-trained provider). An insignificant association was found between identifying a place of birth or birth attendant in advance and ultimately giving birth in the presence of a skilled birth attendant. In contrast, a significant association (p<0.05) was observed between the remaining three components of BPCR (arranging transport, saving money and identification of a blood donor) and giving birth with in the presence of a skilled birth attendant. The odds of giving birth in the presence of a skilled birth attendant was found to be higher with couple preparedness across every component of BPCR compared to preparedness among women after adjusting for covariates and confounders.

**Table 4:** Relationships between BPCR practice as per different components and birth in the presence of a skilled birth attendant, by women and couples.

Components of BPCR	Birth with a skilled birth attendant						
	Women (N = 317)				Couples (N = 317)		
		%	UOR (95% CI)	AOR (95% CI)	%	UOR (95% CI)	AOR (95% CI)
Identified the place of birth	(Ref) No	15.7	1.1 (0.5,2.4)	0.9 (0.4,2.2)	15	1.2 (0.6,2.3)	1 (0.5,2.2)
	Yes	16.5			16.9		
Identified a birth attendant	(Ref) No	27	0.5 (0.2,1.1)	0.4* (0.2,0.95)	16.9	1 (0.5,1.8)	0.8 (0.4,1.7)
	Yes	15			16.2		
Arranged transport for emergency	(Ref) No	11.9	3.9* (2.1,7.4)	3.1* (1.5,6.3)	13	4.5* (2.2,9.2)	3.4* (1.5,7.8)
	Yes	34.4			40		
Saved money for emergency	(Ref) No	6.6	6.1* (3.0,12.1)	4.1* (1.9,8.7)	6.6	6.7* (3.4,13.2)	4.5* (2.1,9.5)
	Yes	29.9			32.2		
Arranged/identified a potential blood donor(s)	(Ref) No	10	10.8* (5.3,21.7)	6.1* (3.1,15.2)	12	13.8* (5.7,33.4)	6.8* (2.5,18.8)
	Yes	54.3			65.4		

UOR: Unadjusted Odds Ratio; AOR: Adjusted Odds Ratio,

* p<0.05

AOR adjusted for women age, women education, husband education, religion and wealth quintile

**Fig 2:**
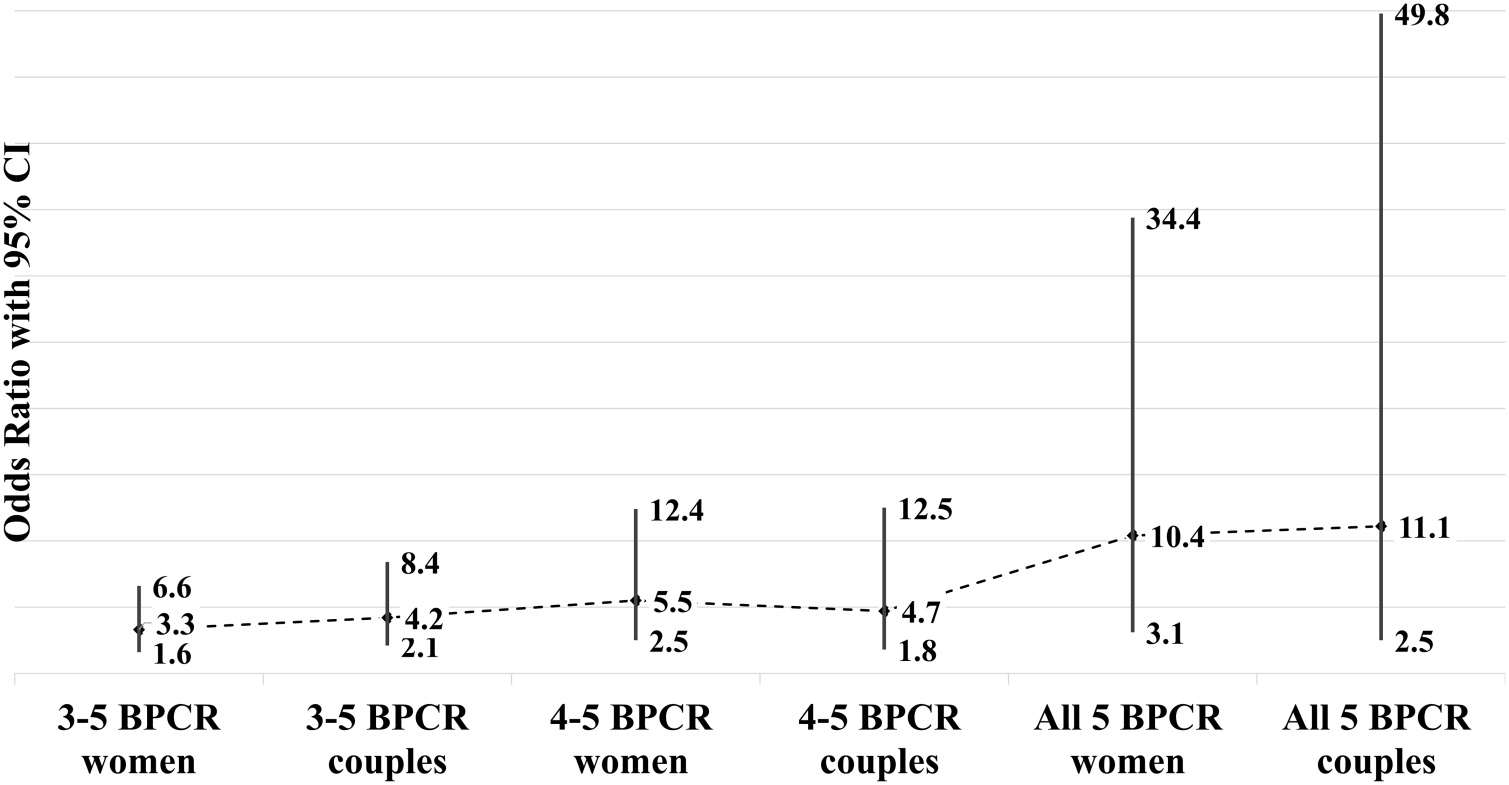
Associations (adjusted odds ratio with 95% CI) between completeness of BPCR and birth in the presence of a skilled birth attendant, by women and couples.

Fig 2 illustrates the relationship between the completeness of BPCR practice and birth in the presence of a skilled birth attendant through adjusted odds ratios. The odds of giving birth in the presence of a skilled birth attendant was found to be 3.3 times higher among women who had practiced 3–5 components BPCR compared to those who practiced fewer than three components of BPCR. The odds of birth in the presence of a skilled birth attendant consistently increased with higher completeness of preparedness (OR 5.5 for 4–5 BPCR components, OR 10.4 for all 5 BPCR components). For different levels of completeness of BPCR practice, the adjusted odds ratios were higher for couple preparedness than preparedness among women. Around 90% of women who planned to deliver in a health facility, followed through on it (AOR 53.8, CI 10.6–269.4). This increased to 100% when the plan was made jointly by the couple (not presented in the tables).

## Discussion

Preparing for birth and potential complications has been identified globally and at country level as a key strategy and intervention for ensuring birth in the presence of a skilled birth attendant and improving the health of women and newborns [12, 14, 20]. Our findings indicate that BPCR practices are low in Netrokona. However, complete BPCR is positively associated with giving birth in the presence of a skilled birth attendant. Moreover, our findings indicate that this effect is magnified when husbands are involved in the process and planning is carried out by the couple.

Not surprisingly, we find that BPCR practice in Netrokona is insufficient. Netrokona is among the lowest performing districts, reflected in basic development indicators [21], and this study was carried out in some of the remote and hard to reach areas of the district. Completeness of BPCR practice was found to be low, with fewer than half (40%) of women preparing at least three components. The planning among women seems to be similar to what has been observed in Africa and in India; however, different studies employ varying definitions for assessing the completeness of planning, rendering cross-country comparisons difficult. For instance, some studies group ANC attendance as a BPCR component, while others only consider planning for the birth place to have been completed if facility birth was planned [25–28]. In this study, we considered this component to have been completed regardless of the place selected. This was done intentionally in order respect that the decisions taken by participants, and women in particular, as well as to reflect the local context., Home birth is deemed acceptable in Bangladesh, provided that the birth is assisted by a skilled birth attendant. A cadre of community-based skilled birth attendants has been trained and deployed throughout the country to serve this purpose, though coverage remains minimal [29]. When taking these variations into account, birth planning in Netrokona seems to be generally lower than in many of the other contexts reported on. However, the level of planning seems to be consistent with other hard to reach areas of Bangladesh [5].

Among our study participants, we observe wide variations in planning across the five key components. Identifying a birth place and a birth attendant is the most common practice, with the majority of respondents planning for these components. However, optimal planning of a birth place and a birth attendant remains the exception among our participants, as few women and couples planned for birth in a health facility or arranged for a home birth in presence of a skilled birth attendant. This may be due to the lack of availability of skilled MNH care or to religious and cultural preferences toward giving birth at home [30]. Such sub-optimal planning is not likely to contribute to improved care-seeking behaviours, nor improved MNH outcomes [31–33].

Beyond these components, saving money for potential costs related to obstetric and neonatal emergencies was the next most common BPCR practice among our participants, followed by arranging transportation. Identification of a potential blood donor remains the most neglected component of BPCR according to our findings, with only 15% of women identifying a blood donor, and less than 10% of couples making such preparations. Our findings are generally consistent with other studies conducted in Africa and India which have found saving money to be a relatively common BPCR practice, followed by arranging transportation. Identification of a potential blood donor is consistently the rarest component taken into consideration [26, 34–37]. It may be that this component is the least intuitive of all components and therefore requires more contact with the health service providers for women and families to be sensitized to and plan for this.

Another of our key findings is that intention to deliver in a facility was highly predictive of giving birth in a facility. Though the proportion of women who planned to give birth in a facility was small, almost all who made this plan followed through on it. This finding contrasts with a study conducted in Tanzania, which found that nearly 40% of women who had planned a facility birth were not able to follow through on this plan [38]. This may indicate that the barrier in deciding to seek care is among the most important in Netrokona, and that once the decision has been made, women and families are able to overcome barriers related to reaching health services and accessing services once a health facility has been reached [39]. An alternate explanation could be access to health facility acts as a confounder; i.e., those who planned to give birth in a health facility had greater access to the health facility, and eventually delivered in a health facility. We did not have enough data to control for this confounder in our multiple logistic regression models.

In terms of male involvement, studies throughout Africa and Asia have demonstrated the involvement of men in birth preparedness and complication readiness to be context specific, with low overall involvement in some regions and high in others, with wide variation across components [16]. In this study we find that across all components of BPCR, with the exception of saving money, women had significantly higher levels of planning, compared to couples. These findings are consistent with other studies which have also found men to be most involved in planning the financial component of BPCR [40].

The global evidence regarding the impact of BPCR practice on birth in presence of a skilled birth attendant has been mixed, with some studies finding a positive association between BPCR and skilled care, and others failing to find this relationship [41, 42]. While BPCR planning remains low in Netrokona, our findings indicate that in general comprehensive planning is predictive of birth in presence of a skilled birth attendant. Women who arranged transport for birth and emergencies, saved money and identified a blood donor were consistently more likely to give birth in presence of a skilled birth attendant. However, this same relationship was not observed for women identifying a place of birth and a birth attendant in advance. Our analysis suggests that identifying ‘any place of birth’ or ‘any person for conducting the child birth’ is not predictive of delivering in presence of a skilled birth attendant. However further analysis revealed that planning to deliver in a health facility or in presence of a skilled birth attendant at home is predictive of finally delivering with skilled birth attendant. This implies that the MNH programs and the concerned health care providers should change their approach related to BPCR counselling. Instead of simply promoting the identification of ‘any place of birth’ or ‘any person for conducting the childbirth’, health care providers should seek to understand women’s and families reasons for selecting a home birth or birth attendance by an unskilled provider. Then they can more appropriately counsel pregnant women and their husbands regarding the importance and advantages of facility birth or arranging skilled birth attendant to present during a home birth. This may be an important programmatic lesson. It is also possible that planning for the other three components requires a greater degree of anticipation, and respondents who had planned for these might had a greater degree of seriousness regarding their plan and were therefore more likely to seek out skilled care.

While women’s planning had a predictive value of birth with a skilled birth attendant, joint planning within the couple magnified this effect across all components, BPCR completeness and BPCR good practices. This is particularly striking with regard to completeness of planning: women were significantly more likely to give birth in presence of a skilled birth attendant when the couples planned for three or four components of BPCR compared to when women undertake this planning. In addition, couples planning over independent planning by the women increased the odds of giving birth in the presence of a skilled birth attendant for the individual BPCR components and for good practices. This is consistent with a study conducted in Uganda looking at decision-making on birth preparedness planning and its influence on giving births in the presence of a skilled birth attendant among women. This study found that women who made joint decisions with spouses or other close persons (e.g. mothers-in-law, friends) were more likely to give birth in the presence of a skilled birth attendant compared to when women undertook this planning alone [43].

The finding that couples’ planning has an added value over woman planning is not surprising, particularly in a context such as that found in Netrokona, where women’s autonomy and decision-making remain limited [44]. Women are often reliant on others within the household, particularly men, to make decisions, including decisions related to MNH. Therefore, it stands to reason that even if a woman prepares a plan independently, she may be limited in her capacity to follow through on this plan if the choice has not been agreed upon within the couple.

Our study suggests that BPCR contributes to birth in the presence of a skilled birth attendant and that having men engaged in BPCR increases its potential benefits. The majority of studies on BPCR have been conducted in the context of Africa. Future research on BPCR should be conducting in Asian and other contexts to more fully understand the relationship between BPCR and use of skilled health services in these regions of the world. Furthermore, future research should seek to better understand how best to promote BPCR in such resource poor-setting and to include men in a way which contributes to the health and well-being of women and children.

## Limitations

As this was a cross-sectional survey, we cannot establish causality between BPCR and the outcomes assessed. In addition, recall bias may be a limitation as respondents who had a birth in presence a skilled birth attendant may recall the practices related to BPCR more than those who did not give birth in presence a skilled birth attendant. However, we tried to minimize the recall bias by asking the questions related to BPCR before the questions related to utilization of MNH care. Moreover, some of the respondents may not have accurately remembered the details which they were asked about. However, we expect the recall error to be similar between the two outcome groups, hence will not bias the findings. There is also the possibility of social desirability bias; we believe that this limitation was mitigated to some degree by recruiting local data collectors.

In addition, we know that other family members can also play an important role in the Bangladeshi context. However, we were unable to collect additional information regarding other family members in this survey. Future research should explore this additional element.

## Conclusion

Our findings suggest that BPCR is directly associated with giving birth with a skilled birth attendant, which is identified globally as a key intervention for saving the lives of women and newborns. Actions aiming to increase good BPCR practices should be prioritized, particularly in resource-poor, hard-to-reach settings such as Netrokona, where women are likely to face many challenges to accessing skilled care. Moreover, our results indicate that the benefits of the BPCR practice are maximized when it is more complete and when it is a joint process undertaken by the couple. Interventions aiming to increase BPCR practice should focus on ensuring complete preparedness. Moreover, they should move beyond focusing on women only and also target the couples as joint planning within the couple is most likely to lead to positive care-seeking practices.

## Supporting information

S1 Table (PDF)

S2 Table (DOCX)

S1 Dataset (DTA)
