# Supplementary material for: Birth preparedness and complication readiness among women and couples and its association with skilled birth attendance in rural Bangladesh
Source: PLoS One. 2018 Jun 7;13(6):e0197693. doi: 10.1371/journal.pone.0197693 (PMC5991697; doi:10.1371/journal.pone.0197693)
Supplement: S1 Table — (PDF) [file pone.0197693.s001.pdf]

IFC Framework Evaluation Baseline Survey 2014

Module I

	Name	Code
CLUSTER/UNION		□□
UPAZILA		□□
VILLAGE		□□□□
HOUSEHOLD ID		□□□□
WOMEN ID		□□□
ADDRESS (Location)		

সাক্ষাৎকার শুর‍ু হবার সময়: |___|___|:|___|___|

সাক্ষাৎকার শেষ করার সময় |___|___|:|___|___|

Interview summary

	Visit 1	Visit 2	Visit 3
Date	|__|__|-|__|__|-|__|__|	|__|__|-|__|__|-|__|__|	|__|__|-|__|__|-|__|__|
Interviewer's Name			
Result code*	|__|__|	|__|__|	|__|__|
Next Visit	|__|__|-|__|__|-|__|__|	|__|__|-|__|__|-|__|__|	

Final Visit	
Date	|__|__|-|__|__|-|__|__|
Interviewer's code	|__|__|__|
Result code	|__|__|
Total # of visits	|__|

	ফলাফল বৃত্তায়িত কর‍ুন		বর্তমান অবস্থা
	করতে হবে	করা হয়েছে	মন্তব্য
১ লা জানুয়ারী ২০১৩ সাল বা তার পরে গর্ভ ফলাফল আছে এমন মহিলার জন্য **Module II** (মা ও নবজাতকের যত্ন) পূরন করতে হবে কি?<br>Is it required to complete **Module II** (maternal and immediate newborn care) for the woman with a birth outcome since 01January-2013?	Y / N	Y / N	
১ লা জানুয়ারী ২০১৩ সাল বা তার পরে গর্ভ ফলাফল আছে এমন মহিলার স্বামীর জন্য **Module X**/HUSBAND AWARENESS MODULE পূরন করতে হবে কি?<br>Is it required to complete **Module III** / HUSBAND AWARENESS MODULE for women in the HH with a birth outcome since 01-January-2013?	Y / N	Y / N	

***Result Codes**

01. Interview Complete
02. Partial complete
03. Refused to give interview
04. Could not find the residence
05. The woman was not available at the date of interview
06. No woman found with pregnancy outcome since 01 January 2012
07. Others ______________________ |____|____|

Supervision	Name	Code	Date
Reviewed by FS		|___|___|	|___|___|-|___|___|-|___|___|
Checked by FRS		|___|___|	|___|___|-|___|___|-|___|___|
Reviewed by QC Team		|___|___|	|___|___|-|___|___|-|___|___|

Data Entered by	|___|___| |___|___|-|___|___|-|___|___|

Section A: Household Information

This Section contains some house hold information of the selected woman and other members of the same house-hold

	পরিবারের গঠন (বর্তমানে জীবিত ও গৃহের নিয়মিত বাসিন্দা):	পুরুষ (Male)		মহিলা (Female)	
A01	2 বছরের কম বয়সী শিশুর সংখ্যা ....<br>Number of U2 years children in the Household	|___|___|	…………..…	|___|___|	
A02	2 - 5 বছরের কম বয়সী শিশুর সংখ্যা ....<br>Number of children of age 2-up to 5 years	|___|___|	…………..…	|___|___|	
A03	5-15 বছরের কম বয়সী বালক / বালিকার সংখ্যা..................<br>Number of children/adolescents of age 5-up to 15 years	|___|___|	…………..…	|___|___|	
A04	পরিবারে 15- 49 বছর বয়স্ক সদস্য সংখ্যা ....<br>Number of adults of age 15-49 years	|___|___|	…………..…	|___|___|	
A05	15- 49 বছর বয়স্ক কখনও বিয়ে হয়েছিল এমন মহিলার সংখ্যা Number of ever-married women of age 15-49 yrs		…………..…	|___|___|	
A06	50 বছর বা তার উর্ধ্বে বয়সের সদস্য সংখ্যা ....<br>Number of adults of age 50+ years	|___|___|	…………..…	|___|___|	

Section B: Socio-Economic Information

No.	Questions and Filters	Coding Categories	Skip
B01	আপনার ঘরের/খানার সদস্যদের পানি পান করার প্রধান উৎস কি/কোথায়?<br>What is the main source of drinking water for members of your household?	পাইপের পানিঃ (Piped water)<br>বাড়ীর ভিতরে ট্যাপের (পাইপের) পানি (Piped inside dwelling).................................... 11<br>বাড়ীর বাহিরে ট্যাপের (পাইপের) পানি (Piped outside dwelling) .... 12<br>কুপের পানিঃ (Well water)<br>শ্যালো টিউবওয়েল (Shallow tubewell)................... 21<br>গভীর নলকূপ (Deep tubewell) .... 22<br>কুয়া (Surface well/other well) .... 23<br>ভূ-পৃষ্ঠের পানিঃ (Surface water)<br>পুকুর/খাল/বদ্ধ জলাশয়/হ্রদ/দীঘি/বিল/হাওড় (Pond/Tank/Lake)....................................... 31<br>নদী/ঝর্নার পানি (River Stream)............................ 32<br>বৃষ্টির পানি (Rain water) .... 41<br>অন্যান্য (Other)<br>________________________________ 97<br>(নির্দিষ্ট করুন)	

No.	Questions and Filters	Coding Categories	Skip
B02	আপনাদের কি ধরনের পায়খানা/ল্যাট্রিন এর ব্যবস্থা আছে?<br>What kind of toilet facility does your household have?	সেপটিক ট্যাংক/আধুনিক ল্যাট্রিন (Septic tank/Modern toilet) 11<br>গর্ত (পিট) টয়লেট/ল্যাট্রিন (Pit toilet/Latrine)<br>জলাবদ্ধ/¯-ve (স্যানেটারী) ল্যাট্রিন<br>(Water sealed/Slab latrine)............................ 21<br>গর্তের (পিট) ল্যাট্রিন (Pit latrine) .... 22<br>খোলা/ঝুলন্ত ল্যাট্রিন (Open/Hanging latrine).............. 23<br>ল্যাট্রিন নাই/ঝোপ ঝাড়/মাঠ (No facility/Bush/Field).......... 31<br>অন্যান্য (Others)<br>_______________________________ 97<br>(নির্দিষ্ট করুন)	
B03	আপনাদের ঘরের/খানার কোন সদস্যের এই জিনিসগুলো আছে কি?<br>[প্রত্যেকটি জিনিসের কথাই জিজ্ঞেস করুন]<br><br>How many of the followings are owned by your household?	**Yes No**<br>রেডিও (Radio).. 1..... 2<br>টেলিভিশন (Television) .... 1..... 2<br>টেপ রেকর্ডার (Tape recorder) .... 1..... 2<br>ভিসিপি/ভিসিআর/ডিভিডি (VCR/VCP/DVD player) .... 1..... 2<br>মোবাইল ফোন (mobile phone) .... 1..... 2<br>টেলিফোন (Land Telephone) .... 1..... 2<br>বৈদ্যুতিক পাখা (Electric Fan) .... 1..... 2<br>কম্পিউটার (Computer) .... 1..... 2<br>ফ্রিজ (Refrigerator) .... 1..... 2<br>ঘড়ি (Watch) .... 1..... 2<br>আলমারি (Almira)... 1..... 2<br>রান্নাঘরের তাকিয়া (Kitchen cabinet) .... 1..... 2<br>খাট/চৌকি (cot/bed)...................................... 1..... 2<br>টেবিল (Table) .... 1..... 2<br>চেয়ার (Chair)... 1..... 2<br>সোফা (Sofa).. 1..... 2<br>লেপ/কম্বল (Lep/Kombol/Blanket) .... 1..... 2<br>তোষক/জাজিম (Toshok/Jajim/Matress) .... 1..... 2<br>বাই সাইকেল (Bicycle) .... 1..... 2<br>মোটর সাইকেল/স্কুটার (Motor cycle/Scooter) .... 1..... 2<br>গরু/মহিষের গাড়ী (Animal drawn cart).................. 1..... 2<br>গাড়ি/মাইক্রোবাস (Car/Microbus) .... 1..... 2<br>মালবাহী গাড়ি/বাস (Truck/Bus) .... 1..... 2<br>ইঞ্জিন চালিত নৌকা (Motor-Boat)....................... 1..... 2<br>নৌকা (ইঞ্জিন ছাড়া) (Boat without motor)............... 1..... 2<br>রিক্সা/ভ্যান (Rickshaw/Rickshaw Van).................... 1..... 2	
B04	আপনাদের ঘরে কি বিদ্যুৎ আছে?<br>Do you have Electricity in your house?	হ্যাঁ (Yes) .... 1<br>না (No) 2	

No.	Questions and Filters	Coding Categories	Skip
B05	আপনারা যে ঘরে থাকেন সে ঘরের চালের/ছাদের প্রধান নির্মান-সামগ্রী কি?<br><br>*[দেখে লিখুন, যদি থাকার জন্য একাধিক ঘর থাকে তবে সবচেয়ে বড় ঘরের নির্মান-সামগ্রী লিখুন]*<br><br>Main Material of the Roof.	প্রাকৃতিক ছাদঃ Natural Roof<br>ছাদ নেই No roof .... 11<br>খড়/ছন/তালপাতা Bamboo/Thatch/Palm leaf .... 12<br>কাঁচা ছাদঃ Rudimentary Roof<br>বাঁশ Bamboo .... 21<br>কাঠের তক্তা Wood Planks .... 22<br>কার্ডবোর্ড Cardbord .... 23<br>উন্নত ছাদঃ Finished roof (Pukka)<br>টিন Tin .... 31<br>সিমেন্ট Cement/Concrete .... 32<br>টালি Tiles .... 33<br>অন্যান্য Others ________ 97<br>(নির্দিষ্ট করুন)	
B06	আপনারা যে ঘরে থাকেন সে ঘরের মেঝের প্রধান নির্মাণ-সামগ্রী কি?<br>*[দেখে লিখুন, যদি থাকার জন্য একাধিক ঘর থাকে তবে সবচেয়ে বড় ঘরের নির্মান-সামগ্রী লিখুন]*<br>Main material of the Floor.	কাঁচা মেঝে Natural floor<br>মাটি/বালু Earth/sand .... 11<br>প্রাথমিক পর্যায়ের মেঝেঃ Rudimentary floor<br>কাঠের তক্তা Wood Planks .... 21<br>তাল গাছ/ বাঁশ Palm/Bamboo .... 22<br>উন্নত মেঝে Finished floor<br>নকশা করা কাঠের পাটাতন/ পালিশকৃত কাঠ Parquet/Polished Wood .... 31<br>সিমেন্ট/ইট বালি জমানো Cement/Concrete .... 32<br>সিরামিক টাইলস/মোজাইক Ceramic Tiles/Mosaic .... 33<br>অন্যান্য Others ________ 97<br>(নির্দিষ্ট করুন)	
B07	আপনারা যে ঘরে থাকেন সে ঘরের দেয়ালের প্রধান নির্মান-সামগ্রী কি?<br>*[দেখে লিখুন, যদি থাকার জন্য একাধিক ঘর থাকে তবে সবচেয়ে বড় ঘরের নির্মান-সামগ্রী লিখুন]*<br>Main material of the Walls	প্রাকৃতিক দেয়ালঃ Natural Walls<br>দেয়াল নাই No walls .... 11<br>পাটকাঠি/বেত/তাল গাছ/গাছের গুড়ি Cane/Palm/Trunks .. 12<br>মাটি Mud .... 13<br>প্রাথমিক পর্যায়ের দেয়ালঃ Rudimentary Walls<br>মাটিসহ বাঁশ Bamboo with Mud .... 21<br>মাটিসহ পাথর Stone with Mud .... 22<br>c-vB-উড Plywood .... 23<br>কার্ডবোর্ড Cardbord .... 24<br>উন্নত দেয়ালঃ Finished Walls<br>টিন Tin .... 31<br>চুন সুড়কি দিয়ে তৈরী পাথরের দেয়াল (Stone with lime). 32<br>ইট (c-v÷vi ছাড়া) Bricks (Without plaster) .... 33<br>ইট / সিমেন্ট (c-v÷vi সহ) Bricks / Cement (With plaster) .... 34<br>উন্নতমানের কাঠের তক্তা Polished Wood Planks .... 35<br>অন্যান্য Others ________ 97<br>(নির্দিষ্ট করুন)	
B08	আপনার ঘরে/খানায় আপনাদের নিজেদের কতগুলো জীবজন্তু আছে?<br>*[যদি জানা না থাকে তাহলে '99', যদি কিছুই না থাকে তাহলে "00" বসান। যদি সংখ্যা 90 বা তার বেশী হলেও "90" লিখুন]*<br>*[কোন পশু/পাখির খামার বা বিক্রি/ব্যবসার জন্য থাকলে সেই পশু/পাখির সংখ্যা এখানে লিখবেন না]*<br>How many of the following animals are owned by your household?	গরু (Cow) .... |___|___| মহিষ (Buffalo) .... |___|___| ছাগল (Goats) .... |___|___| ভেড়া (Sheeps) .... |___|___| মুরগী (Chickens) .... |___|___| হাঁস (Ducks) .... |___|___|	

No.	Questions and Filters	Coding Categories	Skip
B09	আপনাদের কোন নিকট/কাছের আত্মীয় কি বিদেশে থাকেন/কাজ করেন? Do you have any relative living or working outside of Bangladesh?	হ্যাঁ (Yes) .... 1<br>না (No) 2	 → B11
B10	তিনি বা তারা কি আপনাকে বা আপনাদেরকে টাকা পাঠান?<br>Do they send you money from abroad?	হ্যাঁ (Yes) .... 1<br>না (No) 2	
B11	আপনার ঘরের/খানার কেউ কি এই সংস্থা/প্রতিষ্ঠানের সদস্য?<br>Does anybody from this household belong to any of the following organizations?	YES NO<br>পিপলস ইন্সটিটিউশন (People's Institution) .... 1 .... 2<br>গ্রামীন ব্যাংক (Grameen Bank) .... 1 .... 2<br>ব্রাক (BRAC) .... 1 .... 2<br>বি আর ডি বি (BRDB) .... 1 .... 2<br>প্রশিকা (Proshikha) .... 1 .... 2<br>আশা (ASHA) .... 1 .... 2<br>টিএমএসএস (TMSS) .... 1 .... 2<br>অন্য কোন সংস্থা (Any other organization) .... 1 .... 2<br>নির্দিষ্ট করুন (Specify)____________ |__|__| নির্দিষ্ট করুন (Specify) ____________ |__|__|	
B12	আপনার পরিবার (খানা) কি বসতবাড়ীর জমির মালিক? হ্যাঁ হলে কতটুকু?<br>*[না হলে 00 লিখুন, জানিনা হলে 99 লিখুন]*<br>Does your family own household land? If yes how much? [If no write 00, if don't know write 99]	|____|____| একর |____|____| শতক<br>Acre Decimal	
B13	আপনার পরিবারের (খানার) কোন চাষযোগ্য জমি আছে কি? হ্যাঁ হলে কতটুকু?<br>*[না হলে 00 লিখুন, জানিনা হলে 99 লিখুন]*<br>Does your family posses any cultivable land?, if yes how much? (If no then write 00, if doesn't know then write 99)	|____|____| একর |____|____| শতক<br>Acre Decimal	
B14	আপনি কোন ধর্মের অনুসারী?<br>What is your religion?	মুসলিম (Muslim) .... 1<br>হিন্দু (Hinduism) .... 2<br>বৌদ্ধ (Buddhism) .... 3<br>খৃষ্টান (Christianity) .... 4<br>অন্যান্য (Others) ____________ .7<br>(নির্দিষ্ট Ki"b)	
B15	আপনি কি কোন আদিবাসী গোত্র/নৃ-গোষ্ঠী/উপজাতী /সম্প্রদায়ের অন্তর্ভূক্ত?<br>Are you belonging to any ethnicity?	হ্যাঁ (Yes) .... 1<br>না (No) 2	 → C01
B16	আপনি কোন নৃ-গোষ্ঠী/সম্প্রদায়ের (উপজাতী) অন্তর্ভূক্ত?<br>What is your ethnic group?	হাজং (Hajong) .... 1<br>গারো (Garo) .... 2<br>খাসিয়া (Khasia) .... 3<br>মগ (Mog) .... 4<br>মুরং (Murong) .... 5<br>হদি (Hudi) .... 6<br>অন্যান্য (Others) ____________ .... 7<br>(নির্দিষ্ট কর_ন)	

Section C: Personal Information

১'লা জানুয়ারী ২০১৩ সাল বা তার পরে গর্ভ ফলাফল হয়েছে এমন মহিলাকে এই প্রশ্নগুলো করতে হবে।

All questions are to be addressed to women with a delivery outcome since January 1, 2013

No.	Questions And Filters	Coding Categories	Skip
C01	আপনি কোন সালের কোন মাসে জন্ম গ্রহন করেছিলেন?<br>In what month and year were you born?<br>মাস জানা না *না থাকলে "99" লিখুন*<br>(If don't know month, write '99')	মাস (Month)... |_____|_____| সাল (Year)........................... |_____|_____||_____|_____|	
C02	বর্তমানে আপনার বয়স কত?<br>How old were you at your last birthday?	বয়স (পূর্ন বছরে) Age in completed years...... |_____|_____|	
C03	আপনি কি বর্তমানে বিবাহিতা, তালাকপ্রাপ্তা, আলাদা থাকেন না বিধবা?<br>Are you now married, divorced, separated or widowed?	বিবাহিতা (married) .... 1<br>তালাকপ্রাপ্তা (divorced)... 2<br>আলাদা থাকেন (separated) .... 3<br>বিধবা (widow)... 4	 → C05<br>→ C05<br>→ C05
C04	আপনার স্বামী সর্বোচ্চ কোন ক্লাশ পাশ করেছেন (কোন ক্লাশ সম্পূর্ণ করেছেন)?<br>What is the highest grade/class or number of years of studies you have completed at that schooling?	সর্বোচ্চ কোন ক্লাস Highest Class/Grade |____|____| *(কোন ক্লাস/শ্রেণী সম্পুর্ন করে না থাকলে "00" লিখুন)*<br>*[Write '00' if no class completed]*	
C05	আপনি কি কখনও স্কুলে, মাদ্রাসায় বা উপানুষ্ঠানিক শিক্ষা স্কুলে (বয়স্ক শিক্ষা কেন্দ্র বা অন্যকোথাও) লেখাপড়া করেছেন?<br>Did you ever study in school, madrasa or non formal school?	হ্যাঁ (Yes) .... 1<br>না (No) 2	 → C07
C06	আপনি সর্বোচ্চ কোন ক্লাস/শ্রেণী পর্যন্ত লেখাপড়া করেছেন?<br>What is the highest grade/class or number of years of studies you have completed at that schooling?	সর্বোচ্চ কোন ক্লাস Highest Class/Grade |____|____| *(কোন ক্লাস/শ্রেণী সম্পুর্ন করে না থাকলে "00" লিখুন)*<br>*[Write '00' if no class completed]*	
C07	ঘরের কাজের পাশাপাশি আপনি এমন কোন কাজ কি করেন যা থেকে আপনার আয় হয়? যেমন, কেউ জিনিসপত্র বিক্রী করেন, কেউ নিজের ছোট ব্যবসায় বা পারিবারিক খামারে বা ব্যবসায় কাজ করেন, কেউ Mi"-ছাগল বর্গা নেন ইত্যাদি।<br>As you know, some women take up jobs for which they are paid in cash or kind. Others sell things, have a small business or work on the family farm or in the family business. Are you doing any of this things or any work?	হ্যাঁ Yes .... 1<br>না No 2	
	এবার আমি, আপনি **১ লা জানুয়ারী ২০১৩** সাল বা তার পরে যতবার গর্ভবতী হয়েছেন, গর্ভপাত ব্যতিত (২৮ সপ্তাহ পূর্ন হয়নি), তার প্রত্যেকটির ব্যাপারে আলোচনা করতে চাই। সেই গর্ভের ফলাফল জীবিত জন্ম বা মৃত জন্ম যাই হোক না কেন, সবগুলো সম্পর্কেই আলোচনা করতে চাই। আপনার শেষ গর্ভাবস্থা দিয়েই আলোচনা শুর" করতে চাই।<br>Now I would like to discuss with you about all of your pregnancies since 01-January-2013 except abortion (before 28 weeks), irrespective of if the child was born alive or still birth. I would like to start with your last pregnancy.		
C08	**শেষ গর্ভের ফলাফলের তারিখ** (Date of last pregnancy outcome)	|___|___|-|___|___|-|___|___|	
C09	এই গর্ভাবস্থার ফলাফল কি ছিল? জীবিত শিশু নাকি মৃত শিশু? Status of pregnancy Outcome	জীবিত জন্ম Live birth .... 1<br>মৃত জন্ম. Still Birth 2	→ C11
C10	জন্মের পর বাচ্চাটি কি কেঁদেছিল বা নড়াচড়া করেছিল বা শ্বাসপ্রশ্বাস নিয়েছিল?<br>Cry/move/breathe after birth	হ্যাঁ Yes .... 1<br>না No 2	 → C13
C11	শিশুর বর্তমান অবস্থা<br>Status of the child (alive or dead)	জীবিত এবং সাথে থাকে (Alive & live with mother)..............1<br>জীবিত কিন্তু অন্যত্র থাকে (Alive but live elsewhere)............2<br>মৃত (Not alive)...3	→ C13<br>→ C13
C12	যদি মৃত হয়: মৃত্যুর সময় তার বয়স কত ছিল?<br>*[দুই মাস (60 দিন) এর কম হলে দিনে লিখুন]*<br>If the child died, write down childs age at death	দিন Days.. 1 |___|___| মাস Months .... 2 |___|___|	

No.	Questions And Filters	Coding Categories	Skip
C13	**শেষ গর্ভের পূর্ববর্তী গর্ভাবস্থার ফলাফলের তারিখ** (Date of pregnancy outcome before the last birth)	ফলাফল আছে …. 1 |___|___|-|___|___|-|___|___| আর কোন গর্ভের ইতিহাস নাই …………………………. 2	→ D01
C14	এই গর্ভাবস্থার ফলাফল কি ছিল? জীবিত শিশু নাকি মৃত শিশু? Status of pregnancy Outcome	জীবিত জন্ম Live birth ……………………………………… 1<br>মৃত জন্ম. Still Birth ……………………………………… 2	→ C16
C15	জন্মের পর বাচ্চাটি কি কেঁদেছিল বা নড়াচড়া করেছিল বা শ্বাসপ্রশ্বাস নিয়েছিল?<br>Cry/move/breathe after birth	হ্যাঁ Yes ……………………………………………………… 1<br>না No ……………………………………………………… 2	→ D01
C16	(নাম) কি এখনও জীবিত?<br>Status of the child (alive or dead)	জীবিত এবং সাথে থাকে (Alive & live with mother)…………..1<br>জীবিত কিন্তু অন্যত্র থাকে (Alive but live elsewhere)…………. 2<br>মৃত (Not alive)………………………………………………….. 3	→ D01<br>→ D01
C17	যদি মৃত হয়: মৃত্যুর সময় তার বয়স কত ছিল?<br>*[দুই মাস (60 দিন) এর কম হলে দিনে লিখুন]*<br>If the child died, write down child's age at death	দিন Days…………………………………………1 |___|___| মাস Months ………………………………………2 |___|___|	

Section D: People's Institution Group

This section contains some information about People's Institution group

আপনি হয়ত জানেন যে কখনও কখনও এলাকা ভিত্তিক কিছু কমিটি/দল থাকে। আমি এখন এসব বিষয়ে কিছু প্রশ্ন করব।

You may know that there are local committees in many communities. Now I will discuss about such commnitte / group in your area.

No.	Questions And Filters	Coding Categories	Skip
D01	আপনার এলাকায় এমন কোন কমিটি বা দল (গ্রুপ) সম্পর্কে আপনি জানেন কি যারা মা ও শিশুর স্বাস্থ্যের উন্নতির জন্য কাজ করে?<br>Do you know about any committees, or network or group in your community that works towards improving maternal and child health?	হ্যাঁ Yes ………………………………………………………… 1<br>না No ………………………………………………………… 2	→ D12
D02	আপনি বা আপনার পরিবারের কোন সদস্য কি এই ধরনের কমিটি বা দল (গ্রুপ) এর সদস্য?<br>Are you and/or any of your family member is a member of any such group?	হ্যাঁ Yes ………………………………………………………… 1<br>না No ………………………………………………………… 2	→ D04
D03	আপনি/আপনার পরিবারের সদস্য কতদিন আগে ঐ কমিটি বা দলের (গ্রুপের) সদস্য হয়েছেন? [বছরে বললে মাসে পরিবর্তিত করুন। ১ মাসের কম হলে ০০ লিখুন]<br>How long ago did you or someone in your family become a member of this group?<br>Interviewer: If respondent answer in years convert it into months. If less than one month write '00' in the box	মাস MONTH……………………………………………… |___|___| মনে নাই Don't Remember ……………………………………… 99	
D04	এই কমিটি বা গ্রুপ কোন কোন বিষয় নিয়ে কাজ করে?<br>What issues does the group deal with?	মায়ের স্বাস্থ্য সেবা Mother's health ……………………………A<br>শিশুর স্বাস্থ্য সেবা Baby's health………………………………B<br>পরিবার পরিকল্পনা Family planning……………………………C<br>শিক্ষা Education ……………………………………………………D<br>অসুস্থ মা ও শিশুর পরিবহনের ব্যবস্থা করে<br>Arrange transportation for sick mother & babies …………… E<br>অসুস্থ মা ও শিশুর চিকিৎসার জন্য অর্থের ব্যবস্থা করে<br>Financing sick mother & babies ……………………………F<br>অন্যান্য Others________________________.....X<br>নির্দিষ্ট করুন<br>জানে না Don't know …………………………………………… Z	
D05	এই কমিটি /দলের কাছে আপনি কি মায়ের অথবা শিশুর স্বাস্থ্য সম্পর্কিতকোন প্রকার সমস্যার কারনে সাহায্যের জন্য গিয়েছিলেন?<br>Did you ever go for any sort of help from this group for maternal or child health related problems?	হ্যাঁ Yes ……………………………………………………………1<br>না No ……………………………………………………………2	

No.	Questions And Filters	Coding Categories	Skip
D06	এই কমিটি/দলের কাছ থেকে আপনি কি কখনও মায়ের অথবা শিশুর স্বাস্থ্য সম্পর্কিত কোন প্রকার সমস্যার কারনে কোন ধরনের সাহায্য পেয়েছিলেন?<br>Have you ever received any sort of help from this group for maternal or child health related problems?	হ্যাঁ Yes ....1<br>না No 2	 → D12
D07	এই কমিটি/দলের কাছ থেকে মায়ের অথবা শিশুর স্বাস্থ্য সম্পর্কিতকোন প্রকার সমস্যার কারনে কি ধরনের সাহায্য পেয়েছিলেন?<br>What kind of support did you receive from this group for maternal or child health related problems?	পরিবহনের ব্যবস্থা করেছিল Arranged transportation ....A<br>টাকা পয়সার ব্যবস্থা করেছিল Aranged financial support ....B<br>স্বাস্থ্যকর্মীকে জানানো Informed health worker ....C<br>স্বাস্থ্যকর্মীর পরিদর্শন নিশ্চিত করা Ensured visit of health worker ....D<br>স্বাস্থ্যকেন্দ্র থেকে সেবা পাওয়ার ক্ষেত্রে সহায়তা করেছিল Ensured service from/linkage with health facility ....E<br>অন্যান্য Other________ X	
D08	***[D 07 এ A বৃত্তায়িত হলে]*** এই কমিটি/গ্রুপের কাছ থেকে কি ধরনের পরিবহন সুবিধা পেয়েছিলেন?<br>**If D07 A is circled**, ask, what kind of support for transportation did you receive from the group?	যানবাহন খুঁজতে সাহায্য করেছে Helped in finding a vehicle......1<br>যানবাহন দিয়েছে Provided with a vehicle ....2<br>প্রযোজ্য নয় Not applicable 3	
D09	***[D 07 এ B বৃত্তায়িত হলে]*** তাদের তহবিল থেকে সরাসরি আপনাকে টাকা দিয়েছিল না-কি এলাকা থেকে টাকা তুলে দিয়েছিল?<br>**If D07 B is circled,** ask did they give you money directly from their fund or arranged money from the community?	তহবিল থেকে দিয়েছিল Gave money from their fund ....1<br>এলাকা থেকে টাকা তুলে দিয়েছিল Arranged from community ... 2<br>উভয়েই Both ....3<br>প্রযোজ্য নয় Not applicable ....3<br>জানি না Don't know........9	
D10	***[D 07 এ E বৃত্তায়িত হলে*** এই কমিটি/গ্রুপের কাছ থেকে **স্বাস্থ্যকেন্দ্র হতে সেবা পাওয়ার ক্ষেত্রে** কি ধরনের **সহায়তা** পেয়েছিলেন?<br>**If D07 E is circled**, ask, what kind of support for service/linkage did you receive from the group?	________________|___|___| ________________|___|___| প্রযোজ্য নয় Not applicable 3	
D11	এই কমিটি/দলের কাছ থেকে মায়ের অথবা শিশুর স্বাস্থ্য সম্পর্কিত সমস্যার কারনে যে সাহায্য পেয়েছিলেন তা কি আপনার কাছে উপকারী মনে হয়েছে?<br>Do you consider the help that you received for maternal or child health related problems as beneficial?	হ্যাঁ Yes ....1<br>না No 2	

তথ্যসংগ্রহকরীর জন্য নির্দেশিকা Instruction for interviewer**: CHECK Questions**

D12	এই মহিলার ১ লা জানুয়ারী ২০১৩ সাল বা তার পরে কোন গর্ভ ফলাফল আছে কি (**Question C08 and C13)?**<br>Is there any pregnancy outcome for this woman since 01-January-2013 (**Question C08 and C13)**?	হ্যাঁ Yes ....1<br>না No 2	 → D14
D13	আপনাকে কি এই মহিলার নিকট হতে maternal and immediate newborn care (Module II) এর জন্য তথ্য সংগ্রহ করতে বলা হয়েছে?<br>Were you asked to collect information on maternal and immediate newborn care (Module II)?	হ্যাঁ Yes ....1<br>না No 2	**→ Module II**
D15	উত্তর দাতাকে ধন্যবাদ জানিয়ে সাক্ষাৎকার শেষ করুন।<br>Thanks the mother for providing time and end the interview.		

IFC Framework Evaluation Baseline Survey 2014

Module II

Maternal and immediate newborn care module

ALL QUESTIONS ARE TO BE ADDRESSED TO WOMEN WITH A PREGNANCY OUTCOME SINCE 1’st JANUARY 2013

	Name	Code	
**CLUSTER/UNION**		☐☐	সাক্ষাৎকার শুরু হবার সময়:
**UPAZILA**		☐☐	|___|___|:|___|___|
**VILLAGE**		☐☐☐☐	
**HOUSEHOLD ID**		☐☐☐☐	সাক্ষাৎকার শেষ করার সময়
**WOMEN ID**		☐☐☐	|___|___|:|___|___|

Section E: Maternal Care

No.	Questions And Filters	Coding Categories	Skip
E00	গর্ভকালীন সময়ে একজন মহিলার মানসম্মত মাতৃ স্বাস্থ্যসেবা পাওয়ার অধিকার রয়েছে, এ ব্যাপারে আপনি জানেন কি?<br>Are you aware that a pregnant women has the right to access quality MNH services?	হ্যাঁ Yes ..... 1<br>না No ..... 2<br>জানি না/ মনে নাই Don't know/Can't remember 9	→Supp Mod 2 (E 00a, E00b, E00c)
E01	গর্ভকালীন সময়ে একজন মহিলা অসুস্থ না হলেও মেডিকেল চেকআপ (ANC) করার দরকার আছে কি?<br>Do you know about ANC (Ante natal care), a medical check-up for a woman during pregnancy , even when she is not sick?	হ্যাঁ Yes ..... 1<br>না No ..... 2<br>জানি না/ মনে নাই Don't know/Can't remember 9	2→ E03<br>9→ E03
E02	গর্ভকালীন সময়ে একজন মহিলার কমপক্ষে কত বার মেডিকেল চেকআপ (ANC) করা দরকার?<br><br>Can you tell me at least how many times a pregnant woman should receive such kind of medical check-up (ANC) during pregnancy?	বার (গর্ভকালীন সময়ে) .... |____|____| Times (during pregnancy)<br><br>জানি না/ মনে নাই Don't know/Can't remember 9	
E03	শেষ গর্ভের সময়, গর্ভকালীন মেডিকেল চেক-আপের জন্য আপনি কি কাউকে দেখিয়েছিলেন?<br>Did you see (consult) anyone for antenatal care during your most recent pregnancy?	হ্যাঁ Yes ..... 1<br>না No ..... 2<br>জানি না/ মনে নাই Don't know/Can't remember 9	2→ E11<br><br>9→ E11
E04	শেষ গর্ভের সময়, গর্ভকালীন মেডিকেল চেক-আপের জন্য আপনি কাকে দেখিয়েছিলেন?<br><br>*[মহিলাকে জিজ্ঞেস করুন]* আরও কিছু? *[মহিলার নিজে থেকে দেয়া সবগুলো উত্তরই বৃত্তায়িত করুন]* । *উত্তরগুলো পড়ে শুনাবেন না। একাধিক উত্তর হতে পারে।]*<br><br>Whom you see for antenatal care during you most recent pregnancy? [Do not readout the answers. Ask anyone else? Record all the answers]	**দক্ষ/প্রশিক্ষণ প্রাপ্ত স্বাস্থ্য কর্মী (Medically trained)**<br>পাশ করা ডাক্তার (MBBS doctor)..... A<br>নার্স/ধাত্রী (Nurse/midwife) ..... B<br>প্যারামেডিক/মেডিকেল এসিসটেন্ট/সাকমো (Paramedic/MA/SACMO)..... C<br>পরিবার কল্যাণ পরিদর্শক (FWV)..... D<br>সি,এস,বি,এ (CSBA) ..... E<br>**অন্যান্য স্বাস্থ্য কর্মী (Other health worker)**<br>স্বাস্থ্য সহকারী/ পরিবার কল্যান সহকারী (HA /FWA) . F<br>পুষ্টি কর্মী (CNP)..... G<br>সুসমা কার্যক্রমের স্বেচ্ছাসেবী (Volunteer of SUSOMA prj)H<br>অন্যান্য কমিউনিটি স্বাস্থ্য কর্মী - এনজিও কর্মী, স্বেচ্ছাসেবী (Other CHWs, NGO worker, volunteer)..... I<br>**অন্যান্য (Other)**<br>প্রশিক্ষণ প্রাপ্ত টিবিএ (প্রশিক্ষণ প্রাপ্ত ধন্নী, চাউনী, দাই) (TTBA) ..... J<br>প্রশিক্ষণহীন টিবিএ (ধন্নী, চাউনী, দাই) TBA(Dai/Dhorni/Chauni) ..... K<br>হোমিওপ্যাথ/হোমিওপ্যাথ ঔষধের দোকান (Homeopath/Homeopath drug store) ..... L<br>আয়ুর্বেদিক চিকিৎসক / আয়ুর্বেদিক ঔষধের দোকান /হেকিম/কবিরাজ (Ayurved/ Ayurvedic drug store /Hekim/Kabiraj) ..... M<br>গ্রাম ডাক্তার (Village doctor) ..... N<br>এলোপ্যাথী ঔষধের দোকান (Allopath drug store) ... O<br>ইমাম/ঝাড় ফুক/ওঝা (Spiritual healer) ..... P<br>পরিবারের অন্যান্য সদস্য/আত্মীয়/ প্রতিবেশী/বন্ধু Family/relative/Neighbor/friend..... Q<br>অন্যান্য Others________________... X<br>(নির্দিষ্ট করুন)<br>জানি না/মনে নাই Don't know/can't remember .... Z	

No.	Questions And Filters	Coding Categories	Skip
E05	শেষ গর্ভের সময়, গর্ভকালীন মেডিকেল চেক-আপ আপনি কোথায় নিয়েছিলেন/করিয়েছিলেন?<br><br>*[মহিলাকে জিজ্ঞেস করুন] আরও কিছু? [মহিলার নিজে থেকে দেয়া সবগুলো উত্তরই বৃত্তায়িত করুন । উত্তরগুলো পড়ে শুনাবেন না । একাধিক উত্তর হতে পারে ।]*<br><br>*[মহিলা যেখান থেকে সেবা পেয়েছেন, সেই স্বাস্থ্যকেন্দ্রের নাম লিখুন । যদি একাধিক জায়গা থেকে সেবা নিয়ে থাকেন, তাহলে সবগুলো জায়গারই নাম এবং কোড লিখুন ।]*<br><br>Code :|____| নাম Name: ______________________________<br><br>Code :|____| নাম Name: ______________________________<br><br>Code :|____| নাম Name: ______________________________<br><br>Where did you receive antenatal care for this most recent pregnancy? Do not readout the answers. Ask anywhere else? Record all the answers. Record the name of the places where the women sought care.	**<u>সরকারী স্বাস্থ্য কেন্দ্র (Govt Health center)</u>**<br>মেডিকেল কলেজ হাসপাতাল (Medical College Hospital)....A<br>জেলা/সদর হাসপাতাল (District /Sadar Hospital)............B<br>মা ও শিশু স্বাস্থ্য কেন্দ্র (MCWC)..............................C<br>উপজেলা স্বাস্থ্য কমপেপ্লক্স (UHC)............................D<br>ইউনিয়ন স্বাস্থ্য ও পরিবার কল্যাণ কেন্দ্র/সাব সেন্টার/আরডি (FWC/SC/RD)...E<br>কমিউনিটি ক্লিনিক (Community clinic)........................F<br>সেটেলাইট ক্লিনিক/ ইপিআই কেন্দ্র (Satellite clinic/EPI centre)..G<br>অন্যান্য সরকারী স্বাস্থ্য কেন্দ্র (Other Govt Health facility) ..H<br>**<u>বেসরকারী স্বাস্থ্য কেন্দ্র (Non Govt Health center)</u>**<br>এনজিও হাসপাতাল (NGO hospital) ....I<br>এনজিও স্থায়ী স্বাস্থ্য কেন্দ্র (NGO static health centre) ......J<br>এনজিও সেটেলাইট ক্লিনিক (NGO satellite clinic)...........K<br>পুষ্টি কেন্দ্র (NNP centre).......................................L<br>অন্যান্য বেসরকারী স্বাস্থ্য কেন্দ্র (Other NGO Health facility)...M<br>**<u>প্রাইভেট (Private Health sector)</u>**<br>হাসপাতাল/ ক্লিনিক (Hospital/clinic)..........................N<br>স্বাস্থ্য কেন্দ্র /ডিসপেনসারী (Health centre/Dispensary) .....O<br>এমবিবিএস ডাক্তারের চেম্বার (MBBS doctor's chamber) ...P<br>গ্রাম ডাক্তারের চেম্বার (Village doctor's chamber)...........Q<br>প্যারামেডিক/মেডিকেল এসিসটেন্ট/সাকমোর চেম্বার (Paramedic/ MA/SACMO chamber)... R<br>এলোপ্যাথী ঔষধের দোকান (Allopath drug store) ....S<br>অন্যান্য প্রাইভেট স্বাস্থ্য কেন্দ্র (Other private Health facility)T<br><br>**<u>বাড়ী (Home)</u>**<br>নিজ বাড়ী, স্বামী/শ্বশুড় বাড়ী (Own home, husband/father in laws house)...U<br>বাবার বাড়ী (My natal home) ....V<br><br>অন্যান্য (Others________________________________..X<br>(নির্দিষ্ট কর্ন)<br>জানি না/মনে নাই Don't know/can't remember ....Z	
E06	*[Question E04 দেখুন, code A থেকে E এর যে কোন একটি বা একাধিক বৃত্তায়িত থাকলে কোড/কোডগুলো এখানে লিখুন: _____ , _____, ____ । এবার কোড/কোডগুলো দেখে দেখে মহিলাকে প্রশ্ন কর্ন ঃ*<br><br>আপনি এর/এদের ভিতর যার কাছ থেকে প্রথম গর্ভকালীন মেডিকেল চেক-আপ করিয়েছিলেন/নিয়েছিলেন, তখন আপনি কত মাসের গর্ভবতী ছিলেন?<br>How many months pregnant were you when you first received antenatal care from for this pregnancy from the mentioned skilled health care provides (Code A-E of Q E04)?	মাসের গর্ভবতী Month-pregnant (during 1st check-up).....................................|______|______| <br>প্রযোজ্য নয় Not applicable .... 97<br><br>জানা নাই/মনে নাই DK/can't remember.............. 99	

No.	Questions And Filters	Coding Categories	Skip
E07	*[Question E04 দেখুন, code A থেকে E এর যে কোন একটি বা একাধিক বৃত্তায়িত থাকলে কোড/কোডগুলো এখানে লিখুন: _____ , _____, ____ । এবার কোড/কোডগুলো দেখে দেখে মহিলাকে প্রশ্ন কর‍ু‍ন ঃ]*<br>আপনি এর/এদের কাছ থেকে মোট কতবার গর্ভকালীন মেডিকেল চেক-আপ করিয়েছিলেন /নিয়েছিলেন?<br>How many times did you receive antenatal care from for this pregnancy from the mentioned skilled health care provides (Code A-E of Q E04)?	বার Number .... |____|____| প্রযোজ্য নয় Not applicable .... 97<br>জানা নাই/মনে নাই DK/can't remember 99	
E08	শেষ গর্ভের সময়, গর্ভকালীন মেডিকেল চেক-আপের সময় কখনও কি আপনার ----------- ? (প্রত্যেকটি বিষয় পড়ে শোনান)<br>ওজন নেয়া হয়েছিল?<br>রক্ত - চাপ পরীক্ষা করা হয়েছিল?<br>প্রস্রাব পরীক্ষা করা হয়েছিল?<br>রক্ত পরীক্ষা করা হয়েছিল?<br>আলট্রাসনোগ্রাম করা হয়েছিল?<br>আপনার পেটে হাত দিয়ে পরীক্ষা করা হয়েছিল?<br>As part of your antenatal care during this pregnancy, were any of the following done at least once?<br>-Were you weighed?<br>-Was your blood pressure measured?<br>-Did you give a urine sample?<br>-Did you give a blood sample?<br>-Was your ultrasonogram done?<br>-Was your abdominal examination done	হ্যাঁ Yes না No<br>ওজন Weight .... 1........... 2<br>রক্ত - চাপ Blood pressure .... 1........... 2<br>প্রস্রাব পরীক্ষা Urine test....................... 1........... 2<br>রক্ত পরীক্ষা Blood test......................... 1........... 2<br>আলট্রাসনোগ্রাম Ultra sonogram .... 1........... 2<br>পেটে হাত দিয়ে পরীক্ষা Abdominal exam .. 1........... 2	
E09	শেষ গর্ভের সময়, যখন আপনি গর্ভকালীন চেক-আপ করিয়েছিলেন তখন কখনও আপনাকে গর্ভকালীন বিপদচিহ্ন সম্পর্কে কিছু বলা হয়েছে কি?<br>During (any of) your antenatal care visits of your most recent pregnancy, were you told about the signs of pregnancy complications?	হ্যাঁ Yes .... 1<br>না No .... 2<br>জানি না/ মনে নাই Don't know/Can't remember 9	**2→ E11**<br>**9→ E11**
E10	কোন একটি গর্ভকালীন বিপদচিহ্ন দেখা দিলে আপনাকে কোথায় যেতে হবে সে ব্যাপারে আপনাকে কিছু বলা হয়েছিল কি?<br>Were you told where to go if you had any of these complications?	হ্যাঁ Yes .... 1<br>না No .... 2<br>জানি না/ মনে নাই Don't know/Can't remember 9	

No.	Questions And Filters	Coding Categories	Skip
E11	কখনও কখনও গর্ভকালীন সময় কিছু বিপদ চিহ্ন দেখা যেতে পারে এবং তখন গর্ভবতীকে তাৎক্ষনিকভাবে স্বাস্থকেন্দ্রে পাঠানো দরকার হয়।<br><br>গর্ভকালীন সময়ে কি কি বিপদচিহ্ন দেখা দিলে একজন মহিলাকে তাৎক্ষনিকভাবে হাসপাতালে পাঠাতে হয় ?<br><br>*[মহিলাকে জিজ্ঞেস করুন]* আরও কোন সমস্যা/জটিলতা? *[মহিলার নিজে থেকে দেয়া সবগুলো উত্তরই বৃত্তায়িত করুন । উত্তরগুলো পড়ে শুনাবেন না। একাধিক উত্তর হতে পারে।]*<br><br>During pregnancy, women may encounter severe problems or illness and should go or be taken immediately to a health facility.<br><br>What types of symptoms would cause you to seek care at a health facility right away?<br><br>Ask: Anything else?<br>Circle all signs mentioned, but do not prompt with any suggestions.	যোনীপথে রক্তস্রাব Vaginal Bleeding ....A<br>দ্রুত শ্বাস /শ্বাস নিতে কষ্ট হওয়া Fast/difficult breathing ....B<br>জ্বর Fever ....C<br>তলপেটে তীব্র ব্যথা Severe abdominal pain ....D<br>মাথা ব্যথা চোখে ঝাপসা দেখা<br>Headache/ Blurred Vision ....E<br>খিচুনী/ফিট Convulsions/fits ....F<br>দুর্গন্ধযুক্ত স্রাব Foul smelling vaginal discharge ....G<br>গর্ভের বাচ্চার নড়াচড়া কমে যাওয়া/বন্ধ হওয়া<br>Fetal movement reduced/absent ....H<br>যোনীপথ দিয়ে ঘোলাটে/সবুজাভ কিছু বের হওয়া Leaking brownish/greenish fluid from the vagina ....I<br>হাতে পানি আসা/আঙ্গুল ফুলে যাওয়া Edema of hand/fingers ....J<br>মুখে / পায়ে পানি আসা Edema of face/ leg.................K<br>অন্যান্য Others ________________________________....X<br>নির্দিষ্ট করুন<br>জানা নাই/মনে নাই DK/can't remember ....Z	
E12	শেষ গর্ভের সময়, আপনি আয়রণ ট্যাবলেট বা আয়রন সিরাপ পেয়েছিলেন/কিনেছিলেন কি? *[প্রয়োজনে মহিলাকে আয়রন ট্যাবলেট বা আয়রন সিরাপের বোতল দেখান]*<br>During your last pregnancy, were you given or did you buy any iron tablets/syrup? [If necessary then show the tablet or bottle of syrup and then ask]	হ্যাঁ Yes .... 1<br>না No .... 2<br>জানি না/ মনে নাই Don't know/Can't remember 9	**2→ E14**<br>**9→ E14**
E13	শেষ গর্ভের সময়, আপনি মোট কত দিন আয়রন ট্যাবলেট/সিরাপ খেয়েছিলেন?<br>সংখ্যায় বলতে না পারলে, কাছাকাছি দিনের সংখ্যা লিখুন<br><br>During the whole pregnancy, how many days did you take the tablet/ syrup?<br>If the answer is not numeric, probe for approximate number of days.	দিন Days .... |___|___|___|	
E14	শেষ গর্ভের সময়, সুসমা কার্যক্রমের কোন মাদারস্ গ্রুপ এর কোন মিটিং এ অংশগ্রহণ করেছিলেন?<br><br>During yourlast pregnancy, did you attend any meeting of mothers group of SUSOMA project?	হ্যাঁ Yes .... 1<br>না No .... 2<br>জানি না/ মনে নাই Don't know/Can't remember 9	
E14a	শেষ গর্ভের সময় বাচ্চা হওয়া এবং জরুরী অবস্থা বিষয়ক পরিকল্পনার জন্য কোন স্বাস্থ্যকর্মীর সাথে আলাপ/পরামর্শ করেছিলেন কি?<br>Did you discuss a plan for birth and emergencies with a health care worker?	হ্যাঁ Yes .... 1<br>না No .... 2<br>জানি না/ মনে নাই Don't know/Can't remember 9	**2→ E14c**<br>**9→ E14c**

No.	Questions And Filters	Coding Categories	Skip
E14b	কার সাথে আলাপ/পরামর্শ করেছিলেন?<br><br>With whom?	**দক্ষ/প্রশিক্ষণ প্রাপ্ত স্বাস্থ্য কর্মী (Medically trained)**<br>পাশ করা ডাক্তার (MBBS doctor)............................A<br>নার্স/ধাত্রী (Nurse/midwife) ....B<br>প্যারামেডিক/মেডিকেল এসিসটেন্ট/সাকমো<br>(Paramedic/MA/SACMO)..................................C<br>পরিবার কল্যাণ পরিদর্শক (FWV)............................D<br>সি,এস,বি,এ (CSBA) ....E<br>**অন্যান্য স্বাস্থ্য কর্মী (Other health worker)**<br>স্বাস্থ্য সহকারী/ পরিবার কল্যান সহকারী (HA /FWA) ......F<br>পুষ্টি কর্মী (CNP)..G<br>সুসমা কার্যক্রমের স্বেচ্ছাসেবী (Volunteer of SUSOMA prj) ...H<br>অন্যান্য কমিউনিটি স্বাস্থ্য কর্মী - এনজিও কর্মী, স্বেচ্ছাসেবী<br>(Other CHWs, NGO worker, volunteer)..................I<br>**অন্যান্য (Other)**<br>প্রশিক্ষণ প্রাপ্ত টিবিএ (প্রশিক্ষণ প্রাপ্ত ধন্নী,<br>চাউনী, দাই) (TTBA) ....J<br>প্রশিক্ষণহীন টিবিএ (ধন্নী, চাউনী, দাই)<br>TBA(Dai/Dhorni/Chauni) ....K<br>হোমিওপ্যাথ/হোমিওপ্যাথ ঔষধের দোকান<br>(Homeopath/Homeopath drug store) ....L<br>আয়ুর্বেদিক চিকিৎসক / আয়ুর্বেদিক ঔষধের দোকান<br>/হেকিম/কবিরাজ (Ayurved/ Ayurvedic drug store<br>/Hekim/Kabiraj) ....M<br>গ্রাম ডাক্তার (Village doctor) ....N<br>এলোপ্যাথী ঔষধের দোকান (Allopath drug store) ....O<br>ইমাম/ঝাড় ফুক/ওঝা (Spiritual healer) ....P<br>পরিবারের অন্যান্য সদস্য/আত্মীয়/ প্রতিবেশী/বন্ধু<br>Family/relative/Neighbor/friend............................Q<br>অন্যান্য Others________________________........X<br>(নির্দিষ্ট করুন)<br>কেউ অসেনি None came..Y<br>জানি না/মনে নাই Don't know/can't remember ....Z	
E14c	শেষ গর্ভের সময় বাচ্চা হওয়া এবং জরুরী অবস্থা বিষয়ক পরিকল্পনার জন্য আপনার স্বামীর সাথে আলাপ/পরামর্শ করেছিলেন কি?<br>Did you discuss a plan for birth and emergencies with your husband?	হ্যাঁ Yes .... 1<br>না No .... 2<br>জানি না/ মনে নাই Don't know/Can't remember 9	**→Supp Mod 2 (E 14c2, E 14c3, E14d)**
E15	ডেলিভারীর সময় ব্যবহারের জন্য শেষবার গর্ভবতী থাকা অবস্থাতেই ডেলিভারী কিট ক্রয় বা সংগ্রহ করে রেখেছিলেন কি?<br>Did you buy / collect delivery kit for using during delivery?	হ্যাঁ Yes .... 1<br>না No .... 2<br>জানি না/ মনে নাই Don't know/Can't remember 9	
E16	ডেলিভারীর সময় যদি কোন অসুবিধা হয়, তাহলে যেন তাড়াতাড়ি হাসপাতাল বা স্বাস্থ্যকেন্দ্রে যাওয়া যায়, সে জন্য আগে থেকেই কি যানবাহনের ব্যবস্থা করে রেখেছিলেন?<br>Did you arrange emergency transport before delivery in case complication during delivery?	হ্যাঁ Yes .... 1<br>না No .... 2<br>জানি না/ মনে নাই Don't know/Can't remember 9	
E17	ডেলিভারীর সময়টায় টাকা পয়সা লাগতে পারে, তাই আগে থেকেই টাকা পয়সা জমা করে রেখেছিলেন কি?<br>Did you save money for emergency need during delivery?	হ্যাঁ Yes .... 1<br>না No .... 2<br>জানি না/ মনে নাই Don't know/Can't remember 9	
E17a	ডেলিভারীর সময় যদি রক্তের প্রয়োজন হয়, সে জন্য আগে থেকেই কি একজন রক্তদাতা নির্বাচন করে রেখেছিলেন?<br>Did you identify a potential blood donor before delivery ?	হ্যাঁ Yes .... 1<br>না No .... 2<br>জানি না/ মনে নাই Don't know/Can't remember 9	**2→ E18<br>9→ E18**

No.	Questions And Filters	Coding Categories	Skip
E17b	কাকে ঠিক করে রেখেছিলেন?<br><br>Whom did you identify?	একই খানার লোকজন Household Member................ 1<br>রক্ত সম্পর্কের আত্মীয় Blood relative .... 2<br>প্রতিবেশী Neighbour.. 3<br>অপরিচিত ব্যক্তি Unknown person............................ 4<br>অন্যান্য _________________________ .... 7<br>(নির্দিষ্ট কর—ন)<br>জানি না/ মনে নাই Don’t know/Can’t remember 9	
E18	ডেলিভারীর পর পর শিশুটিকে মুড়ানো মোছানোর জন্য কমপক্ষে দুই টুকরা কাপড় কি জোগাড় করে রেখেছিলেন?<br>Did you arrange two pieces of cloth for drying and wrapping the baby?	হ্যাঁ Yes .... 1<br>না No .... 2<br>জানি না/ মনে নাই Don’t know/Can’t remember 9	→ Supp Mod 2 (E 18b)
E19	শেষ যখন আপনি গর্ভবতী ছিলেন, তখন (গর্ভবতী থাকাকালীন সময়ে) আপনার এই ডেলিভারীটি কোথায় হবে, সেটি কি ঠিক করে রেখেছিলেন?<br>*[উত্তর 'হ্যাঁ' হলে, জানতে চান,]* কোথায় ডেলিভারী/শিশুটি হবে বলে ঠিক করে রেখেছিলেন?<br>Did you select the place during pregnancy where your child would be deliverd? If yes, ask which place did you select?	বাসায় At Home .... 1<br>স্বাস্থ্যকেন্দ্রে Health Center .... 2<br>আগে থেকে ঠিক করে রাখি নাই Didn't have any plan . 9	
E20	শেষ যখন আপনি গর্ভবতী ছিলেন, তখন (গর্ভবতী থাকাকালীন সময়ে) আপনার ডেলিভারীতে সাহায্য করার জন্য বা ডেলিভারীটি করানোর জন্য বা ডেলিভারীর সময় উপস্থিত থাকার জন্য পছন্দের কাউকে আপনারা ঠিক করে রেখেছিলেন কি?<br>During your most recent pregnancy, did you or your family select someone or a companion of choice to be present for birth/ delivery?	হ্যাঁ Yes .... 1<br>না No .... 2<br>জানি না/ মনে নাই Don’t know/Can’t remember 9	2→ E21<br>9→ E21

No.	Questions And Filters	Coding Categories	Skip
E20a	কাকে ঠিক করে রেখেছিলেন?<br><br>Whom did you identify?	**দক্ষ/প্রশিক্ষণ প্রাপ্ত স্বাস্থ্য কর্মী (Medically trained)**<br>পাশ করা ডাক্তার (MBBS doctor)............................A<br>নার্স/ধাত্রী (Nurse/midwife) ....B<br>প্যারামেডিক/মেডিকেল এসিসটেন্ট/সাকমো<br>(Paramedic/MA/SACMO)..................................C<br>পরিবার কল্যাণ পরিদর্শক (FWV).............................D<br>সি,এস,বি,এ (CSBA) ....E<br>**অন্যান্য স্বাস্থ্য কর্মী (Other health worker)**<br>স্বাস্থ্য সহকারী/ পরিবার কল্যান সহকারী (HA /FWA) ......F<br>পুষ্টি কর্মী (CNP)..G<br>সুসমা কার্যক্রমের স্বেচ্ছাসেবী (Volunteer of SUSOMA prj) ...H<br>অন্যান্য কমিউনিটি স্বাস্থ্য কর্মী - এনজিও কর্মী, স্বেচ্ছাসেবী<br>(Other CHWs, NGO worker, volunteer)..................I<br>**অন্যান্য (Other)**<br>প্রশিক্ষণ প্রাপ্ত টিবিএ (প্রশিক্ষণ প্রাপ্ত ধন্নী,<br>চাউনী, দাই) (TTBA) ....J<br>প্রশিক্ষণহীন টিবিএ (ধন্নী, চাউনী, দাই)<br>TBA(Dai/Dhorni/Chauni) ....K<br>হোমিওপ্যাথ/হোমিওপ্যাথ ঔষধের দোকান<br>(Homeopath/Homeopath drug store) ....L<br>আয়ুর্বেদিক চিকিৎসক / আয়ুর্বেদিক ঔষধের দোকান<br>/হেকিম/কবিরাজ (Ayurved/ Ayurvedic drug store<br>/Hekim/Kabiraj) ....M<br>গ্রাম ডাক্তার (Village doctor) ....N<br>এলোপ্যাথী ঔষধের দোকান (Allopath drug store) ....O<br>ইমাম/ঝাড় ফুক/ওঝা (Spiritual healer) ....P<br>পরিবারের অন্যান্য সদস্য/আত্মীয়/ প্রতিবেশী/বন্ধু<br>Family/relative/Neighbor/friend............................Q<br>অন্যান্য Others______________________........X<br>(নির্দিষ্ট করুন)<br>জানি না/মনে নাই Don't know/can't remember ....Z	

No.	Questions And Filters	Coding Categories	Skip
E21	শেষ গর্ভের সময়, আপনাকে দেখতে বা আপনার স্বাস্থ্যের ব্যাপারে উপদেশ দিতে আপনার বাসায় কে এসেছিলেন?<br>*[মহিলাকে জিজ্ঞেস করুন]* : আরও কেউ?<br><br>*মহিলার নিজে থেকে দেয়া সবগুলো উত্তরই বৃত্তায়িত করুন । উত্তরগুলো পড়ে শুনাবেন না । একাধিক উত্তর হতে পারে ।*<br><br>Who had visited you to discuss issues regarding your health during your most recent pregnancy? [Do not read out the answers. Ask: Anything else? Circle all the answers]	**দক্ষ/প্রশিক্ষণ প্রাপ্ত স্বাস্থ্য কর্মী (Medically trained)**<br>পাশ করা ডাক্তার (MBBS doctor)............................A<br>নার্স/ধাত্রী (Nurse/midwife) ....B<br>প্যারামেডিক/মেডিকেল এসিসটেন্ট/সাকমো (Paramedic/MA/SACMO)..................................C<br>পরিবার কল্যাণ পরিদর্শক (FWV).............................D<br>সি,এস,বি,এ (CSBA) ....E<br>**অন্যান্য স্বাস্থ্য কর্মী (Other health worker)**<br>স্বাস্থ্য সহকারী/ পরিবার কল্যান সহকারী (HA /FWA) ......F<br>পুষ্টি কর্মী (CNP)..G<br>সুসমা কার্যক্রমের স্বেচ্ছাসেবী (Volunteer of SUSOMA prj) ...H<br>অন্যান্য কমিউনিটি স্বাস্থ্য কর্মী - এনজিও কর্মী, স্বেচ্ছাসেবী (Other CHWs, NGO worker, volunteer)..................I<br>**অন্যান্য (Other)**<br>প্রশিক্ষণ প্রাপ্ত টিবিএ (প্রশিক্ষণ প্রাপ্ত ধন্নী, চাউনী, দাই) (TTBA) ....J<br>প্রশিক্ষণহীন টিবিএ (ধন্নী, চাউনী, দাই) TBA(Dai/Dhorni/Chauni) ....K<br>হোমিওপ্যাথ/হোমিওপ্যাথ ঔষধের দোকান (Homeopath/Homeopath drug store) ....L<br>আয়ুর্বেদিক চিকিৎসক / আয়ুর্বেদিক ঔষধের দোকান /হেকিম/কবিরাজ (Ayurved/ Ayurvedic drug store /Hekim/Kabiraj) ....M<br>গ্রাম ডাক্তার (Village doctor) ....N<br>এলোপ্যাথী ঔষধের দোকান (Allopath drug store) ....O<br>ইমাম/ঝাড় ফুক/ওঝা (Spiritual healer) ....P<br>পরিবারের অন্যান্য সদস্য/আত্মীয়/ প্রতিবেশী/বন্ধু Family/relative/Neighbor/friend............................Q<br>অন্যান্য Others________________________........X<br>(নির্দিষ্ট করুন)<br>কেউ অসেনি None came...Y<br>জানি না/মনে নাই Don't know/can't remember ....Z	 <br><br><br><br><br><br><br><br><br><br><br><br><br><br><br><br><br><br><br><br><br><br>**Y→ E23a**<br>**Z→ E23a**
E22	যে আপনার বাসায় এসেছিলেন আপনাকে দেখতে, সে জন্মের পর নবজাতকের যত্ন কিভাবে নিতে হবে সে সম্পর্কে বুঝানোর সময় কি কিছু দেখিয়েছিলেন? দেখিয়ে থাকলে, কি কি দেখিয়েছিল?<br>*[মহিলাকে জিজ্ঞেস করুন]* : আরও কিছু?<br><br>*মহিলার নিজে থেকে দেয়া সবগুলো উত্তরই বৃত্তায়িত করুন । উত্তরগুলো পড়ে শুনাবেন না । একাধিক উত্তর হতে পারে ।*<br><br>Did the person/health worker demonstrate immediate newborn care by showing you things? What did the health worker show you while talking about immediate newborn care?<br>[Do not read out the answers. Ask: Anything else? Circle all the answers]	পুতুল Doll .... A<br>ছবিওয়ালা কার্ড Card with pictures .... B<br>কাপড় Cloth .... C<br>অন্যান্য Others ____________________ .... D<br>নির্দিষ্ট করুন (Specify)<br>সে বুঝানোর জন্য কিছু দেখান নি<br>Didn't show me anything .... Y<br>মনে নাই Don't know/cant remember .... Z	

No.	Questions And Filters	Coding Categories	Skip
E23	যে আপনার বাসায় এসেছিলেন আপনাকে দেখতে, সে কি আপনাকে কিছু দিয়েছিলেন? উনি আপনাকে কি কি জিনিস দিয়েছিলেন?<br>*[মহিলাকে জিজ্ঞেস করুন]* : আরও কিছু?<br>*মহিলার নিজে থেকে দেয়া সবগুলো উত্তরই বৃত্তায়িত করুন। উত্তরগুলো পড়ে শুনাবেন না। একাধিক উত্তর হতে পারে।*<br><br>Did the health worker provide you anything during the visit?<br>[Do not read out the answers. Ask: Anything else? Circle all the answers]	রেফারেল স্লি- প Referral Slip .... A<br>ডেলিভারী সম্ভাব্য তারিখের কার্ড Delivery notification cardB<br>ডেলিভারী কিট Clean delivery kit .... C<br>আয়রন ট্যাবলেট/সিরাপ Iron Tablet/Syrup .... D<br>ভিজিএফ কার্ড (ভাউচার) VGF Card (Voucher) .... E<br>অন্যান্য Others ______________________ X<br>(নির্দিষ্ট করু ন)<br>কিছুই দেয় নি Nothing .... Y<br>মনে নাই Don't know/cant remember .... Z	
E23a	মাতৃ স্বাস্থ্যসেবা প্রদানে প্রশিক্ষণহীন দাই এর নতুন ভুমিকা সম্পর্কে আপনি অবগত আছেন কি?<br>Are you aware of the new role of TBAs regarding MNH services?	হ্যাঁ Yes .... 1<br>না No .... 2<br>জানি না/ মনে নাই Don't know/Can't remember 9	**→Supp Mod 2 (E23b)**
E24	শেষ গর্ভের সময়, নবজাতকের ধনুষ্টংকার অর্থাৎ জন্মের পর নবজাতকের খিচুনী প্রতিরোধের জন্য আপনি আপনার বাহুতে কোন ইনজেকশন দিয়েছিলেন কি?<br>During your most recent pregnancy did you receive an injection in the arm to prevent the baby from getting tetanus, that is convulsions after birth?	হ্যাঁ (Yes).. 1<br>না (No) .... 2<br>জানা নাই/মনে নাই DK/can't remember.................. 9	**2→ E26**<br>**9→ E26**
E25	শেষ গর্ভের সময়, কতবার আপনি এরকম ইনজেকশন নিয়েছিলেন?<br><br>During your most recent pregnancy , how many times did you receive such an injection?	একটি One... 1<br>দুইটি Two... 2<br>তিন বা তদুর্দ্ধ Three Or More .... 3<br>জানা নাই/মনে নাই DK/can't remember.................. 9	
E26	শেষ গর্ভের আগে আপনি কখনও কি TT ইনজেকশন নিয়েছিলেন যেমন আগের গর্ভের সময়ে অথবা দুই গর্ভের মধ্যবর্তী সময়ে অথবা জীবনের যে কোন সময়ে?<br>Did you receive any tetanus toxoid injection at any time before the most recent pregnancy, including previous pregnancy or between pregnancies?	হ্যাঁ (Yes).. 1<br>না (No) .... 2<br>জানা নাই/মনে নাই DK/can't remember.................. 9	**2→ E28**<br>**9→ E28**
E27	শেষ গর্ভের আগে মোট কতবার আপনি এরকম TT ইনজেকশন নিয়েছিলেন?<br><br>Before the recent pregnancy , how many times did you receive a tetanus injection?	একটি One... 1<br>দুইটি Two... 2<br>তিন বা তদুর্দ্ধ Three Or More .... 3<br>জানা নাই/মনে নাই DK/can't remember.................. 9	
E28	আপনার আত্মীয়দের মধ্য থেকে, শেষ গর্ভের সময় ডেলিভারীতে কেউ সাহায্য করেছিল কি?<br>Was there anyone of your family who assisted the delivery during most recent pregnancy?	হ্যাঁ Yes.. 1<br>না No .... 2<br>জানি না/ মনে নাই Don't know/Can't remember 9	**2→ E30**<br>**9→ E30**
E29	আপনার আত্মীয়দের মধ্য থেকে কে ডেলিভারীতে সাহায্য করেছিল?<br><br>*[মহিলাকে জিজ্ঞেস করেন,]* আরও কেউ? *[মহিলার নিজে থেকে দেয়া সবগুলো উত্তরই বৃত্তায়িত করুন । উত্তরগুলো পড়ে শুনাবেন না। একাধিক উত্তর হতে পারে।]*<br><br>Who had assisted the delivery from among your family members? [Don't prompt].	মা Mother .... A<br>শ্বাশুড়ী Mother-in-law .... B<br>বোন/ননদ/জা Sister .... C<br>চাচী/মামী/খালা/ফুফুAunt .... D<br>দাদী/নানী Grandmother .... E<br>ভাগনি/ভাতিজী Niece... F<br>স্বামী Husband .... G<br>অন্য কোন মহিলা আত্মীয় other female relative<br>________________ .... X<br>অন্য কোন পুরুষ আত্মীয় other male relative____________________......... Y	

No.	Questions And Filters	Coding Categories	Skip
E30	আপনার আত্মীয় নন এমন কেউ ডেলিভারীতে সাহায্য করেছিল কি?<br>Was there anyone from outside your family who assisted the delivery during most recent pregnancy?	হ্যাঁ Yes.. 1<br>না No.. 2<br>জানি না/ মনে নাই Don't know/Can't remember 9	**2→ E32**<br>**9→ E32**
E31	শেষ গর্ভের সময়, কে ডেলিভারীতে সাহায্য করেছিল?<br><br>*[মহিলাকে জিজ্ঞেস করেন,]* আরও কেউ? *[মহিলার নিজে থেকে দেয়া সবগুলো উত্তরই বৃত্তায়িত করুন । উত্তরগুলো পড়ে শুনাবেন না। একাধিক উত্তর হতে পারে।]*<br><br>*যদি উত্তরদাত্রী বলেন যে, কেউ ডেলিভারীতে সহায়তা করেননি তাহলে জিজ্ঞাসা কর‌ুন, প্রাপ্ত বয়স্ক কেউ ডেলিভারীর সময়ে উপস্থিত ছিল কিনা?*<br><br>Who assisted with the delivery during last pregnancy?<br><br>Anyone else?<br><br>PROBE FOR THE TYPE(S) OF PERSON(S) AND RECORD ALL MENTIONED.<br><br>IF RESPONDENT SAYS NO ONE ASSISTED, PROBE TO DETERMINE WHETHER ANY ADULTS WERE PRESENT AT THE DELIVERY.	**দক্ষ/প্রশিক্ষণ প্রাপ্ত স্বাস্থ্য কর্মী (Medically trained)**<br>পাশ করা ডাক্তার (MBBS doctor)........................ A<br>নার্স/ধাত্রী (Nurse/midwife) .... B<br>প্যারামেডিক/মেডিকেল এসিসটেন্ট/সাকমো (Paramedic/MA/SACMO)............................... C<br>পরিবার কল্যাণ পরিদর্শক (FWV)......................... D<br>সি,এস,বি,এ (CSBA) .... E<br>**অন্যান্য স্বাস্থ্য কর্মী (Other health worker)**<br>স্বাস্থ্য সহকারী/ পরিবার কল্যান সহকারী (HA /FWA) . F<br>পুষ্টি কর্মী (CNP).. G<br>সুসমা কার্যক্রমের স্বেচ্ছাসেবী (Volunteer of SUSOMA prj)H<br>অন্যান্য কমিউনিটি স্বাস্থ্য কর্মী - এনজিও কর্মী, স্বেচ্ছাসেবী (Other CHWs, NGO worker, volunteer) .... I<br>**অন্যান্য (Other)**<br>প্রশিক্ষণ প্রাপ্ত টিবিএ (প্রশিক্ষণ প্রাপ্ত ধন্নী, চাউনী, দাই) (TTBA) .... J<br>প্রশিক্ষণহীন টিবিএ (ধন্নী, চাউনী, দাই) TBA(Dai/Dhorni/Chauni) .... K<br>হোমিওপ্যাথ/হোমিওপ্যাথ ঔষধের দোকান (Homeopath/Homeopath drug store) .... L<br>আয়ুর্বেদিক চিকিৎসক / আয়ুর্বেদিক ঔষধের দোকান /হেকিম/কবিরাজ (Ayurved/ Ayurvedic drug store /Hekim/Kabiraj) .... M<br>গ্রাম ডাক্তার (Village doctor) .... N<br>এলোপ্যাথী ঔষধের দোকান (Allopath drug store) ... O<br>ইমাম/ঝাড় ফুক/ওঝা (Spiritual healer) .... P<br>পরিবারের অন্যান্য সদস্য/আত্মীয়/ প্রতিবেশী/বন্ধু Family/relative/Neighbor/friend........................ Q<br>অন্যান্য Others___________________________... X<br>(নির্দিষ্ট কর‌ুন)<br>জানি না/মনে নাই Don't know/can't remember .... Z	

No.	Questions And Filters	Coding Categories	Skip
E32	শেষ গর্ভের সময়, ডেলিভারীটি কোথায় হয়েছিল?<br><br>*(উত্তরগুলো পড়ে শুনাবেন না)*<br>[কেবলমাত্র একটি উত্তর হবে]<br><br>Where did the most recent delivery take place?<br><br>ONLY ONE ANSWER ALLOWED	**<u>সরকারী স্বাস্থ্য কেন্দ্র (Govt Health center)</u>**<br>মেডিকেল কলেজ হাসপাতাল (Medical College Hospital)....01<br>জেলা/সদর হাসপাতাল (District /Sadar Hospital)............02<br>মা ও শিশু স্বাস্থ্য কেন্দ্র (MCWC)..............................03<br>উপজেলা স্বাস্থ্য কমপেন্ডক্স (UHC)............................04<br>ইউনিয়ন স্বাস্থ্য ও পরিবার কল্যাণ কেন্দ্র/সাব সেন্টার/আরডি (FWC/SC/RD) ....05<br>কমিউনিটি ক্লিনিক (Community clinic)........................06<br>সেটেলাইট ক্লিনিক/ ইপিআই কেন্দ্র (Satellite clinic/EPI centre)..07<br>অন্যান্য সরকারী স্বাস্থ্য কেন্দ্র (Other Govt Health facility) ..08<br>**<u>বেসরকারী স্বাস্থ্য কেন্দ্র (Non Govt Health center)</u>**<br>এনজিও হাসপাতাল (NGO hospital) ....09<br>এনজিও স্থায়ী স্বাস্থ্য কেন্দ্র (NGO static health centre) ......10<br>এনজিও সেটেলাইট ক্লিনিক (NGO satellite clinic)...........11<br>পুষ্টি কেন্দ্র (NNP centre).......................................12<br>অন্যান্য বেসরকারী স্বাস্থ্য কেন্দ্র (Other NGO Health facility)...13<br>**<u>প্রাইভেট (Private Health sector)</u>**<br>হাসপাতাল/ ক্লিনিক (Hospital/clinic)..........................14<br>স্বাস্থ্য কেন্দ্র /ডিসপেনসারী (Health centre/Dispensary) .....15<br>এমবিবিএস ডাক্তারের চেম্বার (MBBS doctor's chamber) ...16<br>গ্রাম ডাক্তারের চেম্বার (Village doctor's chamber)...........17<br>প্যারামেডিক/মেডিকেল এসিসটেন্ট/সাকমোর চেম্বার (Paramedic/ MA/SACMO chamber) .... 18<br>এলোপ্যাথী ঔষধের দোকান (Allopath drug store) ....19<br>অন্যান্য প্রাইভেট স্বাস্থ্য কেন্দ্র (Other private Health facility)20<br><br>**<u>বাড়ী (Home)</u>**<br>নিজ বাড়ী, স্বামী/শ্বশুড় বাড়ী (Own home, husband/father in laws house).................21<br>বাবার বাড়ী (My natal home) ....22<br><br>অন্যান্য (Others______________________________..97<br>(নির্দিষ্ট করুন)<br>জানি না/মনে নাই Don't know/can't remember 99	
E32a	শেষ গর্ভের ডেলিভারীর সময় নিরাপদ ডেলিভারী কিট ব্যবহার করা হয়েছিল কি?<br><br>Was a clean delivery kit used during delivery of most recent pregnancy?	হ্যাঁ (Yes).. 1<br>না (No) .... 2<br>জানা নাই/মনে নাই DK/can't remember.................. 9	

No.	Questions And Filters	Coding Categories	Skip
E33	কখনও কখনও ডেলিভারীরর সময় কিছু বিপদ চিহ্ন দেখা যেতে পারে এবং তখন প্রসবকারী মাকে তাৎক্ষনিকভাবে স্বাস্থকেন্দ্রে পাঠানো দরকার হয়। ডেলিভারীরর সময়ে কি কি বিপদচিহ্ন দেখা দিলে একজন মহিলাকে তাৎক্ষনিকভাবে হাসপাতালে পাঠাতে হয় ?<br><br>*[মহিলাকে জিজ্ঞেস করুন]* আরও কোন সমস্যা/জটিলতা? *[মহিলার নিজে থেকে দেয়া সবগুলো উত্তরই বৃত্তায়িত করুন । উত্তরগুলো পড়ে শুনাবেন না। একাধিক উত্তর হতে পারে।]*<br><br>During delivery , once contraction started women may encounter severe problem or illness and should go or be taken immediately to a health facility.<br><br>While having contractions or delivering a baby, what types of symptoms would cause you to seek immediate care at a health facility right away?<br><br>Circle all signs mentioned, but do not prompt with any suggestions.	খিচুঁনী Convulsion.. A<br>তীব্র জ্বর Hight Fever... B<br>অতিরিক্ত রক্তস্রাব Excessive Vaginal Bleeding....... C<br>দ্রুত শ্বাস /শ্বাস নিতে কষ্ট হওয়া Fast/difficult breathing<br>দুর্গন্ধযুক্ত স্রাব A bad smelling vaginal discharge..... D<br>ফুল না পরা Retained Placenta........................... E<br>তীব্র মাথা ব্যথা/চোখে ঝাপসা দেখা Severe Headache/blurred vision .... F<br>দীর্ঘ / প্রলম্বিত প্রসব (12 ঘন্টার বেশী) ব্যথা থাকলে prolong labor 12 hr....................................... G<br>শিশুর হাত /পা আগে বের হয়ে এলে Hand or Feet came first... H<br>অন্যান্য Others____________________________ ..... X<br>(নির্দিষ্ট করুন)<br>জানা নাই/মনে নাই DK/can't remember.................. Z	
E34	আপনার শেষ গর্ভের ডেলিভারীর পর , ফুল পড়ার আগে, অতিরিক্ত রক্তক্ষরন না হওয়ার জন্য আপনি কি কোন ইঞ্জেকশন পেয়েছিলেন?<br><br>Immediately after delivery of your recent pregnancy, before the placenta was delivered, did you receive an injection to prevent you from bleeding too much?	হ্যাঁ (Yes).. 1<br>না (No) .... 2<br>জানা নাই/মনে নাই DK/can't remember.................. 9	
E35	ডেলিভারীতে সাহায্যকারী ব্যাক্তি ফুল বের হওয়ার জন্য আপনার পেটে চাপ দিয়ে নাড়ীতে টান দিয়েছিলেন কি?<br><br>Did the birth attendant hold your stomach and pull on the cord to help the placenta come out?	হ্যাঁ (Yes).. 1<br>না (No) .... 2<br>জানা নাই/মনে নাই DK/can't remember.................. 9	
E36	আপনার শেষ গর্ভের ডেলিভারীর পর , জড়ায়ু সংকোচনের মাধ্যমে রক্তক্ষরন কমানোর জন্য, ফুল পরার পরপরই কেউ আপনার পেটে মালিশ করেছিল কি?<br><br>During your recent pregnancy, immediately after the placenta was delivered , did someone massage your uterus to make it contract strongly and to prevent you from bleeding too much?	হ্যাঁ (Yes).. 1<br>না (No) .... 2<br>জানা নাই/মনে নাই DK/can't remember.................. 9	

No.	Questions And Filters	Coding Categories	Skip
E37	ডেলিভারীর পর একজন মায়ের কখনও কখনও এমন কিছু জটিলতা বা বিপদচিহ্ন দেখা যেতে পারে যে তখন তাকে অবিলম্বে চিকিৎসার জন্য হাসপাতালে/ ডাক্তারের কাছে নিয়ে যেতে হয়। ডেলিভারীর পর কি ধরনের লক্ষণ/ জটিলতা বা বিপদচিহ্ন দেখলে আপনি একজন মাকে চিকিৎসার জন্য হাসপাতালে বা স্বাস্থ্যকর্মীর কাছে নিয়ে যাবেন?<br><br>*[মহিলাকে জিজ্ঞেস করুন]* আরও কোন সমস্যা/জটিলতা?<br>*[মহিলার নিজে থেকে দেয়া সবগুলো উত্তরই বৃত্তায়িত করুন । উত্তরগুলো পড়ে শুনাবেন না। একাধিক উত্তর হতে পারে।]*<br><br>Sometimes mothers after delivery may have severe illness and should be taken immediately to a health facility.<br><br>What type of symptoms would cause you to go to a health facility right away?<br><br>Ask : Anything else?<br><br>CIRCLE ALL SIGNS MENTIONED, BUT DO NOT PROMPT	যোনীপথে অতিরিক্ত রক্তস্রাব<br>Excessive vaginal bleeding .... A<br>শ্বাস নিতে কষ্ট/ দ্রুত শ্বাস<br>Difficult breathing/ fast breathing .... B<br>অতিরিক্ত জ্বর হলে High fever .... C<br>পেটে তীব্র ব্যথা Severe abdominal pain.................. D<br>তীব্র মাথা ব্যথা/ঝাপসা দেখা<br>Severe headache/blurry vision .... E<br>খিঁচুনী / অজ্ঞান Convulsion/loss of conciousness..... F<br>যোনীপথে দূর্গন্ধযুক্ত স্রাব<br>Foul smelling vaginal discharge............................. G<br>পায়ের পিছনে ব্যথা Pain in calf .... H<br>আচরনগত পরিবর্তন অর্থাৎ যেখানে মা নিজেকে বা বাচ্চাকে আঘাত করতে পারে Behavior that indicates she may hurt herself or the baby.............. I<br>স্তন/স্তনবৃন্তে ফোলা, লাল হওয়া বা ব্যথা<br>swollen, red, tender breasts or nipples.................... J<br>প্রস্রাব করায় অসুবিধা বা প্রস্রাব ঝরা<br>problems urinating, or leaking .... K<br>পেরিনিয়াম এ ব্যাথা/ইনফেকশন<br>increased pain or infection in perineum .... L<br>ক্ষতস্থানে ব্যাথা/লাল ভাব/ পুঁজ হওয়া<br>infection in the area of wound (redness, swelling, pain, or pus in wound site .... M<br>অন্য কোন সমস্যা others___________________......... X<br>নির্দিষ্ট করুন<br>জানা নাই/মনে নাই DK/can't remember.................. Z	

No.	Questions And Filters	Coding Categories	Skip
E38	আপনার শেষ গর্ভের গর্ভকালীন সময়ে, ডেলিভারীর সময় বা ডেলিভারীর পর আপনার কি ______ হয়েছিল? (সমস্যা)<br>প্রত্যেকটি সমস্যা পড়ে শুনান:<br>a. তীব্র মাথা ব্যথাসহ চোখে ঝাপসা দেখা<br>b. খিচুনী/একলামশিয়া/অজ্ঞান হওয়া<br>c. উচ্চ রক্তচাপ<br>d. অতিরিক্ত রক্তস্রাব<br>e. পানি ভাঙ্গার ৬ ঘন্টা পরও প্রসব না হওয়া<br>f. বাচ্চার মাথা ছাড়া শরীরের অন্য অংশ আগে আসা<br>g. ১২ ঘন্টার অধিক প্রসব ব্যথা<br>h. ফুল না পড়া<br>i. দুর্গন্ধযুক্ত স্রাবের সাথে তীব্র জ্বর<br>j. হাতে/পায়ে/শরীরে পানি আসা<br>y. উপরে উলিণ্ঢখিত সমস্যাগুলোর কোনটাই হয়নি<br><br>Did you have any of the following problems during your recent pregnancy, delivery or period after delivery?<br><br>Read aloud the responses:<br><br>a. Blurred vision with severe headache<br>b. Convulsion/eclampsia/unconscious<br>c. High blood pressure<br>d. Excessive vaginal bleeding<br>e. Not delivered even after 6 hours of membrane rupture<br>f. Delivery of parts of the baby other than head<br>g. More than 12 hours of labor pain<br>h. Retained placenta<br>i. Foul smelling discharge with fever<br>j. Edema of hand/feet/body<br>y. None of the above mentined Problems	P= গর্ভাবস্থা (Pregnancy) , D= ডেলিভারীর সময় (Delivery), AD= ডেলিভারীর পর (After delivery)<br>সমস্যা P D AD<br>তীব্র মাথা ব্যথাসহ চোখে ঝাপসা দেখা<br>Blurred vision with severe headache ....A1........A2........A3<br>খিচুনী/একলামশিয়া/অজ্ঞান হওয়া<br>Convulsion/eclampsia/unconscious ....B1........B2........B3<br>উচ্চ রক্তচাপ<br>High blood pressure ....C1 ...... C2 ...... C3<br>অতিরিক্ত রক্তস্রাব<br>Excessive vaginal bleeding ....D1 ...... D2 ...... D3<br>পানি ভাঙ্গার ৬ ঘন্টা পরও প্রসব না হওয়া Not delivered even after 6 hours of membrane rupture ....E1 ..... -- ....--<br>বাচ্চার মাথা ছাড়া শরীরের অন্য অংশ আগে আসা Delivery of parts of the baby other than head..................--...........F2.........--<br>12 ঘন্টার অধিক প্রসব ব্যথা<br>More than 12 hours of labor pain.........--...........G2........--<br>ফুল না পড়া<br>Retained placenta ....--......... H2 ...... H3<br>দুর্গন্ধযুক্ত স্রাবের সাথে তীব্র জ্বর<br>Foul smelling discharge with fever--......... --......... I3<br>হাতে/পায়ে/শরীরে পানি আসা<br>Edema of hand/feet/body ....J1........J2........ J3<br>উপরে উলি- খিত সমস্যাগুলোর কোনটাই হয়নি None of the above mentined Problems ....Y1 ...... Y2 ...... Y3	**Y→ E43a**
E39	এই সমস্যা/জটিলতার জন্য আপনি কি কাউকে দেখিয়েছিলেন বা কারও সাহায্য নিয়েছিলেন?<br>Did you seek any sort of treatment for this problem/complication?	হ্যাঁ Yes .... 1<br>না No... 2<br>জানি না/ মনে নাই Don’t know/Can’t remember 9	**2→ E42**<br>**9→ E42**

No.	Questions And Filters	Coding Categories	Skip
E40	এই সমস্যা/জটিলতার জন্য আপনি কাকে দেখিয়েছিলেন বা কার সাহায্য নিয়েছিলেন?<br><br>*[মহিলাকে জিজ্ঞেস করুন]* আরও কেউ? *[মহিলার নিজে থেকে দেয়া সবগুলো উত্তরই বৃত্তায়িত করুন। উত্তরগুলো পড়ে শুনাবেন না। একাধিক উত্তর হতে পারে।]*<br><br>From whom did you seek treatment for this problem/complication? Do not read out the answers. Ask: Anything else? Circle all the answers.	**দক্ষ/প্রশিক্ষণ প্রাপ্ত স্বাস্থ্য কর্মী (Medically trained)**<br>পাশ করা ডাক্তার (MBBS doctor)........................ A<br>নার্স/ধাত্রী (Nurse/midwife) .... B<br>প্যারামেডিক/মেডিকেল এসিসটেন্ট/সাকমো (Paramedic/MA/SACMO).............................. C<br>পরিবার কল্যাণ পরিদর্শক (FWV)......................... D<br>সি,এস,বি,এ (CSBA) .... E<br>**অন্যান্য স্বাস্থ্য কর্মী (Other health worker)**<br>স্বাস্থ্য সহকারী/ পরিবার কল্যান সহকারী (HA /FWA) . F<br>পুষ্টি কর্মী (CNP)... G<br>সুসমা কার্যক্রমের স্বেচ্ছাসেবী (Volunteer of SUSOMA prj)H<br>অন্যান্য কমিউনিটি স্বাস্থ্য কর্মী - এনজিও কর্মী, স্বেচ্ছাসেবী (Other CHWs, NGO worker, volunteer)............. I<br>**অন্যান্য (Other)**<br>প্রশিক্ষণ প্রাপ্ত টিবিএ (প্রশিক্ষণ প্রাপ্ত ধন্নী, চাউনী, দাই) (TTBA) .... J<br>প্রশিক্ষণহীন টিবিএ (ধন্নী, চাউনী, দাই) TBA(Dai/Dhorni/Chauni) .... K<br>হোমিওপ্যাথ/হোমিওপ্যাথ ঔষধের দোকান (Homeopath/Homeopath drug store) .... L<br>আয়ুর্বেদিক চিকিৎসক / আয়ুর্বেদিক ঔষধের দোকান /হেকিম/কবিরাজ (Ayurved/ Ayurvedic drug store /Hekim/Kabiraj) .... M<br>গ্রাম ডাক্তার (Village doctor) .... N<br>এলোপ্যাথী ঔষধের দোকান (Allopath drug store) ... O<br>ইমাম/ঝাড় ফুক/ওঝা (Spiritual healer) .... P<br>পরিবারের অন্যান্য সদস্য/আত্মীয়/ প্রতিবেশী/বন্ধু Family/relative/Neighbor/friend....................... Q<br>অন্যান্য Others___________________________... X<br>(নির্দিষ্ট করুন)<br>জানি না/মনে নাই Don't know/can't remember .... Z	

No.	Questions And Filters	Coding Categories	Skip
E41	এই সমস্যা/জটিলতার জন্য আপনি কোথায় দেখিয়েছিলেন বা কোথায় সেবা পেয়েছিলেন?<br><br>*[মহিলাকে জিজ্ঞেস করুন]* আরও কোথাও?<br><br>*[মহিলার নিজে থেকে দেয়া সবগুলো উত্তরই বৃত্তায়িত করুন । উত্তরগুলো পড়ে শুনাবেন না। একাধিক উত্তর হতে পারে।]*<br>*[মহিলা যেখান থেকে সেবা পেয়েছেন, সেই স্বাস্থ্যকেন্দ্রের নাম লিখুন। যদি একাধিক জায়গা থেকে সেবা নিয়ে থাকেন, তাহলে সবগুলো জায়গারই নাম এবং কোড লিখুন।]*<br><br>Code :|____| নাম Name: ______________________________<br><br>Code :|____| নাম Name: ______________________________<br><br>Code :|____| নাম Name: ______________________________<br><br>Where did you go to seek care for this problem/complication?<br>Do not read out the answers. Ask: Anything else?<br>Circle all the answers. Write down code and name of place.	**<u>সরকারী স্বাস্থ্য কেন্দ্র (Govt Health center)</u>**<br>মেডিকেল কলেজ হাসপাতাল (Medical College Hospital)....A<br>জেলা/সদর হাসপাতাল (District /Sadar Hospital)...........B<br>মা ও শিশু স্বাস্থ্য কেন্দ্র (MCWC)..............................C<br>উপজেলা স্বাস্থ্য কমপেন্ডক্স (UHC)...........................D<br>ইউনিয়ন স্বাস্থ্য ও পরিবার কল্যাণ কেন্দ্র/সাব সেন্টার/আরডি (FWC/SC/RD)..E<br>কমিউনিটি ক্লিনিক (Community clinic).......................F<br>সেটেলাইট ক্লিনিক/ ইপিআই কেন্দ্র (Satellite clinic/EPI centre)...G<br>অন্যান্য সরকারী স্বাস্থ্য কেন্দ্র (Other Govt Health facility) ..H<br>**<u>বেসরকারী স্বাস্থ্য কেন্দ্র (Non Govt Health center)</u>**<br>এনজিও হাসপাতাল (NGO hospital) ....I<br>এনজিও স্থায়ী স্বাস্থ্য কেন্দ্র (NGO static health centre) ......J<br>এনজিও সেটেলাইট ক্লিনিক (NGO satellite clinic)..........K<br>পুষ্টি কেন্দ্র (NNP centre).......................................L<br>অন্যান্য বেসরকারী স্বাস্থ্য কেন্দ্র (Other NGO Health facility)..M<br>**<u>প্রাইভেট (Private Health sector)</u>**<br>হাসপাতাল/ ক্লিনিক (Hospital/clinic)..........................N<br>স্বাস্থ্য কেন্দ্র /ডিসপেনসারী (Health centre/Dispensary) .....O<br>এমবিবিএস ডাক্তারের চেম্বার (MBBS doctor's chamber) ...P<br>গ্রাম ডাক্তারের চেম্বার (Village doctor's chamber)...........Q<br>প্যারামেডিক/মেডিকেল এসিসটেন্ট/সাকমোর চেম্বার (Paramedic/ MA/SACMO chamber).. R<br>এলোপ্যাথী ঔষধের দোকান (Allopath drug store) ....S<br>অন্যান্য প্রাইভেট স্বাস্থ্য কেন্দ্র (Other private Health facility)T<br><br>**<u>বাড়ী (Home)</u>**<br>নিজ বাড়ী, স্বামী/শ্বশুড় বাড়ী (Own home, husband/father in laws house) ....U<br>বাবার বাড়ী (My natal home) ....V<br><br>অন্যান্য (Others______________________________..X<br>(নির্দিষ্ট করুন)<br>জানি না/মনে নাই Don't know/can't remember ....Z	
E42	সুসমা কার্যক্রমের কমিউনিটি গ্রুপ এই সমস্যা/ জটিলতার জন্য আপনাকে চিকিৎসা পেতে কোন সহায়তা করেছিল কি?<br><br>Did the community group of SUSOMA project help you in getting treatment for this problem/complication?	হ্যাঁ Yes .... 1<br>না No.. 2	**2→ E43a**
E43	সুসমা কার্যক্রমের কমিউনিটি গ্রুপ আপনাকে চিকিৎসা পেতে কি ভাবে সহায়তা করেছিল?<br><br>How did the community group of SUSOMA project help you in getting treatment?	__________________________________|___|___| <br>__________________________________|___|___|	
E43a	আপনার শেষ গর্ভের সময়ে, শিশু-মাতৃ স্বাস্থ্য বিষয়ক সেবা পাওয়ার ব্যাপারে স্বাস্থ্যসেবাদানকারীর সাথে আলাপচারিতায়/ব্যবহারে/আচরণে আপনি কি সন্তুষ্ট?<br>Are you satisfied with the interactions with health care providers when receiving MNH services?	হ্যাঁ Yes .... 1<br>না No .... 2<br>প্রযোজ্য নয়... 7<br>জানি না/ মনে নাই Don't know/Can't remember 9	

No.	Questions And Filters	Coding Categories	Skip
E44	শেষ গর্ভের ডেলিভারীর পর কোন স্বাস্থ্যকর্মী বা কোন প্রশিক্ষনহীন দাই স্বাস্থ্যকেন্দ্রে বা বাড়ীতে বা অন্য কোন স্থানে আপনার স্বাস্থ্য পরীক্ষা করেছিলেন কি?<br><br>Did a health care provider or atraditional birth attendant check on your health after the delivery of your recent pregnancy , either at a health facility , home or other location?	হ্যাঁ Yes.. 1<br>না No 2	**2→ F01**
E45	ডেলিভারীর কত সময় পর প্রথমবার আপনার স্বাস্থ্য পরীক্ষা করানো হয়েছিল?<br>*[যদি এক দিনের কম হয়, তাহলে 0 বৃত্তায়িত করে ঘন্টায় রেকর্ড করুন, যদি এক থেকে ছয় দিন হয়, তাহলে 1 বৃত্তায়িত করে দিনে রেকর্ড করুন , যদি ছয় দিনের বেশী হয়, তাহলে 2 বৃত্তায়িত করে সপ্তাহে রেকর্ড করুন]*<br><br>How long after the delivery did the first health check took place?<br><br>If less than one day, circle 0 and record hours; if one to six days circle 1 and record days; if more than 6 days circle 2 and record weeks.	ঘন্টা Hours ....0 |___|___| <br>দিন Days......................................1 |___|___| <br>সপ্তাহ Weeks ....2 |___|___| <br>জানি না Don’t know/can't remember.................. 999	
E46	শেষ গর্ভের সময়, জন্মের/ ডেলিভারীর পর কে আপনার স্বাস্থ্য পরীক্ষা করেছিল?<br><br>*[মহিলাকে জিজ্ঞেস করুন]* আরও কেউ? *[মহিলার নিজে থেকে দেয়া সবগুলো উত্তরই বৃত্তায়িত করুন । উত্তরগুলো পড়ে শুনাবেন না। একাধিক উত্তর হতে পারে।]*<br><br>Who checked your health at that time?<br><br>Do not read out the answers.<br>ASK: Anyone else?<br>Circle and write down all the answers	**<u>দক্ষ/প্রশিক্ষণ প্রাপ্ত স্বাস্থ্য কর্মী (Medically trained)</u>**<br>পাশ করা ডাক্তার (MBBS doctor)........................ A<br>নার্স/ধাত্রী (Nurse/midwife) .... B<br>প্যারামেডিক/মেডিকেল এসিসটেন্ট/সাকমো (Paramedic/MA/SACMO).............................. C<br>পরিবার কল্যাণ পরিদর্শক (FWV)........................ D<br>সি,এস,বি,এ (CSBA) .... E<br>**<u>অন্যান্য স্বাস্থ্য কর্মী (Other health worker)</u>**<br>স্বাস্থ্য সহকারী/ পরিবার কল্যান সহকারী (HA /FWA) . F<br>পুষ্টি কর্মী (CNP)... G<br>সুসমা কার্যক্রমের স্বেচ্ছাসেবী (Volunteer of SUSOMA prj)H<br>অন্যান্য কমিউনিটি স্বাস্থ্য কর্মী - এনজিও কর্মী, স্বেচ্ছাসেবী (Other CHWs, NGO worker, volunteer)............. I<br>**<u>অন্যান্য (Other)</u>**<br>প্রশিক্ষণ প্রাপ্ত টিবিএ (প্রশিক্ষণ প্রাপ্ত ধন্নী, চাউনী, দাই) (TTBA) .... J<br>প্রশিক্ষণহীন টিবিএ (ধন্নী, চাউনী, দাই) TBA(Dai/Dhorni/Chauni) .... K<br>হোমিওপ্যাথ/হোমিওপ্যাথ ঔষধের দোকান (Homeopath/Homeopath drug store) .... L<br>আয়ুর্বেদিক চিকিৎসক / আয়ুর্বেদিক ঔষধের দোকান /হেকিম/কবিরাজ (Ayurved/ Ayurvedic drug store /Hekim/Kabiraj) .... M<br>গ্রাম ডাক্তার (Village doctor) .... N<br>এলোপ্যাথী ঔষধের দোকান (Allopath drug store) ... O<br>ইমাম/ঝাড় ফুক/ওঝা (Spiritual healer) .... P<br>পরিবারের অন্যান্য সদস্য/আত্মীয়/ প্রতিবেশী/বন্ধু Family/relative/Neighbor/friend....................... Q<br>অন্যান্য Others________________________... X<br>(নির্দিষ্ট করুন)<br>জানি না/মনে নাই Don't know/can't remember .... Z	

No.	Questions And Filters	Coding Categories	Skip
E47	শেষ গর্ভের জন্মের/ ডেলিভারীর পর নিজের মেডিকেল চেক-আপের জন্য আপনি কোথায় দেখিয়েছিলেন/কোথায় করিয়েছিলেন?<br><br>Where did you receive a health check after the delivery of your most recent pregnancy?	**সরকারী স্বাস্থ্য কেন্দ্র (Govt Health center)**<br>মেডিকেল কলেজ হাসপাতাল (Medical College Hospital)....A<br>জেলা/সদর হাসপাতাল (District /Sadar Hospital)............B<br>মা ও শিশু স্বাস্থ্য কেন্দ্র (MCWC)..............................C<br>উপজেলা স্বাস্থ্য কমপেন্ডক্স (UHC)............................D<br>ইউনিয়ন স্বাস্থ্য ও পরিবার কল্যাণ কেন্দ্র/সাব সেন্টার/আরডি (FWC/SC/RD)..E<br>কমিউনিটি ক্লিনিক (Community clinic)........................F<br>সেটেলাইট ক্লিনিক/ ইপিআই কেন্দ্র (Satellite clinic/EPI centre)...G<br>অন্যান্য সরকারী স্বাস্থ্য কেন্দ্র (Other Govt Health facility) ..H<br>**বেসরকারী স্বাস্থ্য কেন্দ্র (Non Govt Health center)**<br>এনজিও হাসপাতাল (NGO hospital)..........................I<br>এনজিও স্থায়ী স্বাস্থ্য কেন্দ্র (NGO static health centre) ......J<br>এনজিও সেটেলাইট ক্লিনিক (NGO satellite clinic)...........K<br>পুষ্টি কেন্দ্র (NNP centre)......................................L<br>অন্যান্য বেসরকারী স্বাস্থ্য কেন্দ্র (Other NGO Health facility)..M<br>**প্রাইভেট (Private Health sector)**<br>হাসপাতাল/ ক্লিনিক (Hospital/clinic)..........................N<br>স্বাস্থ্য কেন্দ্র /ডিসপেনসারী (Health centre/Dispensary) .....O<br>এমবিবিএস ডাক্তারের চেম্বার (MBBS doctor's chamber) ...P<br>গ্রাম ডাক্তারের চেম্বার (Village doctor's chamber)...........Q<br>প্যারামেডিক/মেডিকেল এসিসটেন্ট/সাকমোর চেম্বার (Paramedic/ MA/SACMO chamber).. R<br>এলোপ্যাথী ঔষধের দোকান (Allopath drug store) ....S<br>অন্যান্য প্রাইভেট স্বাস্থ্য কেন্দ্র (Other private Health facility)T<br><br>**বাড়ী (Home)**<br>নিজ বাড়ী, স্বামী/শ্বশুড় বাড়ী (Own home, husband/father in laws house) ....U<br>বাবার বাড়ী (My natal home) ....V<br><br>অন্যান্য (Others________________________________..X<br>(নির্দিষ্ট করুন)<br>জানি না/মনে নাই Don't know/can't remember ....Z	**→Supp Mod 2 (E48, E49, E50, E51)**

Section F: Immediate newborn care

১ লা জানুয়ারী ২০১৩ সাল বা তার পরে গর্ভ ফলাফল হয়েছে এমন মহিলাকে এই প্রশ্নগুলো করতে হবে।

All questions are to be addressed to woman with pregnancy outcome since 01-January-2013

No.	Questions And Filters	Coding Categories	Skip
	**Immediate newborn care**		
F01	১ লা জানুয়ারী ২০১৩ সাল বা তার পরের গর্ভের ফলাফল কি ছিল?<br>What was the outcome of your pregnancy since 1 January 2013?	জীবিত জন্ম........1<br>মৃত জন্ম........2	**2→ F16a**
F02	১ লা জানুয়ারী ২০১৩ সাল বা তার পরে জন্ম হয়েছে এমন শিশুর নাম কি, লিঙ্গ কি এবং তার জন্ম তারিখ কত?<br><br>What is the date of the live birth that you had since January 1, 2013? What is the name and sex of the child?	জন্ম তারিখ |___|___|/|___|___|/|___|___| Date of birth দিন মাস বছর<br>নাম Name ____________<br>লিঙ্গ Sex<br>ছেলে Male........1<br>মেয়ে Female........2	
F03	জন্মের পর (নাম)-এর নাড়ী কি দিয়ে কাটা হয়েছিল?<br><br>[কেবলমাত্র একটি উত্তর হবে]<br><br>What instrument was used to cut the cord after the birth?<br>[Select only one answer]	ডেলিভারী ব্যাগ/কিট এর বেণ্ডড Blade from the delivery kit 01<br>নতুন ce-W New razor blade........02<br>নতুন এবং গরম পানিতে ফুটানো বেণ্ডড New and boiled razor blade........03<br>বাড়ীর পুরাতন বেণ্ডড Used razor blade........04<br>ব্যবহৃত এবং গরম পানিতে ফুটানো বেণ্ডড Used and boiled razor blade........05<br>নতুন কাঁচি Scissors........06<br>নতুন এবং গরম পানিতে ফুটানো কাঁচি New and boiled scissors........07<br>ব্যবহৃত কাঁচি Used scissors........08<br>ব্যবহৃত এবং গরম পানিতে ফুটানো কাঁচি Used and boiled scissors........09<br>ছুড়ি Knife........10<br>বাঁশের কঞ্চি/বাতা/টল Bamboo strips/ bata/toll ....11<br>অন্যান্য Others __________ ..97<br>(নির্দিষ্ট কর˜ন)<br>জানা নাই/মনে নাই Don't know/can't remember........99	
F04	নাড়ী কাটার আগে বা পরে (নাম)-এর নাড়িতে কোন কিছু দেয়া হয়েছিল কি?<br>Was anything placed on the umbilical cord of <Name> either before or after it was cut?	হ্যাঁ (Yes)........1<br>না (No)........2<br>জানা নাই/মনে নাই DK/can't remember........9	**2→** F06<br>**9→** F06

No.	Questions And Filters	Coding Categories	Skip
F05	নাড়ী কাটার পর (নাম)-এর নাড়ীতে কি দেয়া হয়েছিল? *[মহিলাকে জিজ্ঞেস করুন] আরও কিছু? [মহিলার নিজে থেকে দেয়া সবগুলো উত্তরই বৃত্তায়িত করুন । উত্তরগুলো পড়ে শুনাবেন না। একাধিক উত্তর হতে পারে।]* What was placed on the cut cord? *[Do not read out the answers],* *Ask: Anything else?* *Circle all the answers*	গোবর Cowdung .... A<br>যেকোন ধরনের তেল Any kind of oil .... B<br>অ্যান্টিসেপটিক (ডেটল/স্যাভলন/হেক্সাসল) Antiseptic (Detol/savlon/hexisol) .... C<br>ছাই Ash .... D<br>অ্যান্টিবায়োটিক (পাউডার/মলম) Antibiotics (Powder / Ointment) .... E<br>চিবানো চাল Chewed rice .... F<br>হলুদের রস/গুড়া Turmeric juice/powder .... G<br>আদার রস Ginger juice .... H<br>সিঁদুর Shidur .... I<br>বরিক পাউডার Boric powder .... J<br>জেনসিয়ান ভায়োলেট/নীল কালি Gentian violet/Blue ink K<br>ট্যালকম পাউডার Talcom Powder .... L<br>চুলার পোড়া মাটি Dust of earth-burner .... M<br>অন্যান্য Other ____________ .... X<br>(নির্দিষ্ট করুন)<br>জানা নাই/মনে নাই Don't know/can't remember...... Z	
F06	জন্মের পর পর অর্থাৎ ফুল পড়ার আগে (নাম)-কে মোছানো/শুকানো হয়েছিল কি? Was (NAME) dried (wiped) immediately after birth before the placenta was delivered?	হ্যাঁ (Yes) .... 1<br>না (No) .... 2<br>জানা নাই/মনে নাই DK/can't remember 9	**2**→ F08<br>**9**→ F08
F07	জন্মের কতক্ষন পর (নাম) কে মোছানো/শুকানো হয়েছিল? [এক ঘন্টার নীচে হলে মিনিটে লিখুন] How many minutes /hours after delivery of <Name> he/she was wiped/dried?	মিনিট Minutes .... 1 |___|___| ঘন্টা Hours .... 2 |___|___| জানি না Don't know/can't remember 999	
F08	জন্মের পর পর অর্থাৎ ফুল পড়ার আগে (নাম)-কে কাপড় দিয়ে মুড়িয়ে নেয়া হয়েছিল কি? Was (NAME) wrapped in a warm cloth or blanket immediately after birth before the placenta was delivered?	হ্যাঁ (Yes) .... 1<br>না (No) .... 2<br>জানা নাই/মনে নাই DK/can't remember 9	**2**→ F10<br>**9**→ F10
F09	জন্মের কতক্ষন পর (নাম) কে কাপড় দিয়ে মুড়িয়ে নেয়া হয়েছিল? [এক ঘন্টার নীচে হলে মিনিটে লিখুন] How many minutes after delivery of <Name> was wrapped?	মিনিট Minutes .... 1 |___|___| ঘন্টা Hours .... 2 |___|___| জানি না Don't know/can't remember 999	
F10	জন্মের এক ঘন্টার মধ্যে (নাম) এর চোখে কোন মলম বা ঔষধের ফোটা দেয়া হয়েছিল কি? In the first hour after delivery, was (NAME) given eye ointment or drops in his/her eyes?	হ্যাঁ (Yes) .... 1<br>না (No) .... 2<br>জানা নাই/মনে নাই DK/can't remember 9	
F11	জন্মের কত সময় পর (নাম)-কে প্রথম গোসল করানো হয়েছিল? *[যদি এক ঘন্টার কম হয়, তাহলে 1 বৃত্তায়িত করুন এবং ঘন্টার ঘরে 00 লিখুন, যদি এক দিনের কম হয়, তাহলে 1 বৃত্তায়িত করুন এবং ঘন্টার ঘরে রেকর্ড করুন, যদি এক সপ্তাহের কম হয়, তাহলে 2 বৃত্তায়িত করুন এবং দিনের ঘরে রেকর্ড করুন। যদি এক সপ্তাহের বেশী হয়, তাহলে 3 বৃত্তায়িত করুন এবং সপ্তাহের ঘরে রেকর্ড করুন]* How long after delivery was (NAME) bathed for the first time? *[If less than 1 hour circle 1 and write 00 in the box, if less than 1 day circle 1 and record hours , if less than 1 week circle 2 and record days , if more than 1 week circle 3 and record weeks]*	ঘন্টা Hours .... 1 |___|___| দিন Days .... 2 |___|___| সপ্তাহ Weeks .... 3 |___|___| গোসল করানো হয়নি Not bathed .... 997<br>জানি না/মনে নাই Don't know/can't remember......... 999	

No.	Questions And Filters	Coding Categories	Skip
F12	(নাম) এর ডেলিভারীর পর কোন স্বাস্থ্যকর্মী বা কোন প্রশিক্ষনহীন দাই (নাম) এর স্বাস্থ্য পরীক্ষা করেছিলেন কি?<br>After (Name) was born, did any health care provider or traditional birth attendant check on (Name's) health?	হ্যাঁ (Yes).. 1<br>না (No).. 2<br>জানা নাই/মনে নাই DK/can't remember.................. 9	**2**→ F16<br>**9**→ F16
F13	জন্মের কত সময় পর প্রথমবার (নাম) এর স্বাস্থ্য পরীক্ষা করানো হয়েছিল?<br>*[যদি এক দিনের কম হয়, তাহলে 0 বৃত্তায়িত করে ঘন্টায় রেকর্ড করুন, যদি এক থেকে ছয় দিন হয়, তাহলে 1 বৃত্তায়িত করে দিনে রেকর্ড করুন, যদি ছয় দিনের বেশী হয়, তাহলে 2 বৃত্তায়িত করে সপ্তাহে রেকর্ড করুন]*<br><br>How many hours, days or weeks after the birth of (Name) did the first health check take place?<br><br>If less than one day, circle 0 and record hours; if one to six days circle 1 and record days; if more than 6 days circle 2 and record weeks.	ঘন্টা Hours... 0 |___|___| <br>দিন Days .... 1 |___|___| <br>সপ্তাহ Weeks.. 2 |___|___| <br>জানি না Don't know/can't remember..... 999	
F14	ডেলিভারীর পর কে (নাম) এর স্বাস্থ্য পরীক্ষা করেছিল?<br><br>[মহিলাকে জিজ্ঞেস *করুন*] আরও কেউ? সবচেয়ে দক্ষ স্বাস্থ্যকর্মীর খোজ করুন[মহিলার নিজে থেকে দেয়া সবগুলো উত্তরই বৃত্তায়িত *করুন* ।<br><br>উত্তরগুলো পড়ে শুনাবেন না। একাধিক উত্তর হতে পারে।]<br><br>Who checked on (Name's) health at that time?<br><br>Anyone else?<br><br>Probe for the most qualified person and record all mentioned.	**<u>দক্ষ/প্রশিক্ষণ প্রাপ্ত স্বাস্থ্য কর্মী (Medically trained)</u>**<br>পাশ করা ডাক্তার (MBBS doctor)........................ A<br>নার্স/ধাত্রী (Nurse/midwife) .... B<br>প্যারামেডিক/মেডিকেল এসিসটেন্ট/সাকমো (Paramedic/MA/SACMO)............................... C<br>পরিবার কল্যাণ পরিদর্শক (FWV)......................... D<br>সি,এস,বি,এ (CSBA) .... E<br>**<u>অন্যান্য স্বাস্থ্য কর্মী (Other health worker)</u>**<br>স্বাস্থ্য সহকারী/ পরিবার কল্যান সহকারী (HA /FWA) . F<br>পুষ্টি কর্মী (CNP).. G<br>সুসমা কার্যক্রমের স্বেচ্ছাসেবী (Volunteer of SUSOMA prj)H<br>অন্যান্য কমিউনিটি স্বাস্থ্য কর্মী - এনজিও কর্মী, স্বেচ্ছাসেবী (Other CHWs, NGO worker, volunteer).............. I<br>**<u>অন্যান্য (Other)</u>**<br>প্রশিক্ষণ প্রাপ্ত টিবিএ (প্রশিক্ষণ প্রাপ্ত ধন্নী, চাউনী, দাই) (TTBA) .... J<br>প্রশিক্ষণহীন টিবিএ (ধন্নী, চাউনী, দাই) TBA(Dai/Dhorni/Chauni) .... K<br>হোমিওপ্যাথ/হোমিওপ্যাথ ঔষধের দোকান (Homeopath/Homeopath drug store) .... L<br>আয়ুর্বেদিক চিকিৎসক / আয়ুর্বেদিক ঔষধের দোকান /হেকিম/কবিরাজ (Ayurved/ Ayurvedic drug store /Hekim/Kabiraj) .... M<br>গ্রাম ডাক্তার (Village doctor) .... N<br>এলোপ্যাথী ঔষধের দোকান (Allopath drug store) ... O<br>ইমাম/ঝাড় ফুক/ওঝা (Spiritual healer) .... P<br>পরিবারের অন্যান্য সদস্য/আত্মীয়/ প্রতিবেশী/বন্ধু Family/relative/Neighbor/friend........................ Q<br>অন্যান্য Others_________________________... X<br>(নির্দিষ্ট *করুন*)<br>জানি না/মনে নাই Don't know/can't remember .... Z	

No.	Questions And Filters	Coding Categories	Skip
F15	ডেলিভারীর পর কোথায় (নাম) এর স্বাস্থ্য পরীক্ষা করিয়েছিলেন?<br><br>[মহিলার নিজে থেকে দেয়া সবগুলো উত্তরই বৃত্তায়িত করুন। উত্তরগুলো পড়ে শুনাবেন না। একাধিক উত্তর হতে পারে।]<br><br>From where did you receive health checkup for name?<br>Do not readout the answers. Ask anywhere else? Record all the answers	**<u>সরকারী স্বাস্থ্য কেন্দ্র (Govt Health center)</u>**<br>মেডিকেল কলেজ হাসপাতাল (Medical College Hospital)....A<br>জেলা/সদর হাসপাতাল (District /Sadar Hospital)............B<br>মা ও শিশু স্বাস্থ্য কেন্দ্র (MCWC).............................C<br>উপজেলা স্বাস্থ্য কমপেন্ডক্স (UHC)...........................D<br>ইউনিয়ন স্বাস্থ্য ও পরিবার কল্যাণ কেন্দ্র/সাব সেন্টার/আরডি (FWC/SC/RD)..E<br>কমিউনিটি ক্লিনিক (Community clinic)........................F<br>সেটেলাইট ক্লিনিক/ ইপিআই কেন্দ্র (Satellite clinic/EPI centre)..G<br>অন্যান্য সরকারী স্বাস্থ্য কেন্দ্র (Other Govt Health facility) ..H<br>**<u>বেসরকারী স্বাস্থ্য কেন্দ্র (Non Govt Health center)</u>**<br>এনজিও হাসপাতাল (NGO hospital) ....I<br>এনজিও স্থায়ী স্বাস্থ্য কেন্দ্র (NGO static health centre) ......J<br>এনজিও সেটেলাইট ক্লিনিক (NGO satellite clinic)...........K<br>পুষ্টি কেন্দ্র (NNP centre).......................................L<br>অন্যান্য বেসরকারী স্বাস্থ্য কেন্দ্র (Other NGO Health facility)..M<br>**<u>প্রাইভেট (Private Health sector)</u>**<br>হাসপাতাল/ ক্লিনিক (Hospital/clinic)..........................N<br>স্বাস্থ্য কেন্দ্র /ডিসপেনসারী (Health centre/Dispensary) .....O<br>এমবিবিএস ডাক্তারের চেম্বার (MBBS doctor’s chamber) ...P<br>গ্রাম ডাক্তারের চেম্বার (Village doctor’s chamber)............Q<br>প্যারামেডিক/মেডিকেল এসিসটেন্ট/সাকমোর চেম্বার (Paramedic/ MA/SACMO chamber)... R<br>এলোপ্যাথী ঔষধের দোকান (Allopath drug store) ....S<br>অন্যান্য প্রাইভেট স্বাস্থ্য কেন্দ্র (Other private Health facility)T<br><br>**<u>বাড়ী (Home)</u>**<br>নিজ বাড়ী, স্বামী/শ্বশুড় বাড়ী (Own home, husband/father in laws house) ....U<br>বাবার বাড়ী (My natal home) ....V<br><br>অন্যান্য (Others________________________________..X<br>(নির্দিষ্ট করুন)<br>জানি না/মনে নাই Don't know/can't remember ....Z	→Supp Mod 2 (F 15 a, b, c,d)

No.	Questions And Filters	Coding Categories	Skip
F16	কখনও কখনও নবজাতক বাচ্চা একমাস বয়সের মধ্যেই খুব অসুস্থ হয়ে যায় এবং তখন তাদেরকে অবিলম্বে চিকিৎসার জন্য হাসপাতালে/ ডাক্তারের কাছে নিয়ে যেতে হয়। কি ধরনের লক্ষণ দেখলে আপনি আপনার নবজাতককে সাথে সাথেই চিকিৎসার জন্য হাসপাতালে বা ডাক্তারের কাছে নিয়ে যাবেন?<br><br>*[মহিলাকে জিজ্ঞেস কর^ন]* আরও কোন সমস্যা/জটিলতা? *[মহিলার নিজে থেকে দেয়া সবগুলো উত্তরই বৃত্তায়িত করুন । উত্তরগুলো পড়ে শুনাবেন না। একাধিক উত্তর হতে পারে।]*<br><br>Sometimes newborns, within the first month of life, have severe illness and should be taken immediately to a health facility.<br><br>What types of symptoms would cause you to take your newborn to a health facility right away?<br><br>ASK: Anything else?<br><br>Do not read responses. Record all that are mentioned.	খিঁচুনি বা শরীর শক্ত হওয়া Convulsions.................... A<br>জ্বর/বাচ্চার শরীর গরম হওয়া Baby feels hot/Fever ... B<br>বুকের দুধ চুষতে না পারা Poor suckling or feeding ... C<br>কষ্টকর/দ্রু^ত শ্বাস নেয়া Difficult or fast breathing.... D<br>বাচ্চার শরীর ঠান্ডা হওয়া Baby feels cold .... E<br>বাচ্চা খুব ছোট হওয়া Too small baby/too early......... F<br>হাতের তালু/ পায়ের পাতা/চোখ হলুদ হওয়া/জন্ডিস Yellow palm/soles/eye color (jaundice) .... G<br>পেট ফাঁপা বা ফোলা Sowllen abdomen .... H<br>অজ্ঞান/হুঁশ না থাকা Unconsciousness .... I<br>নাভির চারপাশ,চোখ অথবা চামড়া লাল হওয়া/পুঁজ বের হওয়া Pus or redness of the umbilical stump,eyes or skin.. J<br>রক্তপাত Bleeding... K<br>ডায়রিয়া Diarrhoea.. L<br>মুখের ভিতর ঘা/সাদা দাগ Ulcers or thrush (white patches in mouth)............... M<br>অন্যান্য Others ___________________________ ... X<br>(নির্দিষ্ট কর^ন)<br>জানা নাই (Don't know).................................. Z	
F16a	আপনার শেষ গর্ভের সময়ে, শিশু-মাতৃ স্বাস্থ্য বিষয়ক সেবা পাওয়ার ব্যাপারে/সেবা নিতে যাওয়ার ক্ষেত্রে আপনি কোন বাধা পেয়েছিলেন?<br><br>Did you encounter any difficulties accessing MNH services?	হ্যাঁ Yes .... 1<br>না No .... 2<br>প্রযোজ্য নয়.. 9	2→ F17<br>9→ F17
F16b	কি ধরনের বাধা পেয়েছিলেন?<br><br>What sort of difficulties you encountered?	______________________________________|___|___| ______________________________________|___|___| ______________________________________|___|___| ______________________________________|___|___|	**→Supp Mod 2 (F16c, F16d)**
**তথ্যসংগ্রহকরীর জন্য নির্দেশিকা** Instruction for interviewer**: CHECK Questions**			
F17	১ লা জানুয়ারী ২০১৩ সাল বা তার পরের গর্ভ ফলাফলের জন্য আপনাকে কি এই *মহিলার স্বামীর* নিকট হতে Husband's module (Module X) এর জন্য তথ্য সংগ্রহ করতে বলা হয়েছে?<br>Were you asked tocollect information from women's husband (Module X)?	হ্যাঁ Yes .... 1<br><br>না No 2	**→Module X**
F19	উত্তর দাতাকে ধন্যবাদ জানিয়ে সাক্ষাৎকার শেষ কর^ন।<br>Thanks the mother for providing time and complete the interview.		

Annex 2c:

Evaluation of the project, ‘Working with Individuals, Families and Communities (IFC) to improve maternal and newborn health in Netrokona district, Bangladesh’

IFC Framework Evaluation Baseline Survey 2014

Module X

Husbands awareness module

ALL QUESTIONS ARE TO BE ADDRESSED TO THE HUSBAND OF WOMEN WITH A PREGNANCY OUTCOME SINCE 1’st JANUARY 2013

	Name	Code	
CLUSTER/UNION		☐☐	সাক্ষাৎকার শুরু হবার সময়: |___|___|:|___|___|
UPAZILA		☐☐	
VILLAGE		☐☐☐☐	
HOUSEHOLD ID		☐☐☐☐	সাক্ষাৎকার শেষ করার সময় |___|___|:|___|___|
WOMEN ID		☐☐☐	
HUSBAND			

Section X: Husband's role in Maternal Care

No.	Questions And Filters	Coding Categories	Skip
			→Supp Mod X X00a, X00b, X00c)
X01	গর্ভকালীন সময়ে একজন মহিলার মানসম্মত মাতৃ স্বাস্থ্যসেবা পাওয়ার অধিকার রয়েছে, এ ব্যাপারে আপনি জানেন কি?<br>Are you aware that a pregnant women has the right to access quality MNH services?	হ্যাঁ Yes .... 1<br>না No .... 2<br>জানি না/ মনে নাই Don't know/Can't remember 9	
X02	গর্ভকালীন সময়ে একজন মহিলা অসুস্থ না হলেও মেডিকেল চেকআপ (ANC) করার দরকার আছে কি?<br>Do you know about ANC (Ante natal care), a medical check-up for a woman during pregnancy , even when she is not sick?	হ্যাঁ Yes .... 1<br>না No .... 2<br>জানি না/ মনে নাই Don't know/Can't remember 9	2→ X04<br>9→ X04
X03	গর্ভকালীন সময়ে একজন মহিলার কমপক্ষে কত বার মেডিকেল চেকআপ (ANC) করা দরকার?<br>Can you tell me at least how many times a pregnant woman should receive such kind of medical check-up (ANC) during pregnancy?	বার (গর্ভকালীন সময়ে) .... |____|____| Times (during pregnancy)<br>জানি না/ মনে নাই Don't know/Can't remember 9	
X04	শেষ গর্ভের সময়, গর্ভকালীন মেডিকেল চেক-আপের জন্য আপনার স্ত্রী কি কাউকে দেখিয়েছিলেন?<br>Did your wife see (consult) anyone for antenatal care during her most recent pregnancy?	হ্যাঁ Yes .... 1<br>না No .... 2<br>জানি না/ মনে নাই Don't know/Can't remember 9	2→ X10<br>9→ X10
X05	শেষ গর্ভের সময়, গর্ভকালীন মেডিকেল চেক-আপের জন্য আপনার স্ত্রী কাকে দেখিয়েছিলেন?<br>*[জিজ্ঞেস করুন]* আরও কিছু? *[মহিলার স্বামীর নিজে থেকে দেয়া সবগুলো উত্তরই বৃত্তায়িত করুন]* । *উত্তরগুলো পড়ে শুনাবেন না। একাধিক উত্তর হতে পারে।]*<br>Whom did your wife see for antenatal care during her most recent pregnancy? [Do not readout the answers. Ask anyone else? Record all the answers]	**<u>দক্ষ/প্রশিক্ষণ প্রাপ্ত স্বাস্থ্য কর্মী (Medically trained)</u>**<br>পাশ করা ডাক্তার (MBBS doctor)........ A<br>নার্স/ধাত্রী (Nurse/midwife) .... B<br>প্যারামেডিক/মেডিকেল এসিসটেন্ট/সাকমো (Paramedic/MA/SACMO)........ C<br>পরিবার কল্যাণ পরিদর্শক (FWV)........ D<br>সি,এস,বি,এ (CSBA) .... E<br>**<u>অন্যান্য স্বাস্থ্য কর্মী (Other health worker)</u>**<br>স্বাস্থ্য সহকারী/ পরিবার কল্যান সহকারী (HA /FWA) . F<br>পুষ্টি কর্মী (CNP)........ G<br>সুসমা কার্যক্রমের স্বেচ্ছাসেবী (Volunteer of SUSOMA prj)H<br>অন্যান্য কমিউনিটি স্বাস্থ্য কর্মী - এনজিও কর্মী, স্বেচ্ছাসেবী (Other CHWs, NGO worker, volunteer)........ I<br>**<u>অন্যান্য (Other)</u>**<br>প্রশিক্ষণ প্রাপ্ত টিবিএ (প্রশিক্ষণ প্রাপ্ত ধন্নী, চাউনী, দাই) (TTBA) .... J<br>প্রশিক্ষণহীন টিবিএ (ধন্নী, চাউনী, দাই) TBA(Dai/Dhorni/Chauni) .... K<br>হোমিওপ্যাথ/হোমিওপ্যাথ ঔষধের দোকান (Homeopath/Homeopath drug store) .... L<br>আয়ুর্বেদিক চিকিৎসক / আয়ুর্বেদিক ঔষধের দোকান /হেকিম/কবিরাজ (Ayurved/ Ayurvedic drug store /Hekim/Kabiraj) .... M<br>গ্রাম ডাক্তার (Village doctor) .... N<br>এলোপ্যাথী ঔষধের দোকান (Allopath drug store) ... O<br>ইমাম/ঝাড় ফুক/ওঝা (Spiritual healer) .... P<br>পরিবারের অন্যান্য সদস্য/আত্মীয়/ প্রতিবেশী/বন্ধু Family/relative/Neighbor/friend........ Q<br>অন্যান্য Others__________________... X<br>(নির্দিষ্ট করুন)<br>জানি না/মনে নাই Don't know/can't remember .... Z	

No.	Questions And Filters	Coding Categories	Skip
	→Supp Mod X X00a, X00b, X00c)		
X06	শেষ গর্ভের সময়, গর্ভকালীন মেডিকেল চেক-আপ আপনার স্ত্রী কোথায় নিয়েছিলেন/করিয়েছিলেন?<br><br>*[জিজ্ঞেস করুন]* আরও কিছু? *[মহিলার স্বামীর নিজে থেকে দেয়া সবগুলো উত্তরই বৃত্তায়িত করুন । উত্তরগুলো পড়ে শুনাবেন না। একাধিক উত্তর হতে পারে।]*<br><br>*[মহিলা যেখান থেকে সেবা পেয়েছেন, সেই স্বাস্থ্যকেন্দ্রের নাম লিখুন। যদি একাধিক জায়গা থেকে সেবা নিয়ে থাকেন, তাহলে সবগুলো জায়গারই নাম এবং কোড লিখুন।]*<br><br>Code :|____| নাম Name: ____________________________<br><br>Code :|____| নাম Name: ____________________________<br><br>Code :|____| নাম Name: ____________________________<br><br>Where did your wife receive antenatal care for her most recent pregnancy? Do not readout the answers. Ask anywhere else? Record all the answers. Record the name of the places where the women sought care.	**সরকারী স্বাস্থ্য কেন্দ্র (Govt Health center)**<br>মেডিকেল কলেজ হাসপাতাল (Medical College Hospital)....A<br>জেলা/সদর হাসপাতাল (District /Sadar Hospital)...........B<br>মা ও শিশু স্বাস্থ্য কেন্দ্র (MCWC).............................C<br>উপজেলা স্বাস্থ্য কমপেক্স (UHC)...........................D<br>ইউনিয়ন স্বাস্থ্য ও পরিবার কল্যাণ কেন্দ্র/সাব সেন্টার/আরডি (FWC/SC/RD)...E<br>কমিউনিটি ক্লিনিক (Community clinic).......................F<br>সেটেলাইট ক্লিনিক/ ইপিআই কেন্দ্র (Satellite clinic/EPI centre)..G<br>অন্যান্য সরকারী স্বাস্থ্য কেন্দ্র (Other Govt Health facility) ..H<br>**বেসরকারী স্বাস্থ্য কেন্দ্র (Non Govt Health center)**<br>এনজিও হাসপাতাল (NGO hospital) ....I<br>এনজিও স্থায়ী স্বাস্থ্য কেন্দ্র (NGO static health centre) ......J<br>এনজিও সেটেলাইট ক্লিনিক (NGO satellite clinic)...........K<br>পুষ্টি কেন্দ্র (NNP centre).....................................L<br>অন্যান্য বেসরকারী স্বাস্থ্য কেন্দ্র (Other NGO Health facility)..M<br>**প্রাইভেট (Private Health sector)**<br>হাসপাতাল/ ক্লিনিক (Hospital/clinic).........................N<br>স্বাস্থ্য কেন্দ্র /ডিসপেনসারী (Health centre/Dispensary) .....O<br>এমবিবিএস ডাক্তারের চেম্বার (MBBS doctor's chamber) ...P<br>গ্রাম ডাক্তারের চেম্বার (Village doctor's chamber)...........Q<br>প্যারামেডিক/মেডিকেল এসিসটেন্ট/সাকমোর চেম্বার (Paramedic/ MA/SACMO chamber).. R<br>এলোপ্যাথী ঔষধের দোকান (Allopath drug store) ....S<br>অন্যান্য প্রাইভেট স্বাস্থ্য কেন্দ্র (Other private Health facility)T<br><br>**বাড়ী (Home)**<br>নিজ বাড়ী, স্বামী/শ্বশুড় বাড়ী (Own home, husband/father in laws house)..U<br>বাবার বাড়ী (My natal home) ....V<br><br>অন্যান্য (Others______________________________..X<br>(নির্দিষ্ট করুন)<br>জানি না/মনে নাই Don't know/can't remember ....Z	U→ X08<br>V→ X08
X07	শেষ গর্ভের সময়, গর্ভকালীন মেডিকেল চেক-আপের জন্য আপনি আপনার স্ত্রীর সাথে গিয়েছিলেন কি?<br><br>Did you accompany your wife while receiving a health check (ANC) during her most recent pregnancy?	হ্যাঁ Yes.. 1<br>না No.. 2	
X08	আপনার স্ত্রী যখন প্রথম গর্ভকালীন মেডিকেল চেক-আপ করিয়েছিলেন/নিয়েছিলেন, তখন তিনি কত মাসের গর্ভবতী ছিলেন?<br>How many months pregnant were your wife when she first received antenatal care?	মাসের গর্ভবতী Month-pregnant (during 1st check-up)...................................|_____|_____| <br>জানা নাই/মনে নাই DK/can't remember............... 99	

No.	Questions And Filters	Coding Categories	Skip
	→Supp Mod X X00a, X00b, X00c)		
X09	*[Question X05 দেখুন, code A থেকে E এর যে কোন একটি বা একাধিক বৃত্তায়িত থাকলে কোড/কোডগুলো এখানে লিখুন: _____ , _____, ____ । এবার কোড/কোডগুলো দেখে দেখে মহিলাকে প্রশ্ন কর ন ঃ]*<br>আপনার স্ত্রী এর/এদের কাছ থেকে মোট কতবার গর্ভকালীন মেডিকেল চেক-আপ করিয়েছিলেন /নিয়েছিলেন?<br>How many times did your wife receive antenatal care from for this pregnancy from the mentioned skilled health care provides (Code A-E of Q X05)?	বার Number .... |____|____| প্রযোজ্য নয় Not applicable .... 97<br>জানা নাই/মনে নাই DK/can't remember 99	
X10	কখনও কখনও গর্ভকালীন সময় কিছু বিপদ চিহ্ন দেখা যেতে পারে এবং তখন গর্ভবতীকে তাৎক্ষনিকভাবে স্বাস্থকেন্দ্রে পাঠানো দরকার হয়।<br>গর্ভকালীন সময়ে কি কি বিপদচিহ্ন দেখা দিলে একজন মহিলাকে তাৎক্ষনিকভাবে হাসপাতালে পাঠাতে হয় ?<br>*[মহিলার স্বামীকে জিজ্ঞেস কর ন]* আরও কোন সমস্যা/জটিলতা? *[মহিলার স্বামীর নিজে থেকে দেয়া সবগুলো উত্তরই বৃত্তায়িত কর ন । উত্তরগুলো পড়ে শুনাবেন না। একাধিক উত্তর হতে পারে।]*<br>During pregnancy, women may encounter severe problems or illness and should go or be taken immediately to a health facility.<br>What types of symptoms would cause you to seek care at a health facility right away?<br>Ask: Anything else?<br>Circle all signs mentioned, but do not prompt with any suggestions.	যোনীপথে রক্তস্রাব Vaginal Bleeding ....A<br>দ্র ত শ্বাস /শ্বাস নিতে কষ্ট হওয়া Fast/difficult breathing....B<br>জ্বর Fever ....C<br>তলপেটে তীব্র ব্যথা Severe abdominal pain ....D<br>মাথা ব্যথা চোখে ঝাপসা দেখা<br>Headache/ Blurred Vision ....E<br>খিচুনী/ফিট Convulsions/fits ....F<br>দুর্গন্ধযুক্ত স্রাব Foul smelling vaginal discharge ....G<br>গর্ভের বাচ্চার নড়াচড়া কমে যাওয়া/বন্ধ হওয়া<br>Fetal movement reduced/absent ....H<br>যোনীপথ দিয়ে ঘোলাটে/সবুজাভ কিছু বের হওয়া Leaking brownish/greenish fluid from the vagina ....<br>হাতে পানি আসা/আঙ্গুল ফুলে যাওয়া Edema of hand/fingers ....<br>মুখে / পায়ে পানি আসা Edema of face/ leg.................K<br>অন্যান্য Others ________________________....X<br>নির্দিষ্ট কর ন<br>জানা নাই/মনে নাই DK/can't remember.......................Z	
X11	শেষ গর্ভের সময় আপনার স্ত্রীর, বাচ্চা হওয়া এবং জরুরী অবস্থা বিষয়ক পরিকল্পনার জন্য কোন স্বাস্থ্যকর্মীর সাথে আলাপ/পরামর্শ করেছিলেন কি?<br>Did you discuss a plan for birth and emergencies with a health care worker?	হ্যাঁ Yes .... 1<br>না No .... 2<br>জানি না/ মনে নাই Don't know/Can't remember 9	2→ X13<br>9→ X13

No.	Questions And Filters	Coding Categories	Skip
	→Supp Mod X X00a, X00b, X00c)		
X12	কার সাথে আলাপ/পরামর্শ করেছিলেন?<br><br>With whom?	**দক্ষ/প্রশিক্ষণ প্রাপ্ত স্বাস্থ্য কর্মী (Medically trained)**<br>পাশ করা ডাক্তার (MBBS doctor)............................A<br>নার্স/ধাত্রী (Nurse/midwife) ....B<br>প্যারামেডিক/মেডিকেল এসিসটেন্ট/সাকমো (Paramedic/MA/SACMO)..................................C<br>পরিবার কল্যাণ পরিদর্শক (FWV).............................D<br>সি,এস,বি,এ (CSBA) ....E<br>**অন্যান্য স্বাস্থ্য কর্মী (Other health worker)**<br>স্বাস্থ্য সহকারী/ পরিবার কল্যান সহকারী (HA /FWA) ......F<br>পুষ্টি কর্মী (CNP)...G<br>সুসমা কার্যক্রমের স্বেচ্ছাসেবী (Volunteer of SUSOMA prj)H<br>অন্যান্য কমিউনিটি স্বাস্থ্য কর্মী - এনজিও কর্মী, স্বেচ্ছাসেবী (Other CHWs, NGO worker, volunteer)..................I<br>**অন্যান্য (Other)**<br>প্রশিক্ষণ প্রাপ্ত টিবিএ (প্রশিক্ষণ প্রাপ্ত ধন্নী, চাউনী, দাই) (TTBA) ....J<br>প্রশিক্ষণহীন টিবিএ (ধন্নী, চাউনী, দাই) TBA(Dai/Dhorni/Chauni) ....K<br>হোমিওপ্যাথ/হোমিওপ্যাথ ঔষধের দোকান (Homeopath/Homeopath drug store) ....L<br>আয়ুর্বেদিক চিকিৎসক / আয়ুর্বেদিক ঔষধের দোকান /হেকিম/কবিরাজ (Ayurved/ Ayurvedic drug store /Hekim/Kabiraj) ....M<br>গ্রাম ডাক্তার (Village doctor) ....N<br>এলোপ্যাথী ঔষধের দোকান (Allopath drug store) ....O<br>ইমাম/ঝাড় ফুক/ওঝা (Spiritual healer) ....P<br>পরিবারের অন্যান্য সদস্য/আত্মীয়/ প্রতিবেশী/বন্ধু Family/relative/Neighbor/friend............................Q<br>অন্যান্য Others___________________________........X<br>(নির্দিষ্ট করুন)<br>জানি না/মনে নাই Don't know/can't remember ....Z	
X13	শেষ গর্ভের সময় বাচ্চা হওয়া এবং জরুরী অবস্থা বিষয়ক পরিকল্পনার জন্য আপনি আপনার স্ত্রীর সাথে আলাপ/পরামর্শ করেছিলেন কি?<br>Did you discuss a plan for birth and emergencies with your wife?	হ্যাঁ Yes .... 1<br>না No .... 2<br>জানি না/ মনে নাই Don't know/Can't remember 9	
X14	ডেলিভারীর সময় ব্যবহারের জন্য শেষবার গর্ভবতী থাকা অবস্থাতেই ডেলিভারী কিট ক্রয় বা সংগ্রহ করে রেখেছিলেন কি?<br>Did you buy / collect delivery kit for using during delivery?	হ্যাঁ Yes .... 1<br>না No .... 2<br>জানি না/ মনে নাই Don't know/Can't remember 9	
X15	ডেলিভারীর সময় যদি কোন অসুবিধা হয়, তাহলে যেন তাড়াতাড়ি হাসপাতাল বা স্বাস্থ্যকেন্দ্রে যাওয়া যায়, সে জন্য আগে থেকেই কি যানবাহনের ব্যবস্থা করে রেখেছিলেন?<br>Did you arrange emergency transport before delivery in case complication during delivery?	হ্যাঁ Yes .... 1<br>না No .... 2<br>জানি না/ মনে নাই Don't know/Can't remember 9	
X16	ডেলিভারীর সময়টায় টাকা পয়সা লাগতে পারে, তাই আগে থেকেই টাকা পয়সা জমা করে রেখেছিলেন কি?<br>Did you save money for emergency need during delivery?	হ্যাঁ Yes .... 1<br>না No .... 2<br>জানি না/ মনে নাই Don't know/Can't remember 9	
X17	ডেলিভারীর সময় যদি রক্তের প্রয়োজন হয়, সে জন্য আগে থেকেই কি একজন রক্তদাতা নির্বাচন করে রেখেছিলেন?<br>Did you identify a potential blood donor before delivery ?	হ্যাঁ Yes .... 1<br>না No .... 2<br>জানি না/ মনে নাই Don't know/Can't remember 9	2→ X19<br>9→ X19

No.	Questions And Filters	Coding Categories	Skip
	→Supp Mod X X00a, X00b, X00c)		
X18	কাকে ঠিক করে রেখেছিলেন?<br><br>Whom did you identify?	একই খানার লোকজন Household Member................ 1<br>রক্ত সম্পর্কের আত্মীয় Blood relative .... 2<br>প্রতিবেশী Neighbour.. 3<br>অপরিচিত ব্যক্তি Unknown person............................ 4<br>অন্যান্য ___________________________ .... 7<br>(নির্দিষ্ট করুন)<br>জানি না/ মনে নাই Don't know/Can't remember 9	
X19	ডেলিভারীর পর পর শিশুটিকে মুড়ানো মোছানোর জন্য কমপক্ষে দুই টুকরা কাপড় কি জোগাড় করে রেখেছিলেন?<br>Did you arrange two pieces of cloth for drying and wrapping the baby?	হ্যাঁ Yes .... 1<br>না No .... 2<br>জানি না/ মনে নাই Don't know/Can't remember 9	
X20	শেষ যখন আপনার স্ত্রী গর্ভবতী ছিলেন, তখন (গর্ভবতী থাকাকালীন সময়ে) আপনার স্ত্রীর এই ডেলিভারীটি কোথায় হবে, সেটি কি ঠিক করে রেখেছিলেন?<br>*[উত্তর 'হ্যাঁ' হলে, জানতে চান,]* কোথায় ডেলিভারী/শিশুটি হবে বলে ঠিক করে রেখেছিলেন?<br>During the last pregnancy of your wife, did you select the place where your child would be deliverd? If yes, ask which place did you select?	বাসায় At Home .... 1<br>স্বাস্থ্যকেন্দ্রে Health Center .... 2<br>আগে থেকে ঠিক করে রাখি নাই Didn't have any plan . 9	
X21	শেষ যখন আপনার স্ত্রী গর্ভবতী ছিলেন, তখন (গর্ভবতী থাকাকালীন সময়ে) আপনার স্ত্রীর ডেলিভারীতে সাহায্য করার জন্য বা ডেলিভারীটি করানোর জন্য বা ডেলিভারীর সময় উপস্থিত থাকার জন্য পছন্দের কাউকে আপনারা ঠিক করে রেখেছিলেন কি?<br>During your wife's most recent pregnancy, did you or your family select someone or a companion of choice to be present for birth/ delivery?	হ্যাঁ Yes .... 1<br>না No .... 2<br>জানি না/ মনে নাই Don't know/Can't remember 9	
X22	মাতৃ স্বাস্থ্যসেবা প্রদানে প্রশিক্ষণহীন দাই এর নতুন ভূমিকা সম্পর্কে আপনি অবগত আছেন কি?<br>Are you aware of the new role of TBAs regarding MNH services?	হ্যাঁ Yes .... 1<br>না No .... 2<br>জানি না/ মনে নাই Don't know/Can't remember 9	→Supp Mod x (X22a)

No.	Questions And Filters	Coding Categories	Skip
	→Supp Mod X X00a, X00b, X00c)		
X23	শেষ গর্ভের সময়, কে ডেলিভারীতে সাহায্য করেছিল?<br><br>*[মহিলার স্বামীকে জিজ্ঞেস করুন]* আরও কেউ? *[মহিলার স্বামীর নিজে থেকে দেয়া সবগুলো উত্তরই বৃত্তায়িত করুন । উত্তরগুলো পড়ে শুনাবেন না। একাধিক উত্তর হতে পারে।]*<br><br>*যদি উত্তরদাত্রী বলেন যে, কেউ ডেলিভারীতে সহায়তা করেননি তাহলে জিজ্ঞাসা করুন, প্রাপ্ত বয়স্ক কেউ ডেলিভারীর সময়ে উপস্থিত ছিল কিনা?*<br><br>Who assisted with the delivery during last pregnancy?<br><br>Anyone else?<br><br>PROBE FOR THE TYPE(S) OF PERSON(S) AND RECORD ALL MENTIONED.<br><br>IF RESPONDENT SAYS NO ONE ASSISTED, PROBE TO DETERMINE WHETHER ANY ADULTS WERE PRESENT AT THE DELIVERY.	**দক্ষ/প্রশিক্ষণ প্রাপ্ত স্বাস্থ্য কর্মী (Medically trained)**<br>পাশ করা ডাক্তার (MBBS doctor)........................ A<br>নার্স/ধাত্রী (Nurse/midwife) .... B<br>প্যারামেডিক/মেডিকেল এসিসটেন্ট/সাকমো (Paramedic/MA/SACMO).............................. C<br>পরিবার কল্যাণ পরিদর্শক (FWV)........................ D<br>সি,এস,বি,এ (CSBA) .... E<br>**অন্যান্য স্বাস্থ্য কর্মী (Other health worker)**<br>স্বাস্থ্য সহকারী/ পরিবার কল্যান সহকারী (HA /FWA) . F<br>পুষ্টি কর্মী (CNP).. G<br>সুসমা কার্যক্রমের স্বেচ্ছাসেবী (Volunteer of SUSOMA prj)H<br>অন্যান্য কমিউনিটি স্বাস্থ্য কর্মী - এনজিও কর্মী, স্বেচ্ছাসেবী (Other CHWs, NGO worker, volunteer)............. I<br>**অন্যান্য (Other)**<br>প্রশিক্ষণ প্রাপ্ত টিবিএ (প্রশিক্ষণ প্রাপ্ত ধন্নী, চাউনী, দাই) (TTBA) .... J<br>প্রশিক্ষণহীন টিবিএ (ধন্নী, চাউনী, দাই) TBA(Dai/Dhorni/Chauni) .... K<br>হোমিওপ্যাথ/হোমিওপ্যাথ ঔষধের দোকান (Homeopath/Homeopath drug store) .... L<br>আয়ুর্বেদিক চিকিৎসক / আয়ুর্বেদিক ঔষধের দোকান /হেকিম/কবিরাজ (Ayurved/ Ayurvedic drug store /Hekim/Kabiraj) .... M<br>গ্রাম ডাক্তার (Village doctor) .... N<br>এলোপ্যাথী ঔষধের দোকান (Allopath drug store) ... O<br>ইমাম/ঝাড় ফুক/ওঝা (Spiritual healer) .... P<br>পরিবারের অন্যান্য সদস্য/আত্মীয়/ প্রতিবেশী/বন্ধু Family/relative/Neighbor/friend....................... Q<br>অন্যান্য Others___________________________... X<br>*(নির্দিষ্ট করুন)*<br>জানি না/মনে নাই Don't know/can't remember .... Z	

No.	Questions And Filters	Coding Categories	Skip
	→Supp Mod X X00a, X00b, X00c)		
X24	আপনার স্ত্রীর শেষ গর্ভের সময়, ডেলিভারীটি কোথায় হয়েছিল?<br><br>*(উত্তরগুলো পড়ে শুনাবেন না)*<br>[কেবলমাত্র একটি উত্তর হবে]<br><br>Where did the most recent delivery take place?<br><br>ONLY ONE ANSWER ALLOWED	**সরকারী স্বাস্থ্য কেন্দ্র (Govt Health center)**<br>মেডিকেল কলেজ হাসপাতাল (Medical College Hospital)....01<br>জেলা/সদর হাসপাতাল (District /Sadar Hospital)...........02<br>মা ও শিশু স্বাস্থ্য কেন্দ্র (MCWC).............................03<br>উপজেলা স্বাস্থ্য কমপেন্ডক্স (UHC)...........................04<br>ইউনিয়ন স্বাস্থ্য ও পরিবার কল্যাণ কেন্দ্র/সাব সেন্টার/আরডি (FWC/SC/RD) ....05<br>কমিউনিটি ক্লিনিক (Community clinic).......................06<br>সেটেলাইট ক্লিনিক/ ইপিআই কেন্দ্র (Satellite clinic/EPI centre)...07<br>অন্যান্য সরকারী স্বাস্থ্য কেন্দ্র (Other Govt Health facility) ..08<br>**বেসরকারী স্বাস্থ্য কেন্দ্র (Non Govt Health center)**<br>এনজিও হাসপাতাল (NGO hospital) ....09<br>এনজিও স্থায়ী স্বাস্থ্য কেন্দ্র (NGO static health centre) ......10<br>এনজিও সেটেলাইট ক্লিনিক (NGO satellite clinic)...........11<br>পুষ্টি কেন্দ্র (NNP centre)......................................12<br>অন্যান্য বেসরকারী স্বাস্থ্য কেন্দ্র (Other NGO Health facility)...13<br>**প্রাইভেট (Private Health sector)**<br>হাসপাতাল/ ক্লিনিক (Hospital/clinic)..........................14<br>স্বাস্থ্য কেন্দ্র /ডিসপেনসারী (Health centre/Dispensary) .....15<br>এমবিবিএস ডাক্তারের চেম্বার (MBBS doctor's chamber) ...16<br>গ্রাম ডাক্তারের চেম্বার (Village doctor's chamber)...........17<br>প্যারামেডিক/মেডিকেল এসিসটেন্ট/সাকমোর চেম্বার (Paramedic/ MA/SACMO chamber) .... 18<br>এলোপ্যাথী ঔষধের দোকান (Allopath drug store) ....19<br>অন্যান্য প্রাইভেট স্বাস্থ্য কেন্দ্র (Other private Health facility)20<br><br>**বাড়ী (Home)**<br>নিজ বাড়ী, স্বামী/শ্বশুড় বাড়ী (Own home, husband/father in laws house)................21<br>বাবার বাড়ী (My natal home) ....22<br><br>অন্যান্য (Others______________________________..97<br>(নির্দিষ্ট করুন)<br>জানি না/মনে নাই Don't know/can't remember 99	
X25	আপনার স্ত্রীর শেষ গর্ভের ডেলিভারীর সময় আপনি আপনার স্ত্রীর সাথে গিয়েছিলেন/ সাথে ছিলেন?<br><br>Did you accompany your wife during birth/delivery of her most recent pregnancy?	হ্যাঁ Yes.. 1<br>না No.. 2	
X26	শেষ গর্ভের ডেলিভারীর সময় নিরাপদ ডেলিভারী কিট ব্যবহার করা হয়েছিল কি?<br><br>Was a clean delivery kit used during delivery of most recent pregnancy?	হ্যাঁ (Yes).. 1<br>না (No) .... 2<br>জানা নাই/মনে নাই DK/can't remember.................. 9	

No.	Questions And Filters	Coding Categories	Skip
	→Supp Mod X X00a, X00b, X00c)		
X27	কখনও কখনও ডেলিভারীরর সময় কিছু বিপদ চিহ্ন দেখা যেতে পারে এবং তখন প্রসবকারী মাকে তাৎক্ষনিকভাবে স্বাস্থকেন্দ্রে পাঠানো দরকার হয়। ডেলিভারীরর সময়ে কি কি বিপদচিহ্ন দেখা দিলে একজন মহিলাকে তাৎক্ষনিকভাবে হাসপাতালে পাঠাতে হয় ?<br><br>*[মহিলার স্বামীকে জিজ্ঞেস করুন]* আরও কোন সমস্যা/জটিলতা? *[মহিলার স্বামীর নিজে থেকে দেয়া সবগুলো উত্তরই বৃত্তায়িত করুন । উত্তরগুলো পড়ে শুনাবেন না। একাধিক উত্তর হতে পারে।]*<br><br>During delivery , once contraction started women may encounter severe problem or illness and should go or be taken immediately to a health facility.<br><br>While having contractions or delivering a baby, what types of symptoms would cause you to seek immediate care at a health facility right away?<br><br>Circle all signs mentioned, but do not prompt with any suggestions.	খিচুঁনী Convulsion.. A<br>তীব্র জ্বর Hight Fever.. B<br>অতিরিক্ত রক্তস্রাব Excessive Vaginal Bleeding....... C<br>দ্রুত শ্বাস /শ্বাস নিতে কষ্ট হওয়া Fast/difficult breathing<br>দুর্গন্ধযুক্ত স্রাব A bad smelling vaginal discharge..... D<br>ফুল না পরা Retained Placenta.......................... E<br>তীব্র মাথা ব্যথা/চোখে ঝাপসা দেখা Severe Headache/blurred vision .... F<br>দীর্ঘ / প্রলম্বিত প্রসব (12 ঘন্টার বেশী) ব্যথা থাকলে prolong labor 12 hr...................................... G<br>শিশুর হাত /পা আগে বের হয়ে এলে Hand or Feet came first.. H<br>অন্যান্য Others____________________________ ..... X<br>(নির্দিষ্ট করুন)<br>জানা নাই/মনে নাই DK/can't remember.................. Z	
X28	ডেলিভারীর পর একজন মায়ের কখনও কখনও এমন কিছু জটিলতা বা বিপদচিহ্ন দেখা যেতে পারে যে তখন তাকে অবিলম্বে চিকিৎসার জন্য হাসপাতালে/ ডাক্তারের কাছে নিয়ে যেতে হয়। ডেলিভারীর পর কি ধরনের লক্ষণ/ জটিলতা বা বিপদচিহ্ন দেখলে আপনি একজন মাকে চিকিৎসার জন্য হাসপাতালে বা স্বাস্থ্যকর্মীর কাছে নিয়ে যাবেন?<br><br>*[মহিলার স্বামীকে জিজ্ঞেস করুন]* আরও কোন সমস্যা/জটিলতা? *[মহিলার স্বামীর নিজে থেকে দেয়া সবগুলো উত্তরই বৃত্তায়িত করুন । উত্তরগুলো পড়ে শুনাবেন না। একাধিক উত্তর হতে পারে।]*<br><br>Sometimes mothers after delivery may have severe illness and should be taken immediately to a health facility.<br><br>What type of symptoms would cause you to go to a health facility right away?<br><br>Ask : Anything else?<br><br>CIRCLE ALL SIGNS MENTIONED, BUT DO NOT PROMPT	যোনীপথে অতিরিক্ত রক্তস্রাব<br>Excessive vaginal bleeding .... A<br>শ্বাস নিতে কষ্ট/ দ্রুত শ্বাস<br>Difficult breathing/ fast breathing .... B<br>অতিরিক্ত জ্বর হলে High fever .... C<br>পেটে তীব্র ব্যথা Severe abdominal pain................... D<br>তীব্র মাথা ব্যথা/ঝাপসা দেখা<br>Severe headache/blurry vision .... E<br>খিঁচুনী / অজ্ঞান Convulsion/loss of conciousness..... F<br>যোনীপথে দূর্গন্ধযুক্ত স্রাব<br>Foul smelling vaginal discharge............................. G<br>পায়ের পিছনে ব্যথা Pain in calf .... H<br>আচরনগত পরিবর্তন অর্থাৎ যেখানে মা নিজেকে বা বাচ্চাকে আঘাত করতে পারে Behavior that indicates she may hurt herself or the baby............... I<br>স্তন/স্তনবৃন্তে ফোলা, লাল হওয়া বা ব্যথা<br>swollen, red, tender breasts or nipples.................... J<br>প্রস্রাব করায় অসুবিধা বা প্রস্রাব ঝরা<br>problems urinating, or leaking .... K<br>পেরিনিয়াম এ ব্যথা/ইনফেকশন<br>increased pain or infection in perineum .... L<br>ক্ষতস্থানে ব্যাথা/লাল ভাব/ পুঁজ হওয়া<br>infection in the area of wound<br>(redness, swelling, pain, or pus in wound site .... M<br>অন্য কোন সমস্যা others__________________......... X<br>নির্দিষ্ট করুন<br>জানা নাই/মনে নাই DK/can't remember.................. Z	

No.	Questions And Filters	Coding Categories	Skip
	→Supp Mod X X00a, X00b, X00c)		
X29	আপনার স্ত্রীর শেষ গর্ভের গর্ভকালীন সময়ে, ডেলিভারীর সময় বা ডেলিভারীর পর আপনার স্ত্রীর কি ______ হয়েছিল?<br>(সমস্যা)<br>প্রত্যেকটি সমস্যা পড়ে শুনান:<br>a. তীব্র মাথা ব্যথাসহ চোখে ঝাপসা দেখা<br>b. খিচুনী/একলামশিয়া/অজ্ঞান হওয়া<br>c. উচ্চ রক্তচাপ<br>d. অতিরিক্ত রক্তস্রাব<br>e. পানি ভাঙ্গার ৬ ঘন্টা পরও প্রসব না হওয়া<br>f. বাচ্চার মাথা ছাড়া শরীরের অন্য অংশ আগে আসা<br>g. ১২ ঘন্টার অধিক প্রসব ব্যথা<br>h. ফুল না পড়া<br>i. দুর্গন্ধযুক্ত স্রাবের সাথে তীব্র জ্বর<br>j. হাতে/পায়ে/শরীরে পানি আসা<br>y. উপরে উলিণ্ডখিত সমস্যাগুলোর কোনটাই হয়নি<br><br>Did your wife have any of the following problems during her recent pregnancy, delivery or period after delivery?<br><br>Read aloud the responses:<br><br>a. Blurred vision with severe headache<br>b. Convulsion/eclampsia/unconscious<br>c. High blood pressure<br>d. Excessive vaginal bleeding<br>e. Not delivered even after 6 hours of membrane rupture<br>f. Delivery of parts of the baby other than head<br>g. More than 12 hours of labor pain<br>h. Retained placenta<br>i. Foul smelling discharge with fever<br>j. Edema of hand/feet/body<br>y. None of the above mentined Problems	P= গর্ভাবস্থা (Pregnancy) , D= ডেলিভারীর সময় (Delivery), AD= ডেলিভারীর পর (After delivery)<br>সমস্যা P D AD<br>তীব্র মাথা ব্যথাসহ চোখে ঝাপসা দেখা<br>Blurred vision with severe headache ....A1........A2........A3<br>খিচুনী/একলামশিয়া/অজ্ঞান হওয়া<br>Convulsion/eclampsia/unconscious ....B1........B2........B3<br>উচ্চ রক্তচাপ<br>High blood pressure ....C1 ...... C2 ...... C3<br>অতিরিক্ত রক্তস্রাব<br>Excessive vaginal bleeding ....D1 ...... D2...... D3<br>পানি ভাঙ্গার ৬ ঘন্টা পরও প্রসব না হওয়া Not delivered even after 6 hours of membrane rupture ....E1 ..... -- ....--<br>বাচ্চার মাথা ছাড়া শরীরের অন্য অংশ আগে আসা Delivery of parts of the baby other than head..................--...........F2.........--<br>12 ঘন্টার অধিক প্রসব ব্যথা<br>More than 12 hours of labor pain.........--...........G2........--<br>ফুল না পড়া<br>Retained placenta ....--......... H2...... H3<br>দুর্গন্ধযুক্ত স্রাবের সাথে তীব্র জ্বর<br>Foul smelling discharge with fever--......... --......... I3<br>হাতে/পায়ে/শরীরে পানি আসা<br>Edema of hand/feet/body ....J1........J2........ J3<br>উপরে উলি- খিত সমস্যাগুলোর কোনটাই হয়নি None of the above mentined Problems ....Y1...... Y2...... Y3	→ X34
X30	আপনার স্ত্রীর এই সমস্যা/জটিলতার জন্য আপনি কি কাউকে দেখিয়েছিলেন বা কারও সাহায্য নিয়েছিলেন?<br>Did you seek any sort of treatment for this problem/complication?	হ্যাঁ Yes .... 1<br>না No... 2<br>জানি না/ মনে নাই Don't know/Can't remember 9	 → X34<br>→ X34

No.	Questions And Filters	Coding Categories	Skip
	→Supp Mod X X00a, X00b, X00c)		
X31	আপনার স্ত্রীকে এই সমস্যা/জটিলতার জন্য আপনি কাকে দেখিয়েছিলেন বা কার সাহায্য নিয়েছিলেন?<br><br>*[মহিলার স্বামীকে জিজ্ঞেস করুন]* আরও কেউ? *[মহিলার স্বামীর নিজে থেকে দেয়া সবগুলো উত্তরই বৃত্তায়িত করুন । উত্তরগুলো পড়ে শুনাবেন না। একাধিক উত্তর হতে পারে।]*<br><br>From whom did you seek treatment for this problem/complication? Do not read out the answers. Ask: Anything else? Circle all the answers.	**দক্ষ/প্রশিক্ষণ প্রাপ্ত স্বাস্থ্য কর্মী (Medically trained)**<br>পাশ করা ডাক্তার (MBBS doctor)........................ A<br>নার্স/ধাত্রী (Nurse/midwife) .... B<br>প্যারামেডিক/মেডিকেল এসিসটেন্ট/সাকমো (Paramedic/MA/SACMO)............................... C<br>পরিবার কল্যাণ পরিদর্শক (FWV)......................... D<br>সি,এস,বি,এ (CSBA) .... E<br>**অন্যান্য স্বাস্থ্য কর্মী (Other health worker)**<br>স্বাস্থ্য সহকারী/ পরিবার কল্যান সহকারী (HA /FWA) . F<br>পুষ্টি কর্মী (CNP).. G<br>সুসমা কার্যক্রমের স্বেচ্ছাসেবী (Volunteer of SUSOMA prj)H<br>অন্যান্য কমিউনিটি স্বাস্থ্য কর্মী - এনজিও কর্মী, স্বেচ্ছাসেবী (Other CHWs, NGO worker, volunteer)............. I<br>**অন্যান্য (Other)**<br>প্রশিক্ষণ প্রাপ্ত টিবিএ (প্রশিক্ষণ প্রাপ্ত ধন্নী, চাউনী, দাই) (TTBA) .... J<br>প্রশিক্ষণহীন টিবিএ (ধন্নী, চাউনী, দাই) TBA(Dai/Dhorni/Chauni) .... K<br>হোমিওপ্যাথ/হোমিওপ্যাথ ঔষধের দোকান (Homeopath/Homeopath drug store) .... L<br>আয়ুর্বেদিক চিকিৎসক / আয়ুর্বেদিক ঔষধের দোকান /হেকিম/কবিরাজ (Ayurved/ Ayurvedic drug store /Hekim/Kabiraj) .... M<br>গ্রাম ডাক্তার (Village doctor) .... N<br>এলোপ্যাথী ঔষধের দোকান (Allopath drug store) ... O<br>ইমাম/ঝাড় ফুক/ওঝা (Spiritual healer) .... P<br>পরিবারের অন্যান্য সদস্য/আত্মীয়/ প্রতিবেশী/বন্ধু Family/relative/Neighbor/friend....................... Q<br>অন্যান্য Others__________________________... X<br>(নির্দিষ্ট করুন)<br>জানি না/মনে নাই Don't know/can't remember .... Z	

No.	Questions And Filters	Coding Categories	Skip
	→Supp Mod X X00a, X00b, X00c)		
X32	আপনার স্ত্রীকে এই সমস্যা/জটিলতার জন্য আপনি কোথায় দেখিয়েছিলেন বা কোথায় সেবা পেয়েছিলেন?<br><br>*[মহিলার স্বামীকে জিজ্ঞেস করুন]* আরও কোথাও?<br><br>*[মহিলার স্বামীর নিজে থেকে দেয়া সবগুলো উত্তরই বৃত্তায়িত করুন । উত্তরগুলো পড়ে শুনাবেন না। একাধিক উত্তর হতে পারে।]*<br>*[মহিলা যেখান থেকে সেবা পেয়েছেন, সেই স্বাস্থ্যকেন্দ্রের নাম লিখুন। যদি একাধিক জায়গা থেকে সেবা নিয়ে থাকেন, তাহলে সবগুলো জায়গারই নাম এবং কোড লিখুন।]*<br><br>Code :|____| নাম Name: ______________________________<br><br>Code :|____| নাম Name: ______________________________<br><br>Code :|____| নাম Name: ______________________________<br><br>Where did you go to seek care for this problem/complication?<br>Do not read out the answers. Ask: Anything else?<br>Circle all the answers. Write down code and name of place.	**সরকারী স্বাস্থ্য কেন্দ্র (Govt Health center)**<br>মেডিকেল কলেজ হাসপাতাল (Medical College Hospital)....A<br>জেলা/সদর হাসপাতাল (District /Sadar Hospital)...........B<br>মা ও শিশু স্বাস্থ্য কেন্দ্র (MCWC).............................C<br>উপজেলা স্বাস্থ্য কমপ্লেক্স (UHC)...........................D<br>ইউনিয়ন স্বাস্থ্য ও পরিবার কল্যাণ কেন্দ্র/সাব সেন্টার/আরডি (FWC/SC/RD)...E<br>কমিউনিটি ক্লিনিক (Community clinic).......................F<br>সেটেলাইট ক্লিনিক/ ইপিআই কেন্দ্র (Satellite clinic/EPI centre)...G<br>অন্যান্য সরকারী স্বাস্থ্য কেন্দ্র (Other Govt Health facility) ..H<br>**বেসরকারী স্বাস্থ্য কেন্দ্র (Non Govt Health center)**<br>এনজিও হাসপাতাল (NGO hospital).........................I<br>এনজিও স্থায়ী স্বাস্থ্য কেন্দ্র (NGO static health centre) ......J<br>এনজিও সেটেলাইট ক্লিনিক (NGO satellite clinic)...........K<br>পুষ্টি কেন্দ্র (NNP centre)......................................L<br>অন্যান্য বেসরকারী স্বাস্থ্য কেন্দ্র (Other NGO Health facility)..M<br>**প্রাইভেট (Private Health sector)**<br>হাসপাতাল/ ক্লিনিক (Hospital/clinic).........................N<br>স্বাস্থ্য কেন্দ্র /ডিসপেনসারী (Health centre/Dispensary) .....O<br>এমবিবিএস ডাক্তারের চেম্বার (MBBS doctor's chamber) ...P<br>গ্রাম ডাক্তারের চেম্বার (Village doctor's chamber)...........Q<br>প্যারামেডিক/মেডিকেল এসিসটেন্ট/সাকমোর চেম্বার (Paramedic/ MA/SACMO chamber)..R<br>এলোপ্যাথী ঔষধের দোকান (Allopath drug store) ....S<br>অন্যান্য প্রাইভেট স্বাস্থ্য কেন্দ্র (Other private Health facility)T<br><br>**বাড়ী (Home)**<br>নিজ বাড়ী, স্বামী/শ্বশুড় বাড়ী (Own home, husband/father in laws house)...U<br>বাবার বাড়ী (My natal home) ....V<br><br>অন্যান্য (Others______________________________..X<br>(নির্দিষ্ট করুন)<br>জানি না/মনে নাই Don't know/can't remember ....Z	→ X34<br>→ X34
X33	আপনার স্ত্রীর এই সমস্যা/জটিলতার জন্য সেবা নিতে যাওয়ার সময় আপনি যানবাহন পেতে সহায়তা করেছিলেন কি ?<br>Did you assist your wife in arranging transportation to reach a health facility for this problem/complication?	হ্যাঁ Yes .... 1<br>না No .... 2<br>প্রযোজ্য নয় Not applicable..................................... 9	
X34	আপনার স্ত্রীর শেষ গর্ভের সময়ে, শিশু-মাতৃ স্বাস্থ্য বিষয়ক সেবা পাওয়ার ব্যাপারে স্বাস্থ্যসেবাদানকারীর সাথে আলাপচারিতায়/ব্যবহারে/আচরণে আপনি কি সন্তুষ্ট?<br>Are you satisfied with the interactions with health care providers when receiving MNH services?	হ্যাঁ Yes .... 1<br>না No .... 2<br>প্রযোজ্য নয়... 7<br>জানি না/ মনে নাই Don't know/Can't remember 9	

No.	Questions And Filters	Coding Categories	Skip
	→Supp Mod X X00a, X00b, X00c)		
X35	আপনার স্ত্রীর শেষ গর্ভের ডেলিভারীর পর কোন স্বাস্থ্যকর্মী বা কোন প্রশিক্ষনহীন দাই স্বাস্থ্যকেন্দ্রে বা বাড়ীতে বা অন্য কোন স্থানে আপনার স্ত্রীর স্বাস্থ্য পরীক্ষা করেছিলেন কি?<br><br>Did a health care provider or a traditional birth attendant check on your wife's health after the delivery of her recent pregnancy , either at a health facility , home or other location?	হ্যাঁ Yes.. 1<br>না No .... 2<br>জানি না Don't know/can't remember................. 9	 → X40<br>→ X40
X36	ডেলিভারীর কত সময় পর প্রথমবার আপনার স্ত্রীর স্বাস্থ্য পরীক্ষা করানো হয়েছিল?<br>*[যদি এক দিনের কম হয়, তাহলে 0 বৃত্তায়িত করে ঘন্টায় রেকর্ড করুন, যদি এক থেকে ছয় দিন হয়, তাহলে 1 বৃত্তায়িত করে দিনে রেকর্ড কর ন , যদি ছয় দিনের বেশী হয়, তাহলে 2 বৃত্তায়িত করে সপ্তাহে রেকর্ড কর ন]*<br><br>How long after the delivery did the first health check took place?<br><br>If less than one day, circle 0 and record hours; if one to six days circle 1 and record days; if more than 6 days circle 2 and record weeks.	ঘন্টা Hours ....0 |___|___| <br>দিন Days......................................1 |___|___| <br>সপ্তাহ Weeks ....2 |___|___| <br>জানি না Don't know/can't remember................. 999	
X37	শেষ গর্ভের সময়, জন্মের/ ডেলিভারীর পর কে আপনার স্ত্রীর স্বাস্থ্য পরীক্ষা করেছিল?<br><br>*[মহিলার স্বামীকে জিজ্ঞেস কর ন]* আরও কেউ? *[মহিলার স্বামীর নিজে থেকে দেয়া সবগুলো উত্তরই বৃত্তায়িত কর ন । উত্তরগুলো পড়ে শুনাবেন না। একাধিক উত্তর হতে পারে।]*<br><br>Who checked your wife's health at that time?<br><br>Do not read out the answers.<br>ASK: Anyone else?<br>Circle and write down all the answers	**<u>দক্ষ/প্রশিক্ষণ প্রাপ্ত স্বাস্থ্য কর্মী (Medically trained)</u>**<br>পাশ করা ডাক্তার (MBBS doctor)........................ A<br>নার্স/ধাত্রী (Nurse/midwife) .... B<br>প্যারামেডিক/মেডিকেল এসিসটেন্ট/সাকমো (Paramedic/MA/SACMO)............................... C<br>পরিবার কল্যাণ পরিদর্শক (FWV)......................... D<br>সি,এস,বি,এ (CSBA) .... E<br>**<u>অন্যান্য স্বাস্থ্য কর্মী (Other health worker)</u>**<br>স্বাস্থ্য সহকারী/ পরিবার কল্যান সহকারী (HA /FWA) . F<br>পুষ্টি কর্মী (CNP).. G<br>সুসমা কার্যক্রমের স্বেচ্ছাসেবী (Volunteer of SUSOMA prj)H<br>অন্যান্য কমিউনিটি স্বাস্থ্য কর্মী - এনজিও কর্মী, স্বেচ্ছাসেবী (Other CHWs, NGO worker, volunteer).............. I<br>**<u>অন্যান্য (Other)</u>**<br>প্রশিক্ষণ প্রাপ্ত টিবিএ (প্রশিক্ষণ প্রাপ্ত ধন্নী, চাউনী, দাই) (TTBA) .... J<br>প্রশিক্ষণহীন টিবিএ (ধন্নী, চাউনী, দাই) TBA(Dai/Dhorni/Chauni) .... K<br>হোমিওপ্যাথ/হোমিওপ্যাথ ঔষধের দোকান (Homeopath/Homeopath drug store) .... L<br>আয়ুর্বেদিক চিকিৎসক / আয়ুর্বেদিক ঔষধের দোকান /হেকিম/কবিরাজ (Ayurved/ Ayurvedic drug store /Hekim/Kabiraj) .... M<br>গ্রাম ডাক্তার (Village doctor) .... N<br>এলোপ্যাথী ঔষধের দোকান (Allopath drug store) ... O<br>ইমাম/ঝাড় ফুক/ওঝা (Spiritual healer) .... P<br>পরিবারের অন্যান্য সদস্য/আত্মীয়/ প্রতিবেশী/বন্ধু Family/relative/Neighbor/friend........................ Q<br>অন্যান্য Others___________________________... X<br>*(নির্দিষ্ট কর ন)*<br>জানি না/মনে নাই Don't know/can't remember .... Z	

No.	Questions And Filters	Coding Categories	Skip
	→Supp Mod X X00a, X00b, X00c)		
X38	শেষ গর্ভের জন্মের/ ডেলিভারীর পর আপনার স্ত্রীর মেডিকেল চেক-আপের জন্য আপনি কোথায় দেখিয়েছিলেন/কোথায় করিয়েছিলেন?<br><br>Where did your wife receive a health check after the delivery of her most recent pregnancy?	**সরকারী স্বাস্থ্য কেন্দ্র (Govt Health center)**<br>মেডিকেল কলেজ হাসপাতাল (Medical College Hospital)....A<br>জেলা/সদর হাসপাতাল (District /Sadar Hospital)...........B<br>মা ও শিশু স্বাস্থ্য কেন্দ্র (MCWC).............................C<br>উপজেলা স্বাস্থ্য কমপ্লেক্স (UHC)...........................D<br>ইউনিয়ন স্বাস্থ্য ও পরিবার কল্যাণ কেন্দ্র/সাব সেন্টার/আরডি (FWC/SC/RD)..E<br>কমিউনিটি ক্লিনিক (Community clinic).......................F<br>সেটেলাইট ক্লিনিক/ ইপিআই কেন্দ্র (Satellite clinic/EPI centre)..............................G<br>অন্যান্য সরকারী স্বাস্থ্য কেন্দ্র (Other Govt Health facility) ..H<br>**বেসরকারী স্বাস্থ্য কেন্দ্র (Non Govt Health center)**<br>এনজিও হাসপাতাল (NGO hospital) ....I<br>এনজিও স্থায়ী স্বাস্থ্য কেন্দ্র (NGO static health centre) ......J<br>এনজিও সেটেলাইট ক্লিনিক (NGO satellite clinic)...........K<br>পুষ্টি কেন্দ্র (NNP centre)......................................L<br>অন্যান্য বেসরকারী স্বাস্থ্য কেন্দ্র (Other NGO Health facility)..............................M<br>**প্রাইভেট (Private Health sector)**<br>হাসপাতাল/ ক্লিনিক (Hospital/clinic).........................N<br>স্বাস্থ্য কেন্দ্র /ডিসপেনসারী (Health centre/Dispensary) .....O<br>এমবিবিএস ডাক্তারের চেম্বার (MBBS doctor's chamber) ...P<br>গ্রাম ডাক্তারের চেম্বার (Village doctor's chamber)...........Q<br>প্যারামেডিক/মেডিকেল এসিসটেন্ট/সাকমোর চেম্বার (Paramedic/ MA/SACMO chamber).. R<br>এলোপ্যাথী ঔষধের দোকান (Allopath drug store) ....S<br>অন্যান্য প্রাইভেট স্বাস্থ্য কেন্দ্র (Other private Health facility)T<br>**বাড়ী (Home)**<br>নিজ বাড়ী, স্বামী/শ্বশুড় বাড়ী (Own home, husband/father in laws house)..U<br>বাবার বাড়ী (My natal home) ....V<br><br>অন্যান্য (Others_______________________________..X<br>(নির্দিষ্ট করুন)<br>জানি না/মনে নাই Don't know/can't remember ....Z	→ X40<br>→ X40
X39	শেষ গর্ভের জন্মের/ ডেলিভারীর পর আপনার স্ত্রীর মেডিকেল চেক-আপের জন্য আপনি আপনার স্ত্রীর সাথে গিয়েছিলেন?<br><br>Did you accompany your wife while receiving a health check after the delivery of her most recent pregnancy?	হ্যাঁ Yes... 1<br>না No.. 2	**→Supp Mod x, (X 39a, X39b, X39c, X39d)**

No.	Questions And Filters	Coding Categories	Skip
	→Supp Mod X X00a, X00b, X00c)		
X40	কখনও কখনও নবজাতক বাচ্চা একমাস বয়সের মধ্যেই খুব অসুস্থ হয়ে যায় এবং তখন তাদেরকে অবিলম্বে চিকিৎসার জন্য হাসপাতালে/ ডাক্তারের কাছে নিয়ে যেতে হয়। কি ধরনের লক্ষণ দেখলে আপনি আপনার নবজাতককে সাথে সাথেই চিকিৎসার জন্য হাসপাতালে বা ডাক্তারের কাছে নিয়ে যাবেন?<br><br>*[মহিলার স্বামীকে জিজ্ঞেস কর—ন]* আরও কোন সমস্যা/জটিলতা? *[মহিলার স্বামীর নিজে থেকে দেয়া সবগুলো উত্তরই বৃত্তায়িত কর—ন । উত্তরগুলো পড়ে শুনাবেন না। একাধিক উত্তর হতে পারে।]*<br><br>Sometimes newborns, within the first month of life, have severe illness and should be taken immediately to a health facility.<br><br>What types of symptoms would cause you to take a newborn to a health facility right away?<br><br>ASK: Anything else?<br><br>Do not read responses. Record all that are mentioned.	খিঁচুনি বা শরীর শক্ত হওয়া Convulsions..................... A<br>জ্বর/বাচ্চার শরীর গরম হওয়া Baby feels hot/Fever ... B<br>বুকের দুধ চুষতে না পারা Poor suckling or feeding ... C<br>কষ্টকর/দ্রুত শ্বাস নেয়া Difficult or fast breathing.... D<br>বাচ্চার শরীর ঠান্ডা হওয়া Baby feels cold .... E<br>বাচ্চা খুব ছোট হওয়া Too small baby/too early......... F<br>হাতের তালু/ পায়ের পাতা/চোখ হলুদ হওয়া/জন্ডিস<br>Yellow palm/soles/eye color (jaundice) .... G<br>পেট ফাঁপা বা ফোলা Sowllen abdomen .... H<br>অজ্ঞান/হুঁশ না থাকা Unconsciousness .... I<br>নাভির চারপাশ,চোখ অথবা চামড়া লাল হওয়া/পুঁজ বের হওয়া Pus or redness of the umbilical stump,eyes or skin...<br>রক্তপাত Bleeding...<br>ডায়রিয়া Diarrhoea...<br>মুখের ভিতর ঘা/সাদা দাগ<br>Ulcers or thrush (white patches in mouth)............... M<br>অন্যান্য Others ____________________________... X<br>(নির্দিষ্ট কর—ন)<br>জানা নাই (Don't know).................................... Z	

Section Y: People's Institution Group

This section contains some information about People's Institution group

আপনি হয়ত জানেন যে কখনও কখনও এলাকা ভিত্তিক কিছু কমিটি/দল থাকে। আমি এখন এসব বিষয়ে কিছু প্রশ্ন করব।

You may know that there are local committees in many communities. Now I will discuss about such commnitte / group in your area.

No.	Questions And Filters	Coding Categories	Skip
Y01	আপনার এলাকায় এমন কোন কমিটি বা দল (গ্র—প) সম্পর্কে আপনি জানেন কি যারা মা ও শিশুর স্বাস্থ্যের উন্নতির জন্য কাজ করে?<br>Do you know about any committees, or network or group in your community that works towards improving maternal and child health?	হ্যাঁ Yes .... 1<br>না No 2	 → Y12
Y02	আপনি বা আপনার পরিবারের কোন সদস্য কি এই ধরনের কমিটি বা দল (গ্র—প) এর সদস্য?<br>Are you and/or any of your family member is a member of any such group?	হ্যাঁ Yes .... 1<br>না No 2	 → Y04
Y03	আপনি/আপনার পরিবারের সদস্য কতদিন আগে ঐ কমিটি বা দলের (গ্র—পের) সদস্য হয়েছেন? [বছরে বললে মাসে পরিবর্তিত কর—ন। ১ মাসের কম হলে ০০ লিখুন]<br>How long ago did you or someone in your family become a member of this group?<br>Interviewer: If respondent answer in years convert it into months. If less than one month write '00' in the box	মাস MONTH... |___|___| মনে নাই Don't Remember... 99	

No.	Questions And Filters	Coding Categories	Skip
Y04	এই কমিটি বা গ্রুপ কোন কোন বিষয় নিয়ে কাজ করে?<br>What issues does the group deal with?	মায়ের স্বাস্থ্য সেবা Mother's health ....A<br>শিশুর স্বাস্থ্য সেবা Baby's health....................................B<br>পরিবার পরিকল্পনা Family planning.................................C<br>শিক্ষা Education...D<br>অসুস্থ মা ও শিশুর পরিবহনের ব্যবস্থা করে<br>Arrange transportation for sick mother & babies .... E<br>অসুস্থ মা ও শিশুর চিকিৎসার জন্য অর্থের ব্যবস্থা করে<br>Financing sick mother & babies ....F<br>অন্যান্য Others________________________________.....X<br>নির্দিষ্ট করুন<br>জানে না Don't know .... Z	
Y05	এই কমিটি /দলের কাছে আপনি কি মায়ের অথবা শিশুর স্বাস্থ্য সম্পর্কিতকোন প্রকার সমস্যার কারনে সাহায্যের জন্য গিয়েছিলেন?<br>Did you ever go for any sort of help from this group for maternal or child health related problems?	হ্যাঁ Yes ....1<br>না No 2	
Y06	এই কমিটি/দলের কাছ থেকে আপনি কি কখনও মায়ের অথবা শিশুর স্বাস্থ্য সম্পর্কিত কোন প্রকার সমস্যার কারনে কোন ধরনের সাহায্য পেয়েছিলেন?<br>Have you ever received any sort of help from this group for maternal or child health related problems?	হ্যাঁ Yes ....1<br>না No 2	→ Y12
Y07	এই কমিটি/দলের কাছ থেকে মায়ের অথবা শিশুর স্বাস্থ্য সম্পর্কিতকোন প্রকার সমস্যার কারনে কি ধরনের সাহায্য পেয়েছিলেন?<br>What kind of support did you receive from this group for maternal or child health related problems?	পরিবহনের ব্যবস্থা করেছিল Arranged transportation ....A<br>টাকা পয়সার ব্যবস্থা করেছিল Aranged financial support ....B<br>স্বাস্থ্যকর্মীকে জানানো Informed health worker ....C<br>স্বাস্থ্যকর্মীর পরিদর্শন নিশ্চিত করা Ensured visit of<br>health worker ....D<br>স্বাস্থ্যকেন্দ্র থেকে সেবা পাওয়ার ক্ষেত্রে সহায়তা করেছিল<br>Ensured service from/linkage with health facility .... E<br>অন্যান্য Other________________________________......X	
Y08	***[Y 07 এ A বৃত্তায়িত হলে]*** এই কমিটি/গ্রুপের কাছ থেকে কি ধরনের পরিবহন সুবিধা পেয়েছিলেন?<br>**If Y07 A is circled**, ask, what kind of support for transportation did you receive from the group?	যানবাহন খুঁজতে সাহায্য করেছে Helped in finding a vehicle......1<br>যানবাহন দিয়েছে Provided with a vehicle ....2<br>প্রযোজ্য নয় Not applicable 3	
Y09	***[Y 07 এ B বৃত্তায়িত হলে]*** তাদের তহবিল থেকে সরাসরি আপনাকে টাকা দিয়েছিল না-কি এলাকা থেকে টাকা তুলে দিয়েছিল?<br>**If Y07 B is circled,** ask did they give you money directly from their fund or arranged money from the community?	তহবিল থেকে দিয়েছিল Gave money from their fund ....1<br>এলাকা থেকে টাকা তুলে দিয়েছিল Arranged from community ... 2<br>উভয়েই Both ....3<br>প্রযোজ্য নয় Not applicable ....3<br>জানি না Don't know..9	
Y10	***[Y 07 এ E বৃত্তায়িত হলে*** এই কমিটি/গ্রুপের কাছ থেকে **স্বাস্থ্যকেন্দ্র হতে সেবা পাওয়ার ক্ষেত্রে** কি ধরনের **সহায়তা** পেয়েছিলেন?<br>**If Y07 E is circled**, ask, what kind of support for service/linkage did you receive from the group?	________________________________|____|____| ________________________________|____|____| প্রযোজ্য নয় Not applicable 3	
Y11	এই কমিটি/দলের কাছ থেকে মায়ের অথবা শিশুর স্বাস্থ্য সম্পর্কিত সমস্যার কারনে যে সাহায্য পেয়েছিলেন তা কি আপনার কাছে উপকারী মনে হয়েছে?<br>Do you consider the help that you received for maternal or child health related problems as beneficial?	হ্যাঁ Yes ....1<br>না No 2	
Y12	উত্তর দাতাকে ধন্যবাদ জানিয়ে সাক্ষাৎকার শেষ করুন।<br>Thanks the mother for providing time and complete the interview.		
