# Supplementary material for: Birth preparedness and complication readiness among women and couples and its association with skilled birth attendance in rural Bangladesh
Source: PLoS One. 2018 Jun 7;13(6):e0197693. doi: 10.1371/journal.pone.0197693 (PMC5991697; doi:10.1371/journal.pone.0197693)
Supplement: S2 Table — (DOCX) [file pone.0197693.s002.docx]

S2 Table: Associations between birth with an SBA (primary outcome of interest) and other covariates using bivariate logistic regression

| **Background characteristics of Women** | **Birth with an SBA (N=317)** |
| --- | --- |
|  | **UOR  (95% CI)** |
| **Women’s age** |  |
| 15-24 years | Reff |
| 25-44 year | 0.45* (0.23, 0.86) |
| 35+ years | 0.70 (0.24, 1.98) |
| **Women’s education** |  |
| Primary incomplete (0-4 years) | Reff |
| Primary complete to secondary incomplete (5-9 years) | 1.63 (0.84, 3.19) |
| Secondary complete or higher (10+ years) | 13.7* (4.83, 38.86) |
| **Husbands’ education** |  |
| Primary incomplete (0-4 years) | Reff |
| Primary complete to secondary incomplete (5-9 years) | 2.19* (1.12, 4.26) |
| Secondary complete or higher (10+ years) | 13.62* (4.53, 40.96) |
| **Religion** |  |
| Muslim | Reff |
| Others (Hindu/ Christian etc.) | 5.38* (2.32, 12.46) |
| **Wealth quintile** |  |
| Lowest | Reff |
| Second | 0.51 (0.19, 1.77) |
| Middle | 1.18 (0.43, 3.20) |
| Fourth | 0.56 (0.18, 1.76) |
| Highest | 3.56* (1.52, 8.37) |

*p<0.05
